# Supplementary material for: Automatic deep learning-driven label-free image-guided patch clamp system
Source: Nat Commun. 2021 Feb 10;12:936. doi: 10.1038/s41467-021-21291-4 (PMC7875980; doi:10.1038/s41467-021-21291-4)
Supplement: Supplementary file 1 — Supplementary Information [file 41467_2021_21291_MOESM1_ESM.pdf]

Supplementary Information

Automatic deep learning-driven label-free image-guided patch clamp system

Krisztian Koos[1], Gáspár Oláh[2], Tamas Balassa[1], Norbert Mihut[2], Márton Rózsa[2], Attila Ozsvár[2], Ervin Tasnadi[1], Pál Barzó[3], Nóra Faragó[2,4,5], László Puskás[4,5], Gábor Molnár[2], József Molnár[1], Gábor Tamás[2], Peter Horvath[1,6*]

[1] Laboratory of Microscopic Image Analysis and Machine Learning, Institute of Biochemistry, Biological Research Centre, Eötvös Loránd Research Network (ELKH), Szeged, Hungary
[2] MTA-SZTE Research Group for Cortical Microcircuits of the Hungarian Academy of Sciences, Department of Physiology, Anatomy and Neuroscience, University of Szeged, Hungary
[3] Department of Neurosurgery, University of Szeged, Szeged, Hungary
[4] Laboratory of Functional Genomics, Institute of Genetics, Biological Research Centre, Eötvös Loránd Research Network (ELKH), Szeged, Hungary
[5] Avidin Ltd, Szeged, Hungary
[6] Institute for Molecular Medicine Finland, University of Helsinki, Helsinki, Finland

These authors contributed equally: K.K. and G.O.
Correspondence should be addressed to P.H. (email: horvath.peter@brc.hu).

Table of Contents

Pipette Detection System	3
Deep Learning Model Comparison for Cell Detection	9
Cell Tracking System	23
Pressure Regulator Setup	26
Electrophysiology Stimuli for DIGAP	27
Software Usage and Parameters	31
Representative Examples	50
Anatomical Reconstruction Examples	65
References	73

Supplementary Note 1: Pipette Detection System

Introduction

The pipette detection pipeline consists of three steps. First, when the pipette is visible in the image, an image stack is acquired. Ideally, the pipette should cover most of the image and should be nearly in focus. Moreover, the tip should be near the sample so that light conditions during detection are similar to those during the movement of the pipette in the sample. Then a fast initialization algorithm is run on the acquired stack. The initialization is based on a moving line profile in the image and is detailed in Section . Finally, the result of the initialization is refined using the main tip detection algorithm referred to as Pipette Hunter 3D (PH3D). The algorithm is the 3-dimensional extension of the base Pipette Hunter model [1] and the derivations are detailed in Section . The energy function is minimized using the variational method and gradient descent. Section describes the performed quality measurement and comparison of the algorithms.

Line profile estimation

An initialization is advantageous to the main detection algorithm as variational frameworks are known to be robust but require many iterations and the result is sensitive to the starting point. The developed initialization algorithm operates on minimum intensity projected (MIP) images of the stack containing the pipette. Two independent runs are required on different projection images (usually Z and X projections) to get a 3D point estimation.

The initialization algorithm estimates the tip position by analyzing a line profile in the MIP image. First, it is assumed that the orientation of the pipette is known either by setting this value manually or using the value set by the pipette calibration step. Then a line is placed in front of the pipette on the edge of the image and rotated such that it is perpendicular to the orientation of the pipette. Then the line profile is analyzed in those positions where it covers the image. Since the image is often noisy we compute the 90th percentile of the pixel values from the line profile which will be a reference value. If the profile includes at least one value that is relevantly smaller than the reference value it is considered to be caused by the edge of the pipette. The relevantly smaller value is defined as a 40% intensity drop but it can be set as a parameter to the algorithm. If more than one relevant values are present the smallest is chosen. Subsequent to this the position in the image of the chosen relevant small value is computed from the parameters of the line, the algorithm terminates and outputs this value as the position of the pipette tip. If no such value is present in the profile then the line is pushed towards the pipette tip by one pixel distance and the algorithm repeats from analyzing the line's profile. The different steps of the algorithm are presented in Supplementary Fig. 1. The quality measurement of this initialization step is described in Section .

Pipette Hunter 3D

The **notations** used throughout this section are listed below:

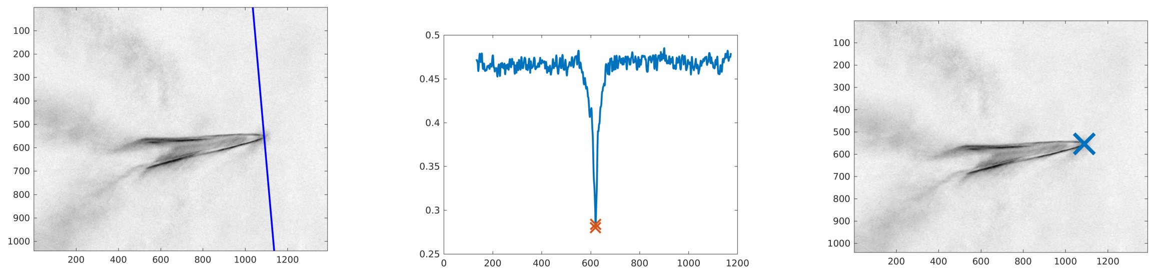

a b c

Supplementary Figure 1: Initialization using line profile estimation. **a:** A line aligned perpendicularly to the orientation of the pipette is pushed against the pipette from the edge of the image. **b:** The line profile of the image where the first relevant intensity drop was found. **c:** The position of the intensity drop in the line profile is converted back to a position in the image.

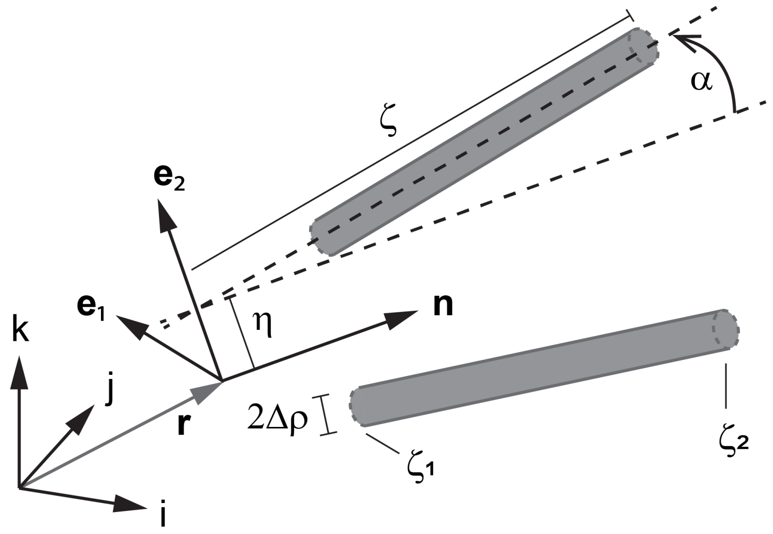

Supplementary Figure 2: The 8 DOF pipette detection mechanism.

- tensors (including vectors) are distinguished from scalars or coordinates using bold letters
- we use different notations for tensors and their representations: representation $[\mathbf{A}]$ or coordinate matrix (w.r.t. some coordinate system) encased in brackets is associated with tensor $\mathbf{A}$
- for dot (scalar) and cross products between vectors, we use operators "$\cdot$" and "$\times$" respectively
- dot operation is used in a broader "contraction" sense: (single-) dot product reduces the order of the respective tensor product by two; examples a) let $\mathbf{v}$, $\mathbf{w}$ be arbitrary vectors (tensors of 1st order), their tensor (dyadic) product is a second order tensor, then $\mathbf{v} \cdot \mathbf{w}$ is scalar (tensor of 0-th order) b) $\mathbf{w} = \mathbf{A} \cdot \mathbf{v}$ where $\mathbf{A}$ is second order tensor and $\mathbf{v}$ is vector, then $\mathbf{w}$ is also a vector c) $\mathbf{C} = \mathbf{A} \cdot \mathbf{B}$ all tensors of order two; between the representations, these operations are performed using the usual matrix-vector and matrix-matrix multiplications (e.g. $[\mathbf{w}] = [\mathbf{A}]\,[\mathbf{v}]$, $[\mathbf{C}] = [\mathbf{A}]\,[\mathbf{B}]$)

- to denote the gradient of a scalar field $I(\mathbf{r})$ (where $\mathbf{r}$ is a position vector w.r.t. some coordinate system) we use either $\frac{\partial I}{\partial \mathbf{r}}$ or the "right gradient" notation $I\nabla$.

Here, we define the energy and derive its gradient for the two-wand mechanism designed to detect the pipette (see Supplementary Fig.2). The assumptions are as follows:

- original configuration: the moving local frame $(\mathbf{e}_1, \mathbf{e}_2, \mathbf{n})$ with coordinates ξ^1, ξ^2, ξ^3 is aligned with the standard basis $(\mathbf{i}, \mathbf{j}, \mathbf{k})$ with coordinates denoted by x^1, x^2, x^3
- the axes of the wands are situated on the plane spanned by $\mathbf{e}_2, \mathbf{n}$, satisfying the equation $\xi^2 = \pm\eta^1 \pm \xi^3 \tan\alpha^1$
- the symmetrical arrangement of the wands is retained during the iterative approaching of the energy minimum
- ζ is the distance along the wand axes measured from the plane spanned by $\mathbf{e}_1, \mathbf{e}_2$, ρ is the distance to the wand axes and ϕ is the angle around it, measured from $\mathbf{e}_1$ in the plane $\mathbf{e}_1, \mathbf{e}_2$.

The wand positions are completely determined by (together with the symmetry condition above)

- the position vector $\mathbf{r}(x^1, x^2, x^3) = x^1\mathbf{i} + x^2\mathbf{j} + x^3\mathbf{k}$ that designates the origin (or pivot point) of the local frame $(\mathbf{e}_1, \mathbf{e}_2, \mathbf{n})$
- the rotation of the local frame about its pivot point (we parameterize the space of rotations with proper Euler angles $\varphi^1, \varphi^2, \varphi^3$, using extrinsic $z - y - z$ scheme)
- the coordinates η^1, α^1 which are given relative to the moving local frame
- the (fixed parameter) values ζ_1, ζ_2 representing the start and the endpoints of the wands along their axes.

The system has therefore 8 degrees of freedom (DOF) given by the variables having upper indices in the list above: $x^1, x^2, x^3, \varphi^1, \varphi^2, \varphi^3, \eta^1, \alpha^1$.

The local coordinates of a point w.r.t. the local frame $(\mathbf{e}_1, \mathbf{e}_2, \mathbf{n})$ inside the cylinder-shaped wands, using cylindrical coordinates ρ, ϕ, ζ are given by:

$$\begin{aligned} \xi^1(\rho,\phi,\zeta) &= \rho\cos\phi \\ \xi^2(\rho,\phi,\zeta) &= \pm\left(\eta^1 + \zeta\sin\alpha^1\right) + (\rho\sin\phi)\cos\alpha^1 \\ \xi^3(\rho,\phi,\zeta) &= \zeta\cos\alpha^1 \mp (\rho\sin\phi)\sin\alpha^1, \end{aligned} \tag{1}$$

$\rho \in [0, \triangle\rho]$, $\zeta \in [\zeta_1, \zeta_2]$, $\phi \in [0, 2\pi)$, then the internal point of wands $\mathbf{P}\left(x^i, \varphi^k, \eta^1, \alpha^1\right) = \mathbf{r} + \xi^1\mathbf{e}_1 + \xi^2\mathbf{e}_2 + \xi^3\mathbf{n}$, where $i,\ k = 1, 2, 3$ is identified by the formula:

$$\begin{aligned} \mathbf{P}_\pm = \mathbf{r}\left(x^i\right) & \\ &\pm \eta^1\mathbf{e}_2\left(\varphi^k\right) + \zeta\left(\cos\alpha^1\mathbf{n}\left(\varphi^k\right) \pm \sin\alpha^1\mathbf{e}_2\left(\varphi^k\right)\right) \\ &+ \rho\left[\sin\phi\left(\cos\alpha^1\mathbf{e}_2\left(\varphi^k\right) \mp \sin\alpha^1\mathbf{n}\left(\varphi^k\right)\right) + \cos\phi\mathbf{e}_1\left(\varphi^k\right)\right]. \end{aligned} \tag{2}$$

Note that in the expression (2), the second line identifies the points of the wand axes. Now we are ready to define the energy of the system as the integral of the voxel-intensity over the volume occupied with the wands:

$$E\left(x^i, \varphi^k, \eta^1, \alpha^1\right) \doteq \int_V \left[I\left(\mathbf{P}_+\right) + I\left(\mathbf{P}_+\right)\right] dV$$
$$= \int_{\zeta_1}^{\zeta_2} \int_0^{2\pi} \int_0^{\triangle\rho} \left[I\left(\mathbf{P}_+\right) + I\left(\mathbf{P}_+\right)\right] \rho d\rho d\phi d\zeta. \quad (3)$$

The local coordinates as the functions of the variables of the integration ρ, ϕ, ζ are given by (1). Energy (3) has its minimal value wherever its derivatives w.r.t. the variables defining the system DOF are all zero. The list of the required derivatives is:

$$\begin{aligned}
\frac{\partial E}{\partial \mathbf{r}} &= \int_V \left[I\nabla\left(\mathbf{P}_+\right) + I\nabla\left(\mathbf{P}_-\right)\right] dV \\
\frac{\partial E}{\partial \varphi^k} &= \int_V \left[I\nabla\left(\mathbf{P}_+\right) \cdot \frac{\partial \mathbf{P}_+}{\partial \varphi^k} + I\nabla\left(\mathbf{P}_-\right) \cdot \frac{\partial \mathbf{P}_-}{\partial \varphi^k}\right] dV \\
\frac{\partial E}{\partial \eta^1} &= \int_V \left[I\nabla\left(\mathbf{P}_+\right) \cdot \frac{\partial \mathbf{P}_+}{\partial \eta^1} + I\nabla\left(\mathbf{P}_-\right) \cdot \frac{\partial \mathbf{P}_-}{\partial \eta^1}\right] dV \\
\frac{\partial E}{\partial \alpha^1} &= \int_V \left[I\nabla\left(\mathbf{P}_+\right) \cdot \frac{\partial \mathbf{P}_+}{\partial \alpha^1} + I\nabla\left(\mathbf{P}_-\right) \cdot \frac{\partial \mathbf{P}_-}{\partial \alpha^1}\right] dV\,.
\end{aligned} \quad (4)$$

Below we examine the integrands of (4) line by line.

1st line: The components of $\frac{\partial E}{\partial \mathbf{r}}$ are the derivatives w.r.t. the coordinates of the origin of the local frame in the world coordinate system: $\frac{\partial E}{\partial x^i} = \frac{\partial E}{\partial \mathbf{r}} \cdot \mathbf{l}$, $\mathbf{l} \in \{\mathbf{i}, \mathbf{j}, \mathbf{k}\}$. $I\nabla$ is the 'right' gradient (represented by row vector) of the image function $I\left(x^1, x^2, x^3\right)$, "$\cdot$" stands for the dot (scalar) product of vectors.

2nd line: Since the local coordinates (1) are known for all values of integration variables $\rho \in [0, \triangle\rho]$, $\zeta \in [\zeta_1, \zeta_2]$, $\phi \in [0, 2\pi)$, we wish to express the quantities that occur in the list of derivatives (4) with these local coordinates. According to (2), only the derivatives of the rotated local frame basis vectors w.r.t. the Euler angles are required. The columns of the representing matrix of the rotation $\mathbf{R} = \mathbf{R}_{\varphi^1} \cdot \mathbf{R}_{\varphi^2} \cdot \mathbf{R}_{\varphi^3}$ is calculated as the multiplication of the matrices of the elementary rotations (applied from the left to column vectors)

$$[\mathbf{R}] = \begin{bmatrix} c_1c_2c_3 - s_1s_3 & -c_3s_1 - c_1c_2s_3 & c_1s_2 \\ c_1s_3 + c_2c_3s_1 & c_1c_3 - c_2s_1s_3 & s_1s_2 \\ -c_3s_2 & s_2s_3 & c_2 \end{bmatrix} = \begin{bmatrix} \mathbf{e}_1 & \mathbf{e}_2 & \mathbf{n} \end{bmatrix}, \quad (5)$$

its columns contain the coordinates of the basis vectors of the local system $(\mathbf{e}_1, \mathbf{e}_2, \mathbf{n})$ expressed in the standard basis (the first two angles φ^1 and φ^2 designate the azimuthal and polar coordinates of $\mathbf{n}$ respectively whilst φ^3 represents the spin of the plane spanned by $\mathbf{e}_1, \mathbf{e}_2$). Here we use the usual abbreviations $s_i \equiv \sin\varphi^i$, $c_k \equiv \cos\varphi^k$, i, $k = 1, 2, 3$ for concise writing. The derivatives are determined by direct calculation, the results can be

expressed by the angles and the local basis vectors as:

$$
\begin{aligned}
\frac{\partial \mathbf{e}_1}{\partial \varphi^1} &= c_2\mathbf{e}_2 - s_2 s_3 \mathbf{n} & \frac{\partial \mathbf{e}_1}{\partial \varphi^2} &= -c_3\mathbf{n} & \frac{\partial \mathbf{e}_1}{\partial \varphi^3} &= \mathbf{e}_2 \\
\frac{\partial \mathbf{e}_2}{\partial \varphi^1} &= -c_2\mathbf{e}_1 - s_2 c_3 \mathbf{n} & \frac{\partial \mathbf{e}_2}{\partial \varphi^2} &= s_3\mathbf{n} & \frac{\partial \mathbf{e}_2}{\partial \varphi^3} &= -\mathbf{e}_1 \\
\frac{\partial \mathbf{n}}{\partial \varphi^1} &= s_2\left(s_3\mathbf{e}_1 + c_3\mathbf{e}_2\right) & \frac{\partial \mathbf{n}}{\partial \varphi^2} &= c_3\mathbf{e}_1 - s_3\mathbf{e}_2 & \frac{\partial \mathbf{n}}{\partial \varphi^3} &= \mathbf{0}.
\end{aligned}
\tag{6}
$$

Note that these derivatives are of constant length vectors - the unit-length local basis vectors - hence lying in their perpendicular plane (e.g. $\frac{\partial \mathbf{e}_1}{\partial \varphi^k} \in \mathit{span}\{\mathbf{e}_2,\, \mathbf{n}\}$). From (6), for the point $\mathbf{P} = \mathbf{r} + \xi^1\mathbf{e}_1 + \xi^2\mathbf{e}_2 + \xi^3\mathbf{n}$, $\mathbf{P} \in \{\mathbf{P}_+, \mathbf{P}_-\}$ the partial derivative expressions: $I\nabla(\mathbf{P}) \cdot \frac{\partial \mathbf{P}}{\partial \varphi^k}$ (the integrands) w.r.t. the Euler angles $\varphi^1, \varphi^2, \varphi^3$ are calculated such that

$$
\begin{aligned}
I\nabla(\mathbf{P}) \cdot \frac{\partial \mathbf{P}}{\partial \varphi^1} &= \left(\xi^3 s_2 s_3 - \xi^2 c_2\right)(I\nabla \cdot \mathbf{e}_1) \\
&\quad + \left(\xi^3 s_2 c_3 + \xi^1 c_2\right)(I\nabla \cdot \mathbf{e}_2) - \left(\xi^1 s_2 s_3 + \xi^2 s_2 c_3\right)(I\nabla \cdot \mathbf{n}) \\
I\nabla(\mathbf{P}) \cdot \frac{\partial \mathbf{P}}{\partial \varphi^2} &= \xi^3 c_3 (I\nabla \cdot \mathbf{e}_1) - \xi^3 s_3 (I\nabla \cdot \mathbf{e}_2) + \left(\xi^2 s_3 - \xi^1 c_3\right)(I\nabla \cdot \mathbf{n}) \\
I\nabla(\mathbf{P}) \cdot \frac{\partial \mathbf{P}}{\partial \varphi^3} &= -\xi^2 (I\nabla \cdot \mathbf{e}_1) + \xi^1 (I\nabla \cdot \mathbf{e}_2),
\end{aligned}
\tag{7}
$$

where the image gradient is decomposed in the local system as

$$
I\nabla = (I\nabla \cdot \mathbf{e}_1)\,\mathbf{e}_1 + (I\nabla \cdot \mathbf{e}_2)\,\mathbf{e}_2 + (I\nabla \cdot \mathbf{n})\,\mathbf{n} \tag{8}
$$

and the wand points are identified with equations (1). To gain deeper insight it is worth introducing the pseudovector $\mathbf{M} \doteq (\mathbf{P} - \mathbf{r}) \times I\nabla$ - the "torque" - w.r.t. the pivot point of the Euler angles, that is the origin of the local basis $(\mathbf{e}_1, \mathbf{e}_2, \mathbf{n})$:

$$
\begin{aligned}
\mathbf{M} &= \left(\xi^1\mathbf{e}_1 + \xi^2\mathbf{e}_2 + \xi^3\mathbf{n}\right) \times \left[(I\nabla \cdot \mathbf{e}_1)\,\mathbf{e}_1 + (I\nabla \cdot \mathbf{e}_2)\,\mathbf{e}_2 + (I\nabla \cdot \mathbf{n})\,\mathbf{n}\right] \\
&= \left[\xi^2 (I\nabla \cdot \mathbf{n}) - \xi^3 (I\nabla \cdot \mathbf{e}_2)\right]\mathbf{e}_1 \\
&\quad + \left[\xi^3 (I\nabla \cdot \mathbf{e}_1) - \xi^1 (I\nabla \cdot \mathbf{n})\right]\mathbf{e}_2 \\
&\quad + \left[\xi^1 (I\nabla \cdot \mathbf{e}_2) - \xi^2 (I\nabla \cdot \mathbf{e}_1)\right]\mathbf{n}\,.
\end{aligned}
\tag{9}
$$

With this pseudovector, the constituents of the energy derivatives w.r.t. the Euler angles (7) can be expressed as:

$$
\begin{aligned}
I\nabla(\mathbf{P}) \cdot \frac{\partial \mathbf{P}}{\partial \varphi^1} &= \mathbf{M} \cdot \left[s_2\left(-c_3\mathbf{e}_1 + s_3\mathbf{e}_2\right) + c_2\mathbf{n}\right] = \mathbf{M} \cdot \mathbf{k} \\
I\nabla(\mathbf{P}) \cdot \frac{\partial \mathbf{P}}{\partial \varphi^2} &= \mathbf{M} \cdot \left(s_3\mathbf{e}_1 + c_3\mathbf{e}_2\right) = \mathbf{M} \cdot \left(\mathbf{R}_{\varphi^1} \cdot \mathbf{j}\right) \\
I\nabla(\mathbf{P}) \cdot \frac{\partial \mathbf{P}}{\partial \varphi^3} &= \mathbf{M} \cdot \mathbf{n} = \mathbf{M} \cdot \left(\mathbf{R}_{\varphi^1} \cdot \mathbf{R}_{\varphi^2} \cdot \mathbf{k}\right)\,.
\end{aligned}
\tag{10}
$$

Formulae (10) are easier to understand if the rotations are interpreted intrinsically. The rate of change of the Euler angles are proportional to the decomposition of torque $\mathbf{M}$ w.r.t. the orientation of the instantaneous reference frame moving/rotating together

with the mechanism: a) first the whole mechanism - with its reference frame attached - spins around axis $\mathbf{k}$ then b) around the rotated axis $\mathbf{R}_{\varphi^1} \cdot \mathbf{j}$ and finally c) around the twice-rotated axis $\mathbf{R}_{\varphi^1} \cdot \mathbf{R}_{\varphi^2} \cdot \mathbf{k} = \mathbf{n}$.

For the calculation of $\frac{\partial E}{\partial \varphi^k}$, $k = 1, 2, 3$ either equations (7) or the equations (10) with torque values (9) integrated over the coordinates ξ^1, ξ^2, ξ^3 identified as wand point coordinates by (1) can be used.

3rd line: From (2) $\frac{\partial \mathbf{P}_\pm}{\partial \eta^1} = \pm \mathbf{e}_2$ hence we have:

$$I\nabla\left(\mathbf{P}_\pm\right) \cdot \frac{\partial \mathbf{P}_\pm}{\partial \eta^1} = \pm I\nabla \cdot \mathbf{e}_2 \,. \tag{11}$$

4th line: With direct calculation, the integrands are given by:

$$\begin{aligned} I\nabla\left(\mathbf{P}_\pm\right) \cdot \frac{\partial \mathbf{P}_\pm}{\partial \alpha^1} &= \left(\pm\zeta \cos\alpha^1 - \rho \sin\alpha^1 \sin\phi\right) \left[I\nabla\left(\mathbf{P}_\pm\right) \cdot \mathbf{e}_2\right] \\ &\quad - \left(\zeta \sin\alpha^1 \pm \rho \cos\alpha^1 \sin\phi\right) \left[I\nabla\left(\mathbf{P}_\pm\right) \cdot \mathbf{n}\right] \,. \end{aligned} \tag{12}$$

Result (12) can also be viewed as the action of the pseudovectors

$$\mathbf{m}_\pm = \left[\left(\xi^2 \mp \eta^1\right) \mathbf{e}_2 + \xi^3 \mathbf{n}\right] \times I\nabla\left(\mathbf{P}_\pm\right) \tag{13}$$

w.r.t. their momentary rotational axes: $-\mathbf{e}_1$ at local coordinates $(0, \eta^1, \xi^3)$ and $\mathbf{e}_1$ at local coordinates $(0, -\eta^1, \xi^3)$ respectively (in the former case, a minus sign is required to satisfy the right-hand rule). Substituting (1) to torque expressions (13) we have

$$\begin{aligned} -\mathbf{m}_+ \cdot \mathbf{e}_1 &= \left[I\nabla\left(\mathbf{P}_+\right) \cdot \mathbf{e}_2\right] \xi^3 - \left[I\nabla\left(\mathbf{P}_+\right) \cdot \mathbf{n}\right] \left(\xi^2 - \eta^1\right) \\ &= \left(\zeta \cos\alpha^1 - \rho \sin\alpha^1 \sin\phi\right) \left(I\nabla \cdot \mathbf{e}_2\right) \\ &\quad - \left(\zeta \sin\alpha^1 + \rho \cos\alpha^1 \sin\phi\right) \left(I\nabla \cdot \mathbf{n}\right) \\ \mathbf{m}_- \cdot \mathbf{e}_1 &= -\left[I\nabla\left(\mathbf{P}_+\right) \cdot \mathbf{e}_2\right] \xi^3 + \left[I\nabla\left(\mathbf{P}_+\right) \cdot \mathbf{n}\right] \left(\xi^2 - \eta^1\right) \\ &= -\left(\zeta \cos\alpha^1 + \rho \sin\alpha^1 \sin\phi\right) \left(I\nabla \cdot \mathbf{e}_2\right) \\ &\quad + \left(-\zeta \sin\alpha^1 + \rho \cos\alpha^1 \sin\phi\right) \left(I\nabla \cdot \mathbf{n}\right) \end{aligned} \tag{14}$$

which is equivalent to (12).

Comparison

The performance of the algorithms was evaluated on 5 acquired image stacks of different pipettes. The pipettes were lowered around the working distance to have similar light conditions to those during the experiments with samples. The pipette tip is moved over at least half of the image and it is assured that the stack always contains it. The voxel position of the tip was manually selected in the stacks. Then the initialization algorithm was run on the stacks and its results were the starting point of PH3D. The results of both the initialization algorithm and PH3D were saved and compared to the manually picked positions. The mean difference between the line profile estimation and the ground truth data is 14.64 ± 4.23 voxels, while for PH3D the error is 8.63 ± 4.80 voxels. The pixel size is 115 nm and the distance between the slices in the stack was 1 μm, thus the error expressed in micrometers is 0.99 ± 0.55 μm. This error value is fairly low to reliably hit a cell if the pipette is oriented to its center, since the diameter of cells is usually in the range of 5-20 μm. Furthermore, this error value is smaller than the reported 3.53 ± 2.47 μm or 32.97 ± 23.10 pixels in the case of the original 2D model.

Supplementary Note 2:
Deep Learning Model Comparison for Cell Detection

DetectNet

The first model in the comparison was DetectNet [2]. DetectNet can be split into training and validation sections. The training part (Supplementary Fig. 3) contains the data augmentation, the fully connected network (GoogLeNet [3]), and the loss functions, while the bounding box clustering and the mean average precision (mAP) statistics are the elements of the validation part. The training was performed in NVIDIA's Deep Learning GPU Training System (DIGITS [4]) architecture, which is an extension of *caffe* [5]. Adaptive Moment Estimation (ADAM) [6] was used as the solver for the training process. The number of epochs was 500, while the learning rate was 1e-5 with a fixed learning rate policy. The pre-trained weights of the ImageNet dataset were used for the initialization of GoogLeNet to speed up the training process. The input image size was downscaled to 704×512, and the mini-batch size was set to 10. The training was performed using an NVIDIA Titan Xp graphics card.

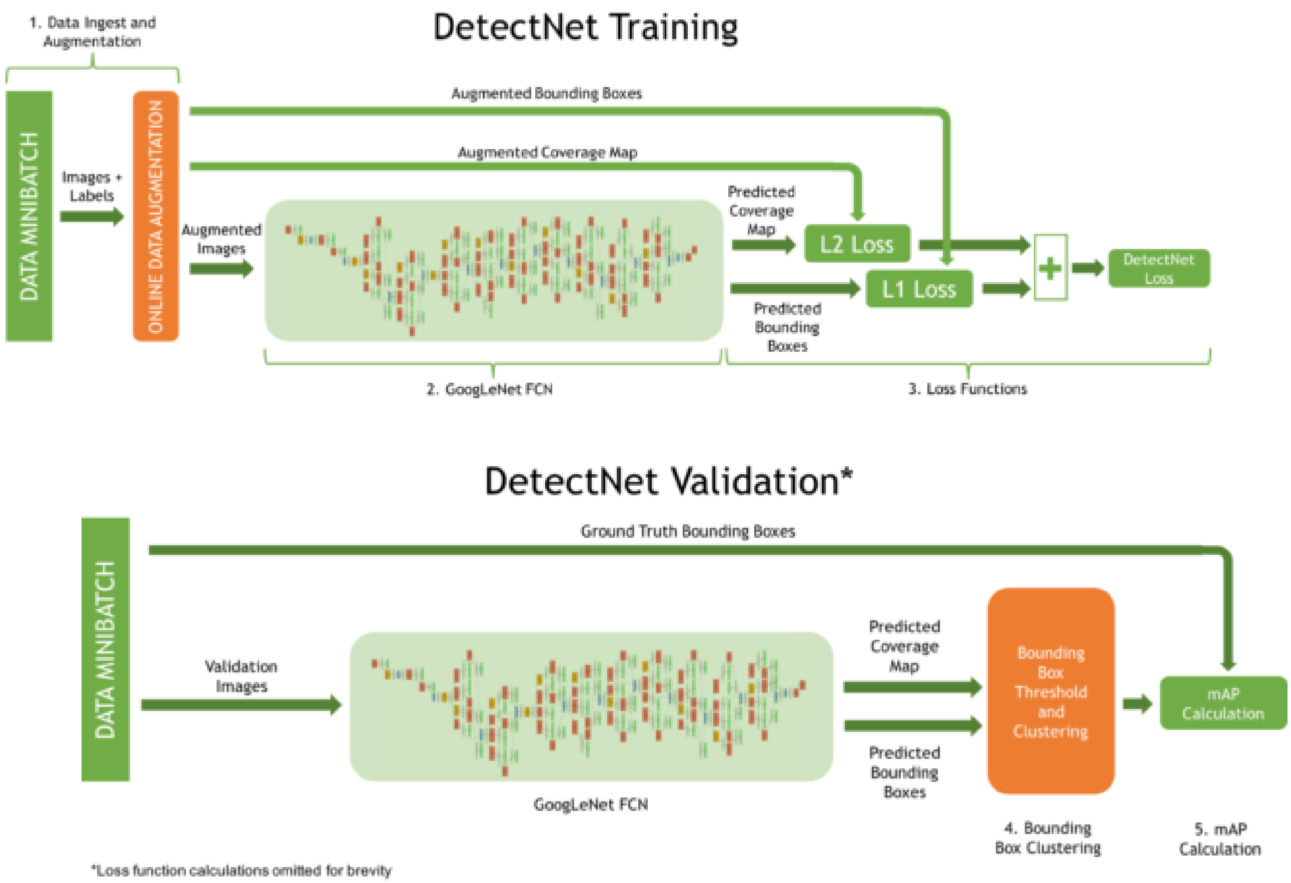

Supplementary Figure 3: DetectNet structure for training and validation. (Source: https://developer.nvidia.com/blog/)

Faster R-CNN

Faster R-CNN (FRCNN) [7] (Supplementary Fig. 4) with ResNet50 [8] backbone (Supplementary Fig. 8) pretrained on ImageNet was trained in Matlab R2019b. The Stochastic Gradient Descent with Momentum (SGDM) [9] was used as the optimizer with cross-entropy loss function. The number of epochs was 6. The initial learning rate was 1e-3, which was dropped every 15 epochs by a factor of 0.2. The training method was set to 'end-to-end', that simultaneously trains the region proposal and region classification subnetworks. The input image size was downscaled to 696×520, and the mini-batch size was limited to 1 by the framework. Box pyramid scale value of 2 was set, with 0-0.4 positive and 0.6-1 negative overlapping ranges. The number of box pyramidal levels were determined automatically by the framework. The training was performed using NVIDIA GeForce RTX 2080 Ti with 11 GB memory.

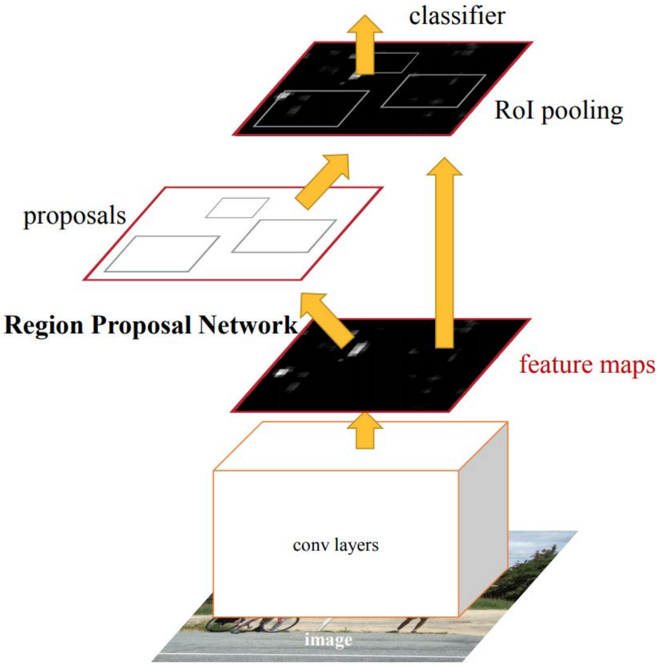

Supplementary Figure 4: Faster RCNN architecture. (Source: [7].)

Darknet with YOLOv3-SPP backbone

With the Python-based PyTorch [10] package we trained two separate Darknet [11] models with different backbones. The first model had YOLOv3-SPP [12] backbone, that we used in the same setup as it was described in the original paper. To evaluate the feature extraction in YOLOv3, a hybrid approach between YOLOv2 and a residual network has been created. The new network uses 3×3 and 1×1 convolutional layers combined with shortcut connections. It is called Darknet-53 (Supplementary Fig. 5), because of the 53 convolutional layers it has in total.

During training, the sum of squared error losses are used. As for the classification, independent logistic classifiers are used to classify the containing of each bounding box, and logistic regression is used to predict an objectness score. Boxes are predicted at 3 different

scales. Similarly to FPN [13] the features are extracted from them. The last convolutional layer predicts a 3D tensor encoding bounding boxes, objectness, and class prediction. In our experiments, we predict 3 boxes at each scale, so as the result we get N×N×[3*(4+1)] for the 4 bounding box offsets and 1 objectness prediction. In this case, we applied ADAM as the optimizer, with 1e-3 learning rate. We used step decay as a learning rate scheduler, where the drop was 5e-4 after every 30 epochs. The total number of epochs we used was 100. The size of the input images was converted to 1024×1024. We set the IOU threshold between the predicted bounding boxes and ground truth data to 0.4. Random affine transformations were added to the training data. The training was performed using an NVIDIA Titan Xp card.

	Type	Filters	Size	Output
	Convolutional	32	3 × 3	256 × 256
	Convolutional	64	3 × 3 / 2	128 × 128
1×	Convolutional	32	1 × 1	
	Convolutional	64	3 × 3	
	Residual			128 × 128
	Convolutional	128	3 × 3 / 2	64 × 64
2×	Convolutional	64	1 × 1	
	Convolutional	128	3 × 3	
	Residual			64 × 64
	Convolutional	256	3 × 3 / 2	32 × 32
8×	Convolutional	128	1 × 1	
	Convolutional	256	3 × 3	
	Residual			32 × 32
	Convolutional	512	3 × 3 / 2	16 × 16
8×	Convolutional	256	1 × 1	
	Convolutional	512	3 × 3	
	Residual			16 × 16
	Convolutional	1024	3 × 3 / 2	8 × 8
4×	Convolutional	512	1 × 1	
	Convolutional	1024	3 × 3	
	Residual			8 × 8
	Avgpool		Global	
	Connected		1000	
	Softmax			

Supplementary Figure 5: Darknet-53/YOLOv3 architecture. (Source: [12].)

Darknet with custom ResNeXt-50 backbone

The second Darknet model we trained was using a custom ResNeXt-50 (ResNeXt) [14] backbone. In this case, the environment is the same as in the YOLOv3 instance, but the network has a different type and number of layers. Within ResNeXt a modularized design of ResNet was adopted. This network consists of a stack of residual blocks. As in ResNets these blocks have the same topology (Supplementary Fig. 6), and they are subject to two simple rules: when spatial maps of the same size are produced, the blocks will share the same hyper-parameters, and when a spatial map is downsampled by a factor of 2, the width of the blocks is multiplied by a factor of 2. These 2 rules are indicating the only need to design a template module, and all of the modules in a network can be determined accordingly (Supplementary Fig. 8).

It can be shown that the module of Supplementary Fig. 7a is equivalent to Supplementary

Fig. 7b. The same topology is shared among multiple paths. Using the notation of *grouped convolution*, these modules become more succinct. This reformulation can be seen in Supplementary Fig. 7c.

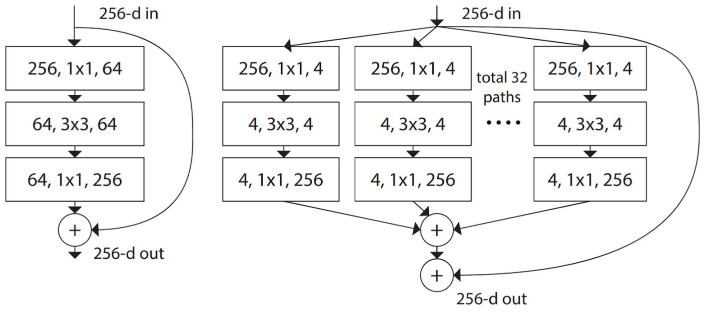

Supplementary Figure 6: residual block of ResNet (left) and residual block of ResNeXt (right). (Source: [14].)

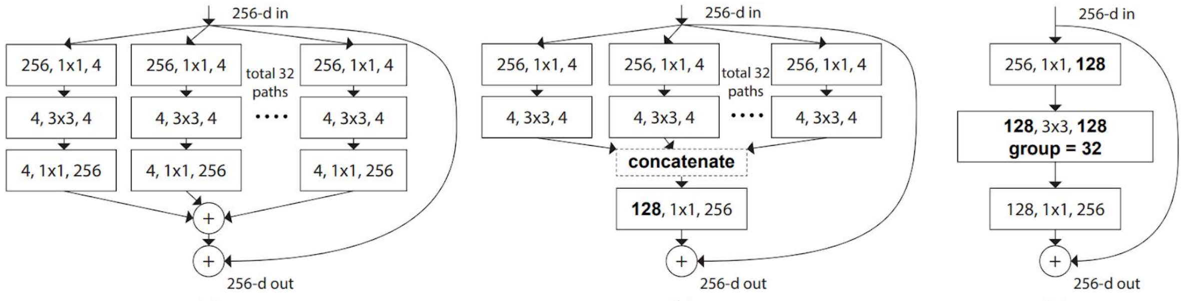

Supplementary Figure 7: **a:** Aggregated residual transformations. **b:** A block equivalent to (a) implemented as early concatenation. **c:** A block equivalent to (a, b), implemented as grouped convolutions. (Source: [14])

Except for the differences in these blocks, we applied the same network as it is set up in ResNet-50 (Supplementary Fig. 6).

The initial learning rate was 5e-4. We applied a step decay learning rate scheduler, where the drop was 1e-4 after every 30 epochs. We used ADAM as the optimizer. The total number of epochs was 100. The size of the input images was converted to 1024×1024. We set the IOU threshold between the predicted bounding boxes and ground truth data to 0.4. Random affine transformations were added to the training data here as well. The training was performed using an NVIDIA Titan Xp card.

stage	output	ResNet-50	**ResNeXt-50 (32×4d)**
conv1	112×112	7×7, 64, stride 2	7×7, 64, stride 2
conv2	56×56	3×3 max pool, stride 2	3×3 max pool, stride 2
		$\begin{bmatrix} 1\times1, 64 \\ 3\times3, 64 \\ 1\times1, 256 \end{bmatrix} \times 3$	$\begin{bmatrix} 1\times1, 128 \\ 3\times3, 128, C{=}32 \\ 1\times1, 256 \end{bmatrix} \times 3$
conv3	28×28	$\begin{bmatrix} 1\times1, 128 \\ 3\times3, 128 \\ 1\times1, 512 \end{bmatrix} \times 4$	$\begin{bmatrix} 1\times1, 256 \\ 3\times3, 256, C{=}32 \\ 1\times1, 512 \end{bmatrix} \times 4$
conv4	14×14	$\begin{bmatrix} 1\times1, 256 \\ 3\times3, 256 \\ 1\times1, 1024 \end{bmatrix} \times 6$	$\begin{bmatrix} 1\times1, 512 \\ 3\times3, 512, C{=}32 \\ 1\times1, 1024 \end{bmatrix} \times 6$
conv5	7×7	$\begin{bmatrix} 1\times1, 512 \\ 3\times3, 512 \\ 1\times1, 2048 \end{bmatrix} \times 3$	$\begin{bmatrix} 1\times1, 1024 \\ 3\times3, 1024, C{=}32 \\ 1\times1, 2048 \end{bmatrix} \times 3$
	1×1	global average pool<br>1000-d fc, softmax	global average pool<br>1000-d fc, softmax
# params.		$\mathbf{25.5}\times10^6$	$\mathbf{25.0}\times10^6$
FLOPs		$\mathbf{4.1}\times10^9$	$\mathbf{4.2}\times10^9$

Supplementary Figure 8: ResNet-50 and ResNeXt-50 architectures.
Inside the brackets are the shape of a residual block, and outside the brackets is the number of stacked blocks on a stage. "*C*=32" suggests grouped convolutions with 32 groups.
(Source: [14])

Evaluation

Even though the detections are performed in 2D images, the results are extended to 3D in the image stacks the same way as in the software as described in the main text. Then the centroids of both the ground truth and detected bounding boxes are calculated and used for matching them. If the distance between a detected and ground truth bounding box is less than or equal to 5 micrometers (43.48 pixels) in the axial plane, and less than or equal to 3 micrometers / 3 slices in the Z axis, then they are matched and counted as a true positive (TP) detection. These distances are determined empirically such that even in the case of small neurons if the detection centroid falls in the soma, it will be matched to a ground truth. Then precision (P), recall (R), and F1-score (F1) were calculated. The models were evaluated on 3 image stacks (305 images in total). The best result was achieved by using FRCNN, while the rest performed very similarly to each other (Supplementary Table 1). Supplementary Figs. 9-12 contain the precision-recall and ROC (receiver operating characteristic) curves of the models. Example images with detection bounding boxes (yellow rectangles) and ground truth data (red boxes) are shown in Supplementary Figs. 14-19.

Model	F1-score	Precision	Recall
DetectNet	56.88%	53.04%	61.33%
**Faster RCNN**	**65.83%**	**60.73%**	**71.88%**
Darknet (custom ResNeXt)	55.70%	60.55%	51.56%
Darknet (YOLOv3-SPP)	57.31%	57.20%	57.42%

Supplementary Table 1: Evaluation results of the models.

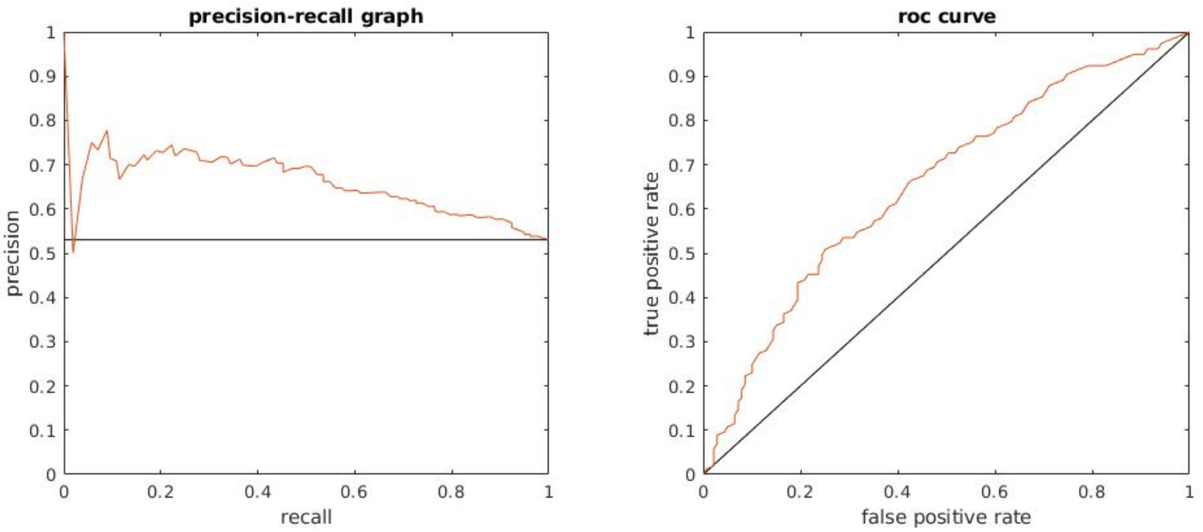

Supplementary Figure 9: precision-recall and ROC curve of the DetectNet model.

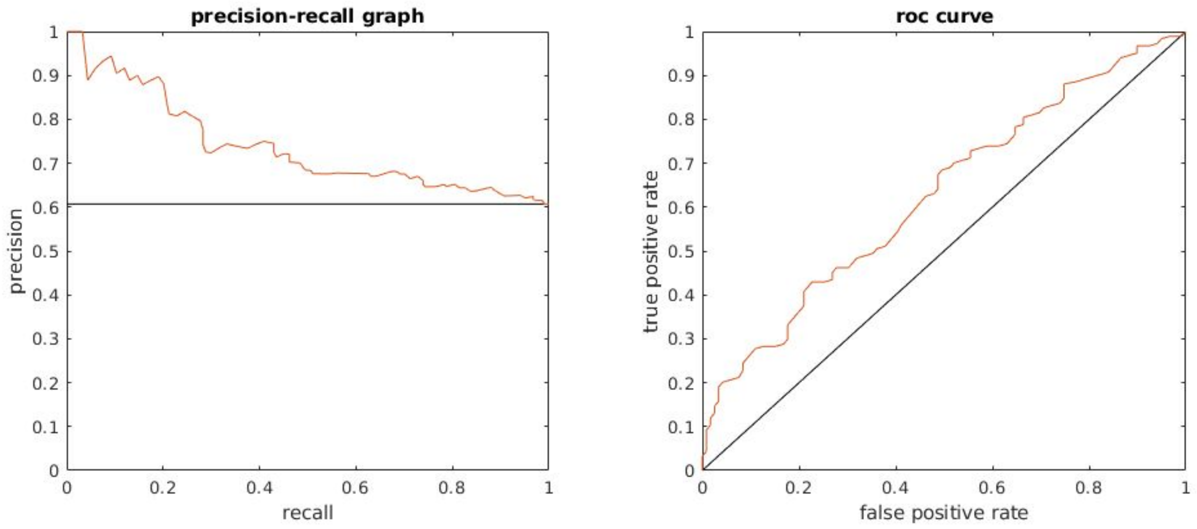

Supplementary Figure 10: precision-recall and ROC curve of the FRCNN model.

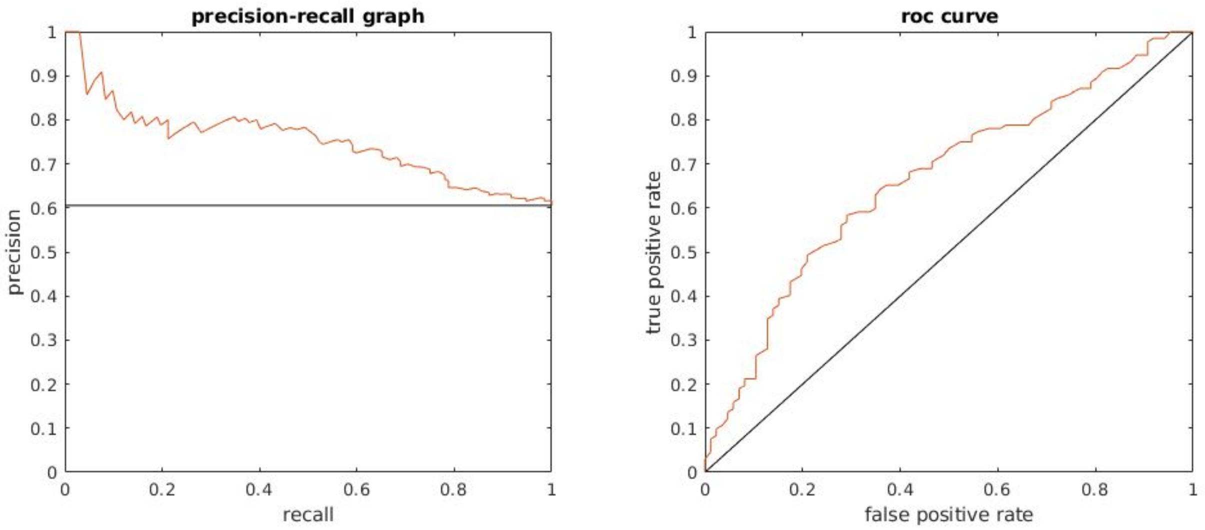

Supplementary Figure 11: precision-recall and ROC curve of the Darknet-ResNeXt model.

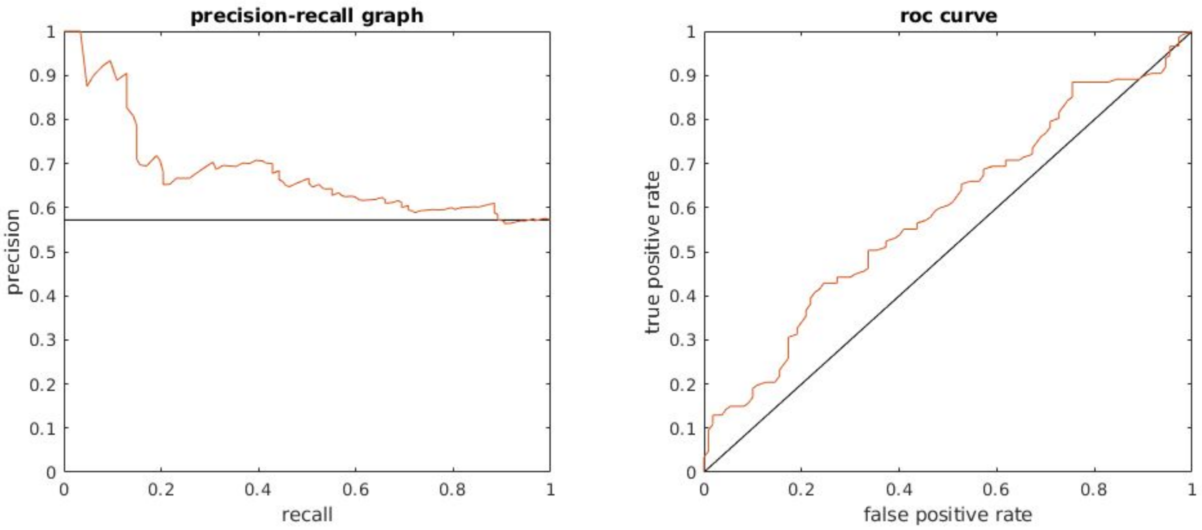

Supplementary Figure 12: precision-recall and ROC curve of the YOLO model.

Results obtained using FRCNN

In the comparison described above the FRCNN architecture provided the best results. To make sure that the model is generalizable we have performed further tests on a subset of images. This representative subset consisted of 35 image stacks of human samples that were imaged in the later stages.

First, we tested how the number of epochs affect the quality. We have run the method for 6 epochs with 0.2 decay every 2 epochs, then 20 epochs with 0.2 decay in every 7 epochs, and

finally 100 epochs with 0.2 decay every 15 epochs. The quality metrics were F1 = 60.13% (P = 58.10%, R = 67.39%), F1 = 63.68% (P = 61.96%, R = 69.27%), and F1=63.06% (P=67.18%, R=61.56%), respectively. This shows that 20 epochs provide somewhat better results compared to Supplementary Table 1, and are a good balance between training time and quality.

Next, we have performed 5-fold cross-validation with 20 epochs. The cross-validation resulted in mean F1 = 65.33%, P = 58.33%, and R = 76.23%, which values are similar to the corresponding ones in Supplementary Table 1.

Finally, we have compared the FRCNN-ResNet50 model to FRCNN-MobileNetV2 [15]. MobileNetV2 is a smaller network that has a great balance between complexity and accuracy [16]. Here, we used 100 epochs for the training. The result quality metrics are F1=60.93%, P = 55.13%, R = 71.47% (Supplementary Fig. 13) . This shows that smaller backbones can be used for the cell detection problem in case of hardware limitations and that FRCNN architecture is a good choice for the task.

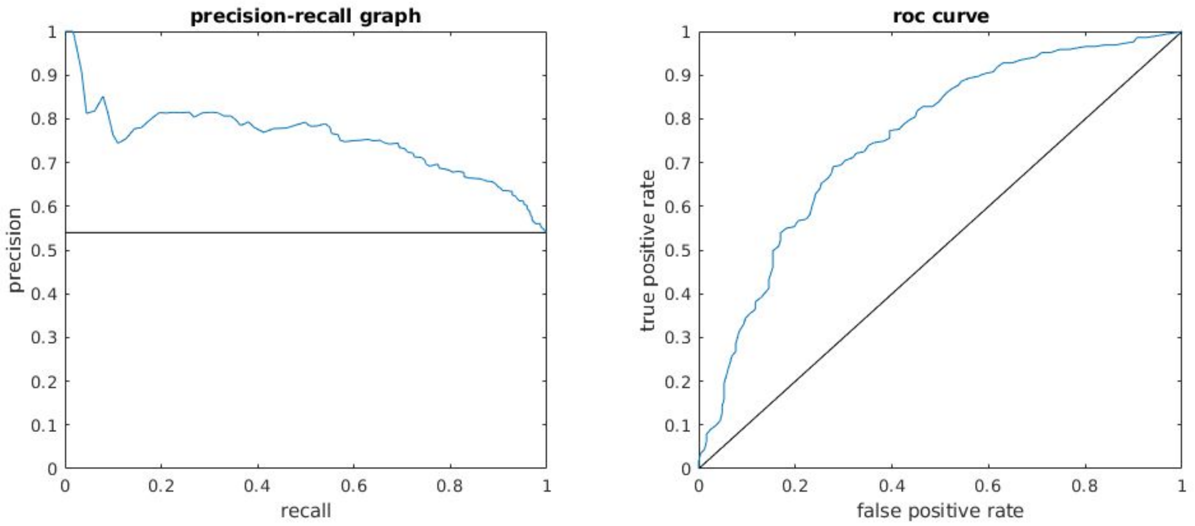

Supplementary Figure 13: precision-recall and ROC curve of the FRCNN-MobileNetV2 model.

Discussion

FasterRCNN highly outperformed other models we tested. In case of all the necessary hardware and software components are available we highly recommend its usage (Matlab 2018 or later, high performance GPU with a minimum of 8GB RAM, we tested the provided model with the following GPU cards: NVidia GeForce 1070, 1080, 1080Ti, 2070, 2080, 2080Ti, Titan Xp). All so often high capacity computers are not available for bench calculations, therefore we also implemented and recommend the use of DetectNet model that is a viable choice when a GPU with more than 4GB RAM is not available.

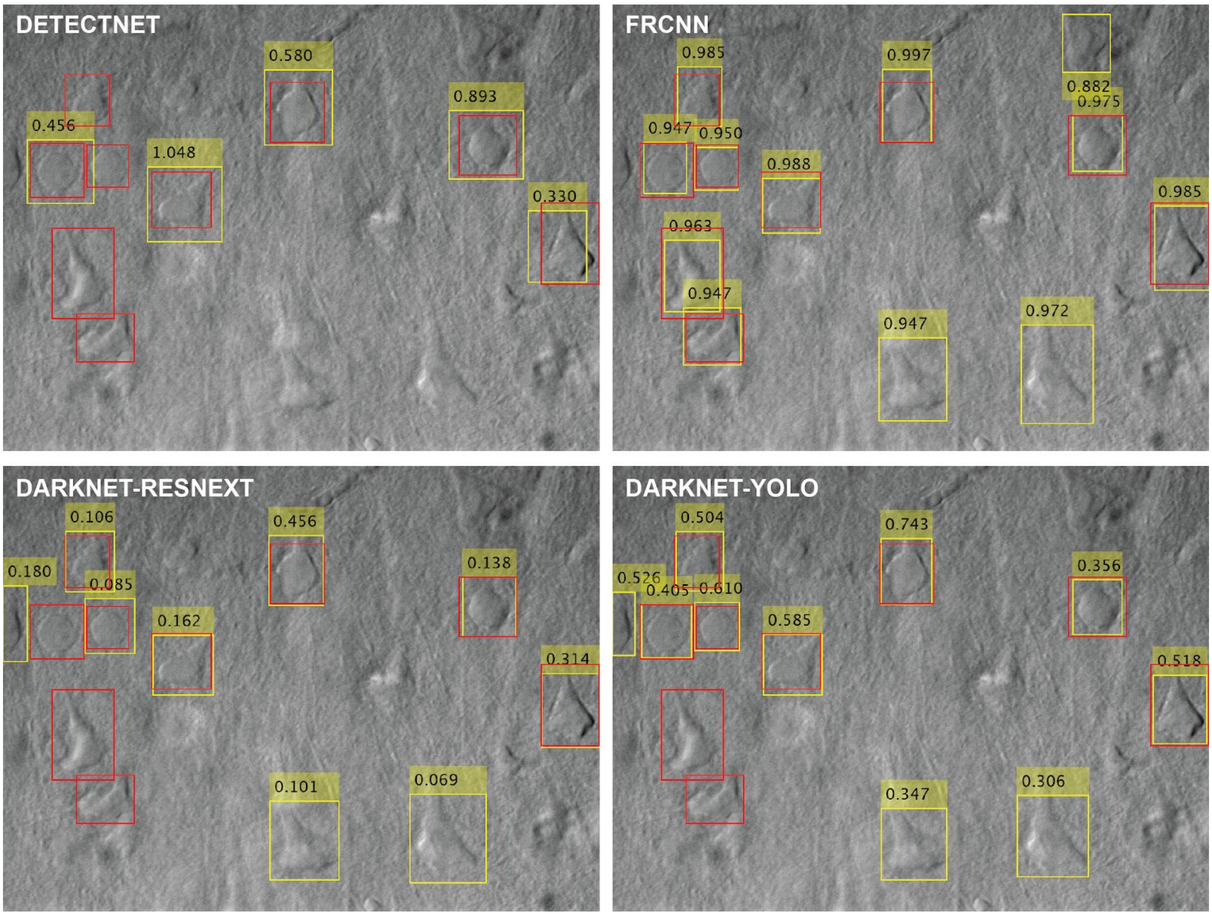

Supplementary Figure 14: Example result images.

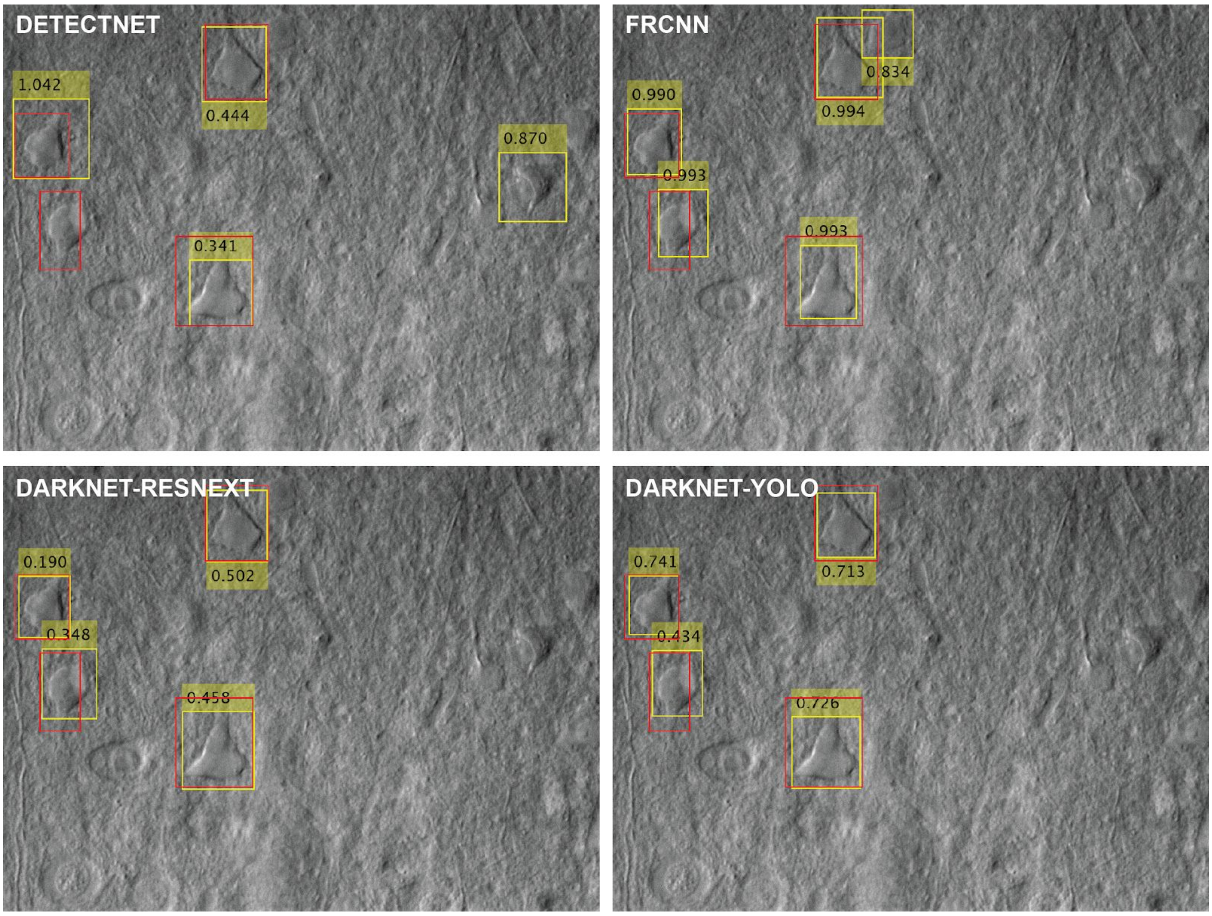

Supplementary Figure 15: Example result images.

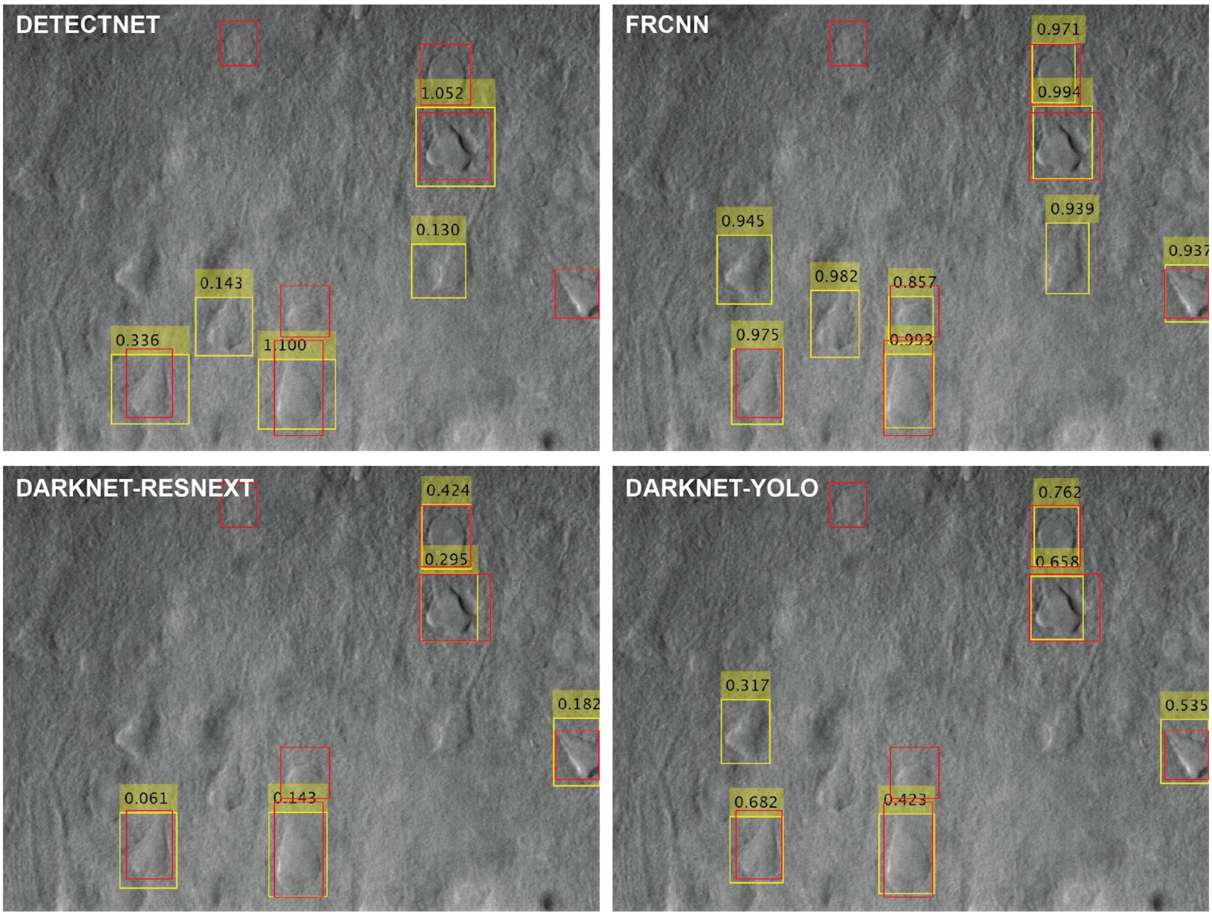

Supplementary Figure 16: Example result images.

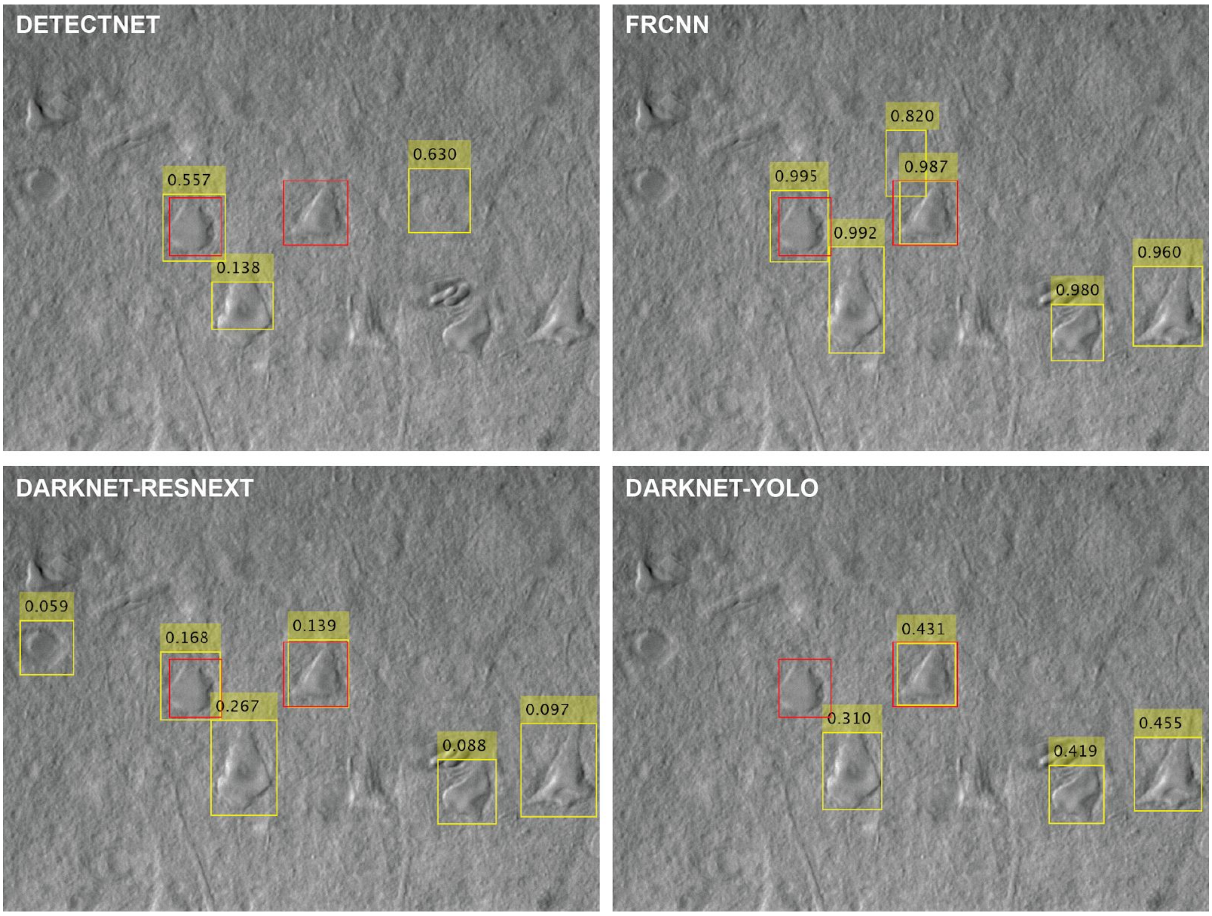

Supplementary Figure 17: Example result images.

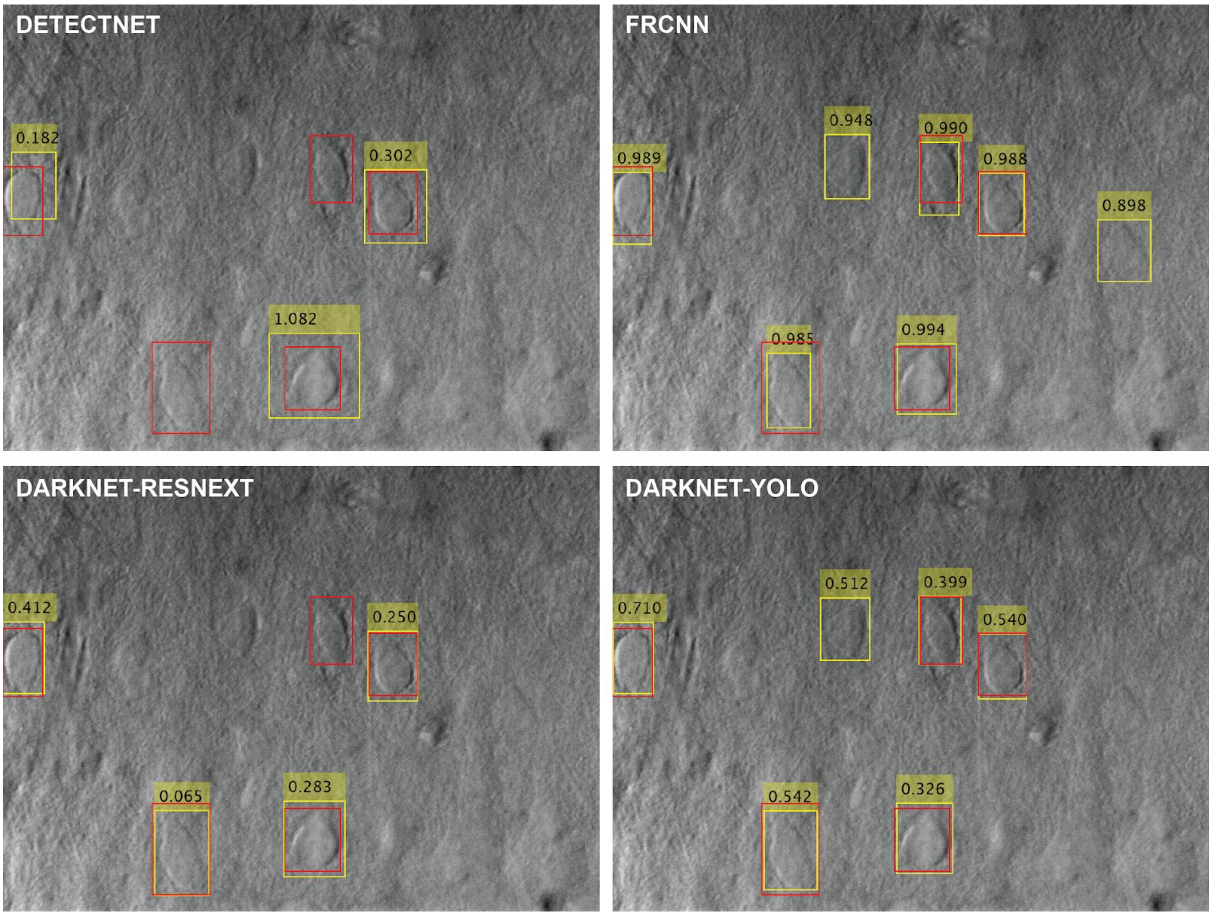

Supplementary Figure 18: Example result images.

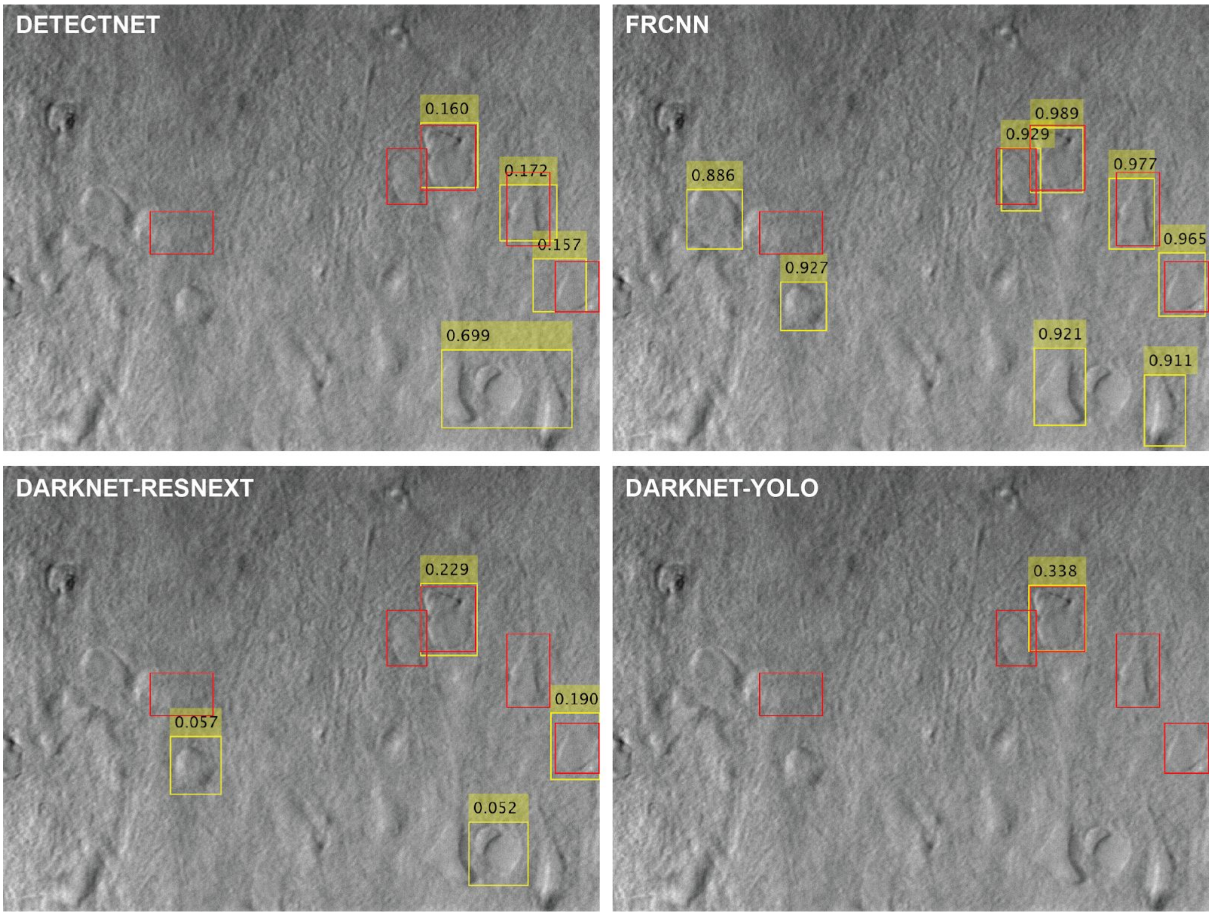

Supplementary Figure 19: Example result images.

Supplementary Note 3: Cell Tracking System

Introduction

The target cell during the patch clamping process can shift as the pipette is being pushed towards it. The shift can occur in any direction in the tissue and based on our experiments it is usually between 3 and 10 µm (6.98 ± 3.91 µm, n=19). Tracking the cell under the microscope is a challenging task because a 3-dimensional tracking is required with a 2-dimensional label-free modality. The developed online tracking system has two parts. One part performs lateral tracking in the XY plane while the other part tracks the cell in the Z dimension. Both parts require a template image of the target cell which is acquired before starting the patch clamp process and when the cell is in focus in the image. The lateral tracking is always performed in the image of the latest focus level. The Z tracking algorithm operates on a small image stack. Acquiring an image stack is time-consuming as the objective has to be moved physically to different focus levels. Therefore, when Z tracking is being performed, the pipette movement is paused to ensure that the cell is not pushed meanwhile.

Lateral cell tracking

The lateral tracker is the Kanade-Lucas-Tomasi (KLT) feature tracker algorithm. Feature points are detected by the minimum eigenvalue algorithm in a window in the template image [17,18]. The algorithm is computationally inexpensive and it is performed often (usually per second) except when Z tracking is in progress. Supplementary Movie 4 shows the performance of the lateral tracker. The white '+' markers at the beginning of the examples in the video show the detected feature points on which the tracker is based. Our experimental verification shows that in 95% of the cases the algorithm was able to track single cells for more than 100 frames, even in case of extreme motion (>3 µm/frame).

The default window size for the tracker was 241x241 pixels which is enough to cover almost every cell (except rarely large human cells). The feature points are detected using a 5x5 filter. If the number of valid feature points significantly drops, for example, due to a sudden movement of the cell in the Z direction, the points are reinitialized. A significant drop of feature points is detected when the number of valid points is less than 10% of their original number or less than or equal to 10.

Tracking in Z dimension

Tracking in the Z dimension is based on focus detection algorithms. As a major principle, we assume that an object which is in focus has sharp edges, that cause large differences in pixel intensities. In the beginning, a small image stack is acquired around the last known position of the target cell. The template image is compared to every slice of the stack by calculating the absolute value of the difference of the standard deviations of the images in a small window. The position of the window is determined by the lateral tracker. The value for the middle slice, which corresponds to the last known focus position, is compensated for noise by a multiplier (0.95 in our case). Then the index of the minimum value determines the direction of the shift of the cell. If the lowest value is of the middle slice, the cell has not moved. If the cell has moved below its previous position then the minimum value will be that of the elements below the middle slice. Similarly, we can determine if the cell has moved up.

Although this approach does not specify the displacement value only the direction of it, the tracking is reliable because the cells do not move rapidly. The stack size used for the tracking, based on empirical tests, consists of 7 slices by default, thus the middle element is the 4th.

Supplementary Fig. 20 shows examples of the computed values and the related images based on which the decisions are made. The data shown are calculated from bigger image stacks, consisting of 60-100 slices. The cells shown were selected from the results of the cell detection system. This allowed showing more values than just 7 for demonstration in the figure.

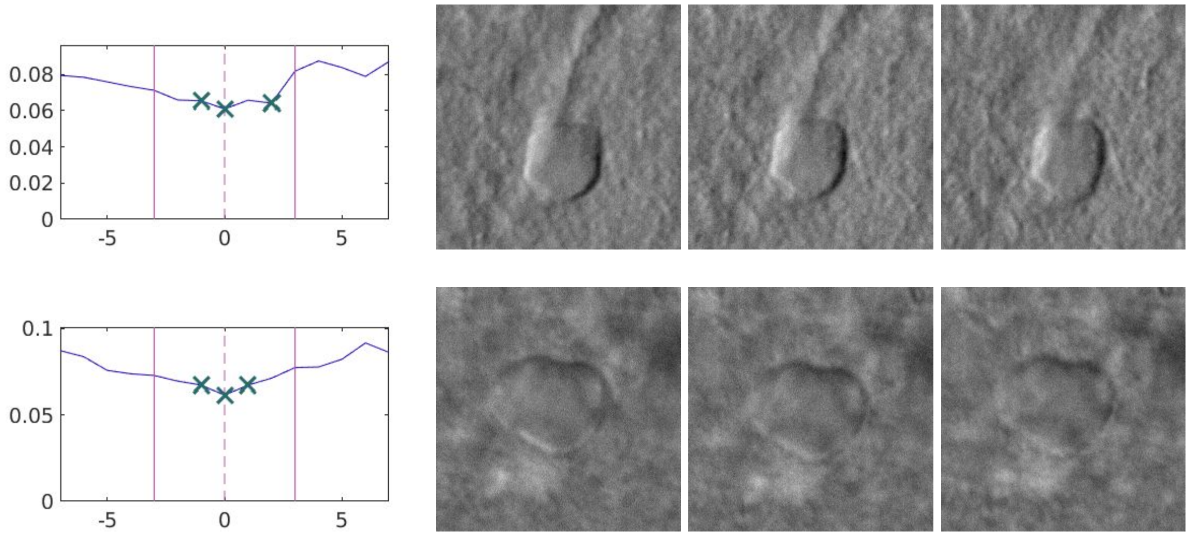

Supplementary Figure 20: Examples of the Z tracking system. The plots in the first column show the calculated values used for determining the direction of the shift. The X-axis shows the index difference from the middle slice. The corresponding image regions of the markers in the plots are shown in the next three columns. The final decision whether the cell has shifted is based on these values and images. The lines at ±3 in the X-axis indicate the region which is used in the calculations, while the values outside this interval are for demonstration purposes.

We have performed a control test to show how the algorithm performs when it is guaranteed that the cell does not move. We have acquired 10 image stacks with 7 slices of the same scene where a cell was in focus in the center of the image in the middle slice. The stacks were manually acquired right after each other without any movement in the stage or the pipette. We have repeated the above 10 times, so we have 10 image stack series of 10 different scenes. We have identified 40 different cells in the scenes, not just the ones that were in the center. Afterward, we have tested our algorithm on this data to check how many times it detects that the target cell has not moved (Supplementary Table 2). The middle slice from the first stack was always used as the reference image and the algorithm was run on the following 9 stacks. The algorithm was able to correctly detect that the cell remained in place in most of the cases. As the pipette is not adjusted immediately (only if the distance from the trajectory is at least 5 micrometers), rare errors do not affect negatively the patch clamping process. Furthermore, the rate of false detections of upward and downward movement is very close to each other and in practice, one compensates the other.

	Accuracy (%)	Error: below (%)	Error: above (%)
Avg	89.17	5.56	5.28
Stdev	9.90	7.96	8.34

Supplementary Table 2: Results of the control test.

Supplementary Note 4: Pressure Regulator Setup

We built a custom pneumatic pressure regulator to apply air pressure on the pipette. The pressure system is equipped with a 50 ml tank in which the requested pressure is set before opening a solenoid valve to the pipette. Two analog pressure sensors (Honeywell, NJ, USA) were used in the system for the closed-loop pressure regulation. One is connected to the tank, the other measures the pressure subsequent to the valve that connects the tank and the pipette. The sensors and the valves are connected to a data acquisition device (DAQ, National Instruments USB-6009, Texas, USA) which controls the valves to set the desired pressure. The pneumatic parts are connected by silicone tubes. Supplementary Fig. 21 shows the wiring diagram of the system. The electrophysiological signal is also forwarded to the same data acquisition device for real-time detection of the impedance change of the pipette tip.

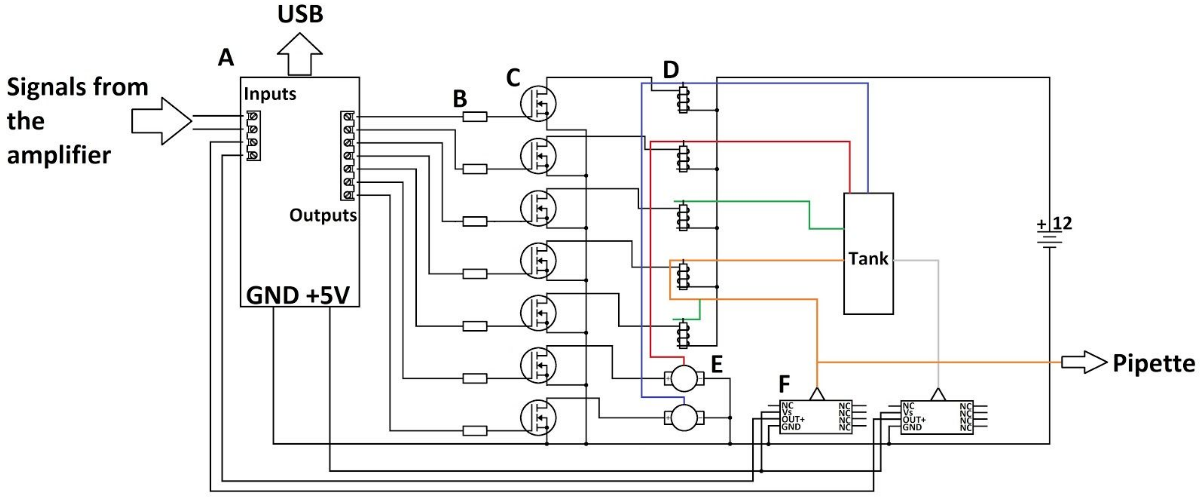

Supplementary Figure 21: Wiring diagram of the custom made pneumatic pressure regulator. A: USB digitizer board (National Instruments, USB-6009), B: 220 Ohm resistors, C: IRFZ44N field effect transistors, D: 12V DC solenoid valves, E: 12V DC pneumatic pumps, F: analog pressure sensors (Honeywell, NJ). Colored lines: pneumatic tubes. red: positive pressure, blue: vacuum, green: atmospheric pressure, orange: desired pressure to the electrode tip, grey: tank pressure. The valves were controlled with TTL signals via the USB board digital outputs. The output voltages of the pressure sensors were measured with the analog input channels of the USB digitizer.

The controller of the pressure system is included in the software. The accuracy of the regulator which is 10 mBar by default can be set from the software. Note that the time it takes to set the pressure can increase if the accuracy is set too high compared to the volume of the installed tank and the silicon tubes. The authors help in providing a kit or an assembled version of the controller on request.

Supplementary Note 5:
Electrophysiology Stimuli for DIGAP

Stimulation waveform to test electrode resistance:

Name & Description	[illegible]		
Shortpulse - Short negative pulse	[illegible]		
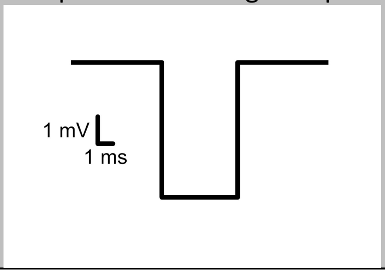	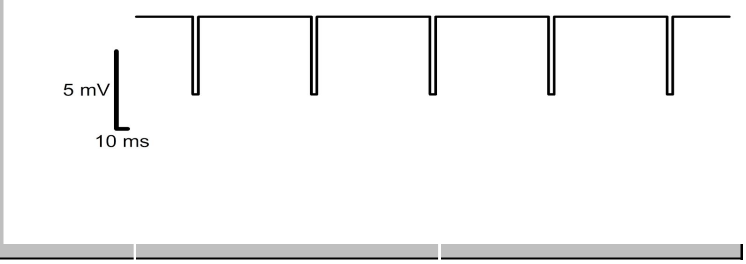		

Stimulation waveform to analyze the physiological properties of patched neurons:

Name & Description	Length	Amplitude	Repeat interval
IVCC - Incremented long pulse	800 ms	-100 pA	0 s
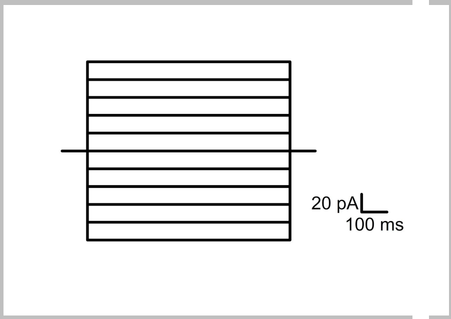		increment: 20 pA	

Batch commands for controlling the HEKA EPC amplifiers:

PROTOCOL "RESET"

This protocol is used for resetting the oscilloscope window in PatchMaster and switch the amplifier to voltage clamp mode.

```
Command   ( 0.000s): "  O  DispTrace       0; Trace 1"
Command   ( 0.000s): "  O  ResetY"
Command   ( 0.000s): "  O  ResetX"
Command   ( 0.000s): "  O  YScaleInc"
Command   ( 0.000s): "  O  YScaleInc"
Command   ( 0.000s): "  O  YScaleInc"
Command   ( 0.000s): "  O  DispTrace       1; Trace 2"
Command   ( 0.000s): "  O  ResetY"
Command   ( 0.000s): "  O  ResetX"
Command   ( 0.000s): "  O  YScaleInc"
Command   ( 0.000s): "  O  YScaleInc"
Command   ( 0.000s): "  O  YScaleInc"
Command   ( 0.000s): "  O  DispTrace       0; Trace 1"
Command   ( 0.000s): "  E  Ampl3           TRUE"
Wait      ( 0.000s): abs  100.0ms
Command   ( 0.000s): "  E  Ampl1           FALSE"
Wait      ( 0.000s): abs  100.0ms
Command   ( 0.000s): "  E  Mode            3; Whole Cell"
Wait      ( 0.000s): abs  100.0ms
Switch    ( 0.000s): "Oscilloscope"
Switch    ( 0.000s): "Amplifier"
Wait      ( 0.000s): abs  100.0ms
```

PROTOCOL "bHunt"

This protocol is used for creating a new group in the file structure in PatchMaster, set the test pulse, set the gain of the amplifier, calculate the offset potential of the electrode, set the filter, and call the “shortpulse” protocol. This protocol is used when the pipette is in the bath. The “shortpulse” protocol is running during the cell hunting phase, and the recorded current signals measured with the NI board to calculate the pipette tip resistance.

```
Command   ( 0.000s): "  @  File          "New Group""
Chain     ( 0.000s): "RESET", return
Command   ( 0.000s): "  E  Reset"
Command   ( 0.000s): "  E  Mode              3; Whole Cell"
Command   ( 0.000s): "  E  PulseAmp                 -5.0mV"
Command   ( 0.000s): "  E  PulseDur                  5.0ms"
Command   ( 0.000s): "  E  CSlow                  100.00pF"
Command   ( 0.000s): "  E  RSeries                 20.0MOhm"
Command   ( 0.000s): "  E  Gain             7;  0.5 mV/pA ->
medium range"
Command   ( 0.000s): "  E  AutoZero"
```

```
Command   ( 0.000s): "  E  Filter2     7.4kHz"
Command   ( 0.000s): "  E  PulseOn           TRUE"
Command   ( 0.000s): "  E  SaveRpip"
Series    ( 0.000s): "shortpulse","",""
Wait      ( 0.000s): abs  100.0ms
Command   ( 0.000s): "  E  Gain             10;  1.0 mV/pA ->
medium range"
Command   ( 0.000s): "  E  Ampl2           TRUE"
Wait      ( 0.000s): abs  100.0ms
Command   ( 0.000s): "  E  Ampl1           TRUE"
Wait      ( 0.000s): abs  100.0ms
Switch    ( 0.000s): "Oscilloscope"
Switch    ( 0.000s): "Amplifier"
Wait      ( 0.000s): abs  100.0ms
Command   ( 0.000s): "  N  Store           TRUE"

PROTOCOL  "bBreakin"
```

This protocol is used for compensating the fast capacitive transient of the pipette. The protocol is called if the pipette tip resistance higher than 1 GOhm ie. When the gigaseal is formed.

```
Chain     ( 0.000s): "RESET", return
Command   ( 0.000s): "  E  Mode              3; Whole Cell"
Command   ( 0.000s): "  E  Gain             10; 5 mV/pA"
Command   ( 0.000s): "  E  AutoCFast"
Command   ( 0.000s): "  E  AutoCFast"
Switch    ( 0.000s): "Amplifier"

PROTOCOL  "aBreakin"
```

##This protocol is used for the cell capacitance compensation and for Rs compensation. It is called when the whole cell configuration is achieved.

```
Chain     ( 0.000s): "RESET", return
Command   ( 0.000s): "  E  Mode              3; Whole Cell"
Command   ( 0.000s): "  E  Gain             11; 5 mV/pA"
Command   ( 0.000s): "  E  CSlow                  10.00pF"
Command   ( 0.000s): "  E  RSeries                 20.0MOhm"
Command   ( 0.000s): "  E  AutoCSlow"
Command   ( 0.000s): "  E  AutoCSlow"
Command   ( 0.000s): "  E  Gain             11; 10 mV/pA"
Switch    ( 0.000s): "Amplifier"
Chain     ( 0.000s): "IVCC"

PROTOCOL  "IVCC"
```

This protocol is used for switching the amplifier to current clamp mode and start the recording. From this point, all the electrophysiological recordings are automatized in PatchMaster.

```
Command    ( 0.000s): "  N  Store           TRUE"
Command    ( 0.000s): "  E  Ampl1           FALSE"
IF         ( 0.000s): AD-7 > -100.00p
   Command    ( 0.000s): "  E  Mode            4; C-Clamp"
   Command    ( 0.000s): "  E  IHold                  0.0pA"
END_IF
Command    ( 0.000s): "E  Mode            4; C-Clamp"
Command    ( 0.000s): "  O  DispTrace       0; Trace 1"
Command    ( 0.000s): "  O  ResetY"
Command    ( 0.000s): "  O  ResetX"
Command    ( 0.000s): "  O  YScaleInc"
Command    ( 0.000s): "  O  YScaleInc"
Command    ( 0.000s): "  O  YScaleInc"
Wait       ( 0.000s): abs  150.0ms
Value      ( 0.000s): Value-4 =  0.0000
Value      ( 0.000s): Value-3 =  0.0000
SetPgf     ( 0.000s): PgfParam-1 =  0.0000
REPEAT     ( 0.000s): sweeps 0.000s
   Sweep      ( 0.000s): "Long square","",""
   IF         ( 0.000s): Value-3 =  0.0000
      IF         ( 0.000s): Value-1 >  1.0000
         Value      ( 0.000s): Value-4 =  Value-2
         Value      ( 0.000s): Value-3 =  1.0000
      END_IF
   END_IF
   IF         ( 0.000s): Value-1 >  16.000
      BREAK      ( 0.000s): repeat
   END_IF
END_REPEAT
Value      ( 0.000s): Value-4 MUL  1.0000G
SetPgf     ( 0.000s): PgfParam-1 = Value-4
Chain      ( 0.000s): "RHEOBASE"
Command    ( 0.000s): "  E  Mode            3; Whole Cell"
Command    ( 0.000s): "  E  VHold                -70.0mV"
Switch     ( 0.000s): "Amplifier"
```

See more: https://www.heka.com/support/tutorials/tutorials_down/pm_tutorial.pdf (Chapter 14).

Supplementary Note 6: Software Usage and Parameters

Introduction

This document introduces the autopatcher software to the user and explains the most important parameters which can be set from the graphical user interface. The Installation section describes how to set up the system and install the necessary submodules. Then the subsequent sections demonstrate how to use the different functionalities.

Installation

The software is written in Matlab, thus having an installed Matlab R2019b or later is recommended with Image Processing and Computer Vision System toolboxes. Simulator mode works with these licenses, but depending on the hardware setup additional toolboxes or support packages might be required. A webcam needs the support package for USB Webcams, while a FireWire camera requires the Image Acquisition Toolbox and the support package for DCAM 1394 cameras. Our pressure controller system and electrophysiological signal processor uses a National Instruments USB-6009 board which requires Data Acquisition Toolbox and the support package for NI-DAQmx Devices. Initially, the software is configured to run in simulator mode. We provide solutions for the mentioned devices and others can also be applied. If the user has access to some different hardware then developing controllers by inheriting abstract classes makes them available for use with the software. Modification of the existing code is not needed.

The configuration of the software is managed by XML files. The tag values are processed as Matlab code. The configuration supports classes, constructor parameters, most often used data types, and method calls. The main configuration file is vistool_config.xml, but some functionality requires the blindpatcher_config.xml, trainer.xml and prediction_config.xml files. The hardware controllers have to be set up in `<element>` tags before starting the software. Those parameters defined inside the `<properties>` tag are tracked and their most recent values are updated in the file when closing the software.

The autopatcher software requires no other installation than extracting the files to a folder. At this point, the software is ready to be used, however, DIC reconstruction requires an OpenCV and OpenCL installation. Cell detection is a computationally expensive task and a dedicated PC might be required besides the microscope controller if there is no sufficient GPU available in it. To run the detection on a dedicated computer, the predictor `<element>` in the config file has to be changed to PredictorRemote. The detection system can be started on the remote machine by startPredictionServer.m to accept tasks. This script can be configured in prediction_config.xml. If the computer that controls the microscope is equipped with a GPU, then remote prediction is not necessary. In this case, again the predictor `<element>`, but now in the main configuration file should be set. This value can be any class that implements the Predictor abstract class. Currently, there are two models implemented that can be used. PredictorCaffe uses Caffe-Detectnet, while PredictorFrcnn uses FRCNN-Resnet50. The former one is a good choice when the GPU memory is rather small (4GB), otherwise, the latter one performs better.

Starting the software: Main window

The software can be started after changing the Matlab working directory to the location of the extracted files. If the startup.m file has not run automatically it has to be run manually. The main window can be started by running the startVisualizationTool.m script which loads the configuration from vistool_config.xml. The logging system might create a log folder and files with .log extension.

Supplementary Fig. 22 shows the main window in live view mode. The live camera or the loaded image is shown on the left. Around the bottom right area of the image, the z level of the microscope is shown in live mode. On the right side, the most often used buttons can be found. The Live View push-button activates or deactivates the camera. If the Live prediction push button is on the cells are detected in the live camera image. The other buttons are used for visual patch clamping and discussed in the Visual patch clamping section. The menu bar at the top of the window is the starting point for other functions and is discussed element-by-element in the rest of this section.

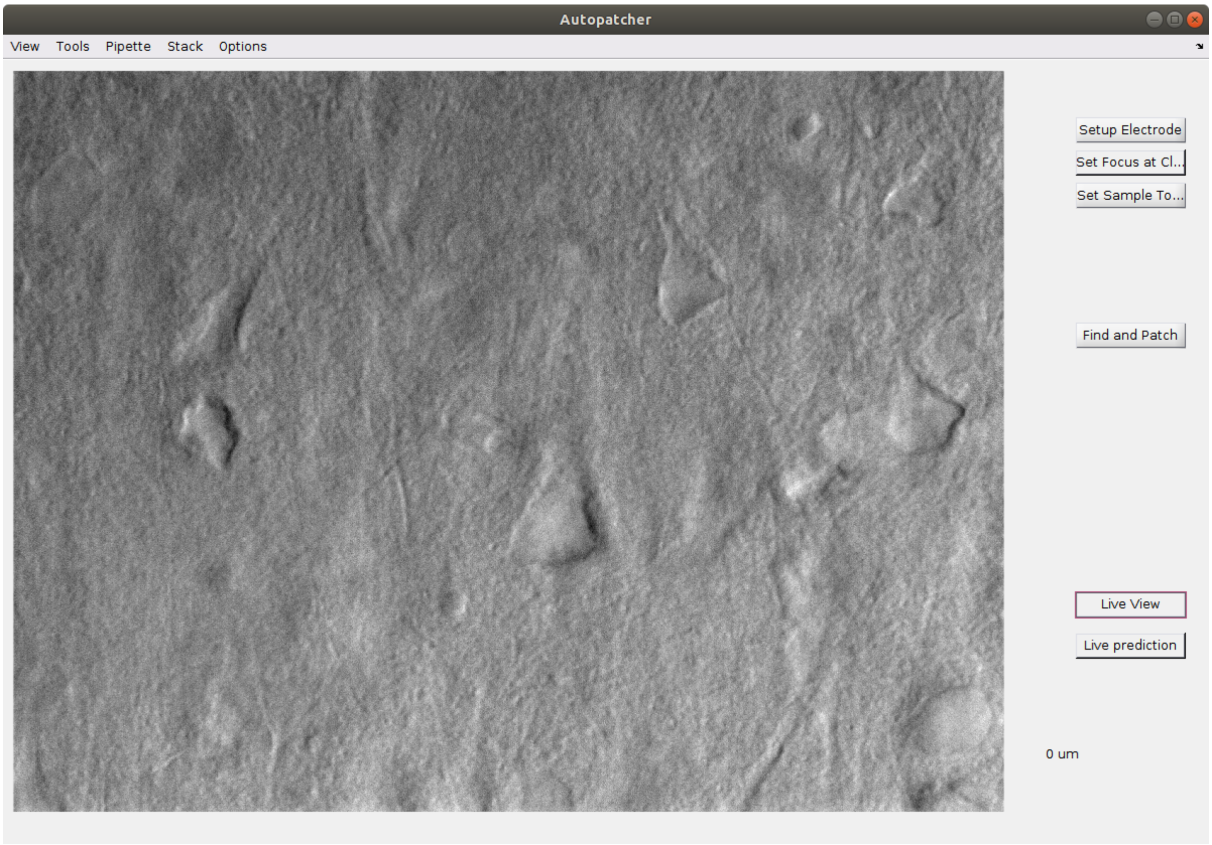

Supplementary Figure 22: The main window of the autopatcher software with live view started.

View menu:

- Blind Patcher: Opens the Blind Patcher window or brings it in focus if already opened.

- Visual Patcher: Opens the Visual Patcher and the Patch Clamp Diary windows, if not opened.
- Cleaner: Opens the Pipette Cleaner window or brings it in focus if already opened.
- Trainer: Opens the Manual3DTrainer window or brings it in focus if already opened and enables its functionality in the main window.

Tools menu:

- Capture and Save image: Saves the current image visible in Live View to a file.
- Reset DCAM: Resets the DCAM driver. Useful if the driver crashes.

Pipette menu:

- Set focus at click: Update the position of the pipette tip by clicking on it in the live camera image.
- Detect focus: Update the tip position of the pipette automatically by a line profile estimation and improve it with the Pipette Hunter 3D 2 wands model.
- Configure: Calibrate the pipette axes for visual patch clamping. This feature should be run after the first startup and before using any visual patch clamping related functionality. A coordinate system transformation has to be determined so that the pipette can be moved based on the microscope's stage coordinate system, or in other words based on the image that we see. This function asks the operator in a step-by-step fashion to move the pipette in every axes at least 100 micrometers (the longer the movements are the more precise the calibration will be) and click on the pipette tip after every movement.
- Ignore sample top: Ignores the sample top position when using the 'Move pipette here' feature in the image and moves the pipette using the 'fast' speed. If the item is unset then the pipette is moved slowly in the tissue and the final step is a forward movement in the x-axis.

Stack menu:

- Load: Loads and shos an image stack.
- Acquire: Acquires and shows an image stack. The number of elements in the stack can be set in the config file under GeneralParameters.stackSize. The current focus level is going to be the topmost image and the step size will be 1 micrometer between the slices.
- Predict: Detects cells in the currently loaded image stack.
- Patch predicted: Offers cells for patch clamping from the recent detection results.
- Show original (default): Shows the original loaded stack (with image normalization).
- Show reconstructed: Performs DIC reconstruction and shows the reconstructed stack.
- Show bg corrected: Performs background correction in the stack and shows the result.

Options menu:

- General options: A window for general options (Supplementary Fig. 23)
  - Camera timer period: The period in seconds to update the live view screen
  - Acquired stack size: The number of images to acquire in an image stack
  - Save Find&Patch stack: Saves the image stack as a file that is acquired by using the Find&Patch button
  - DIC reconstruction group:
    - Iterations: The number of iterations for the algorithm
    - Direction (degrees): Shear direction in degrees
    - Step size weight: The update step size for the gradient descent method
    - Smoothness weight: Multiplier of the smoothness term
    - Cores in a thread: The number of CUDA threads in a thread group

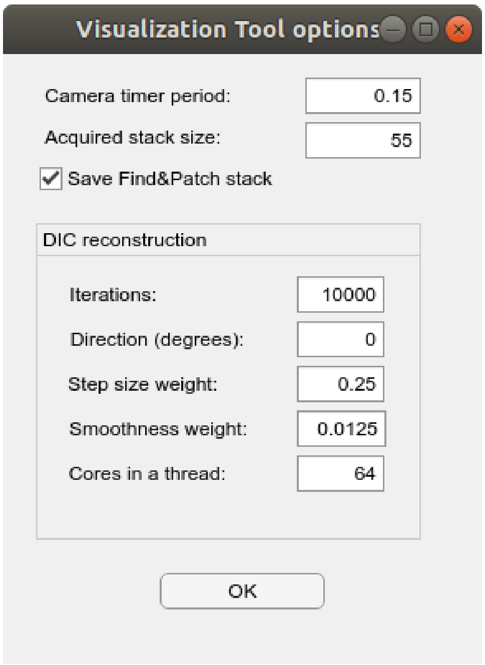

Supplementary Figure 23: General options window.

- Prediction options: Options window for cell detection (Supplementary Fig. 24)
  - Threshold to detect: The detection result is a probability map that is thresholded with this value to get bounding boxes.
  - Prediction timer: The time interval in seconds to run the detection in live view.
  - Minimum/Maximum object width/height: Detected cells with smaller/bigger bounding boxes are not considered as valid results.
  - Minimum overlap to unite: A ratio, if two detections overlap at least this much in different z slices then they are united.
  - Maximum z distance to unite: If the slices, based on their indices, are further from each other than this value they will not be united.

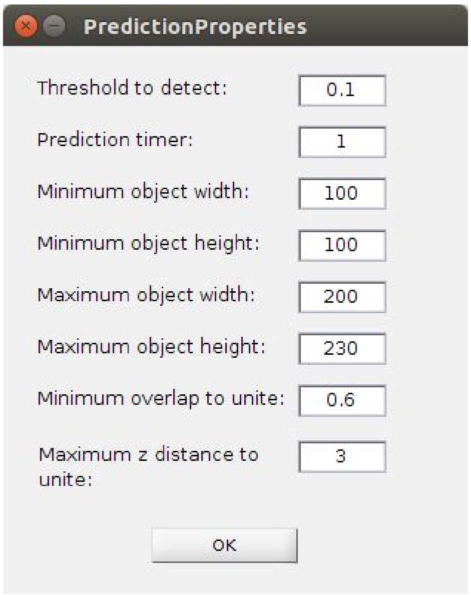

Supplementary Figure 24: Prediction options window.

Blind patch clamping

Blind patch clamping can be initiated from the Blind Patcher window, which is shown in Supplementary Fig. 25. The window contains a Pressure panel to check or manually control the pressure system. Before starting any patch clamping it is recommended to check if the pressure and vacuum sources can provide the preset low/high +/- strengths using the related buttons. It is possible that the sensor does not detect perfectly 0 mBar pressure even if it is physically guaranteed. To compensate for this offset (usually between -10 and +10 mBar) run the Calibrate feature which lasts a few seconds, opens the valves to atmosphere, measures, and saves this offset for later calculations. The Off button disables the pressure controller, which is useful if the operator wants to take over control. Beyond the preset pressure values, the operator can request any reasonable strength by entering the number in the Requested pressure field and pressing enter. The pressure measured on the pipette is visible at all times in the Actual pressure field. The status of the pressure controller can be Regulating (normal operation), Calibration, CalibrationComplete, Disabled, BreakIn and BreakInComplete.

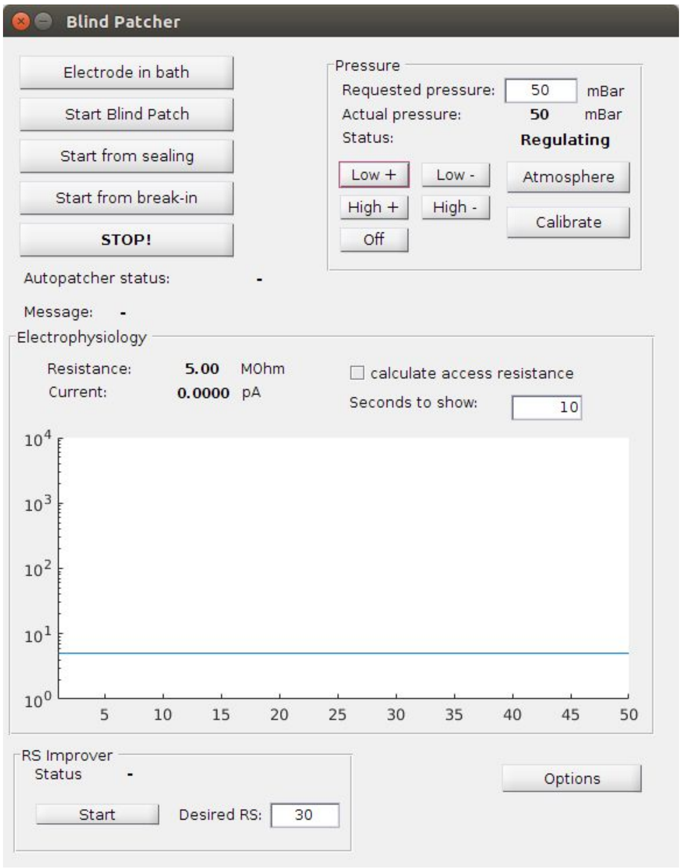

Supplementary Figure 25: Blind Patcher window.

The Electrophysiology panel contains information about the actual resistance and current values and shows the resistance history on a logarithmic scale. Measuring the resistance requires a square command signal which can be activated by the Electrode in bath button in this window.

The Blind Patcher window can be opened in two ways. In both ways it reads the blindpatcher_config.xml file. If it is opened from the Main Window then only the window related properties are used and the control objects are passed to it. It is also possible to perform blind patch clamp recording by starting the startBlindPatcher.m script which opens the window without visual patch clamping capabilities. In this case, the config file has to define the necessary control objects as well.

Performing a blind patch clamp recording starts by clicking on the Electrode in bath button. When pressing this button the pipette should be in the recording solution. This function sets up the pipette by starting a square signal as the command signal for measuring the resistance and applies a small pressure. If the operator finds the resistance value of the pulled glass pipette appropriate, the dura should be crossed by manually moving the pipette and applying higher pressure value. When the pipette is ready for a patch clamp attempt and is near cells the operator has to click the Start Blind Patch button. The current phase of the blind patch clamp process is always visible in the Autopatcher status field in the GUI and can take one of the following three values: Starting, Hunting, Sealing, BreakIn, Success, and Failed. The Message field shows informative details about the current status of the process. In the hunting

phase, the pipette is regularly pushed forward while applying a small pressure until the measured resistance value increases. If a relevant resistance increase is detected then the pipette movement is stopped, the pressure is dropped to atmosphere and the phase is changed to Sealing. In the sealing phase, low negative pressure is applied to form the gigaseal state between the cell and the pipette. If the phase lasts long, the pressure is set to atmosphere for a few short time intervals, the vacuum strength is increased and the pipette is moved a bit in every direction. When the gigaseal state is achieved the phase is changed to BreakIn. In the break-in phase short high negative pressure pulses are applied to break the cell membrane and achieve whole-cell configuration. If the break-in was successful the phase is changed to Success and the recording is automatically started. If any of the phases fail, eg. the pipette is moved too much without hitting a cell in the hunting phase, gigaseal could not be formed in the sealing phase, or the cell died during break-in, then the phase is changed to Failed. If the operator finds a problem at any point in the process which was not detected by the system the attempt can be stopped by clicking on the Stop button. If the problem could be fixed the operator can restart the process from any phase with the Start from sealing or the Start from break-in buttons.

The RS Improver panel contains the Start/Stop button and the Desired RS field and tries to improve the series resistance after a patch clamp attempt by alternating between small pressure and low vacuum.

The Options button opens the Blind Patcher Options window shown in Supplementary Fig. 26. The parameters which can be set here are the following:

- Low positive pressure: Low pressure value used in the patch clamping process.
- High positive pressure: A preset high pressure value (not used in the process but useful for cleaning the pipette tip).
- Low negative pressure: Low vacuum used in the patch clamping process.
- High negative pressure: High vacuum used in the break-in phase.
- ‘Forward’ axis: The axis on which steps are made in the hunting phase
- Hunting group:
    - Min. RS change to seal: Minimum increase in resistance value to switch from hunting to sealing phase
    - Step size: The step size with which the pipette is pushed along its forward axis every second.
    - Maximum distance: The maximum distance the pipette can take in the hunting phase. 0 means no limit.
    - Check hit reproducibility: If checked, pulls back the pipette after a hit; this might increase hit robustness
    - Pull back steps for reproducibility: Number of steps to pull back
    - Clog warning R increase: Warns the user if the R increases by this amount but no hit was detected
- Sealing group:
    - Check R increase on atmosphere: Checks if R increases on atmosphere without applying vacuum, stops otherwise. The amount of increase should be at least as much as ‘Min. RS change to seal’.
- Break-in group:
    - Initial break-in delay: The desired length of first vacuum pulse in the break-in phase.

- Break-in delay increase: The desired increase of the duration of the vacuum pulse after every break-in attempt.
- Gigaseal resistance value: Minimum resistance value which is considered a gigaseal. Measuring higher resistances has higher error rates, thus setting this value higher than the theoretical 1 GOhm is reasonable.
- Successful break-in resistance value: Maximum resistance value which is considered a successful break-in attempt / whole-cell patch.
- Minimum delay before first break-in: Delay between a formed gigaseal state and the first break-in pulse attempt.
- Break-in phase total length: The total length of the break-in phase to allow break-in attempts.
- Pull back after attempts: Pulls back the pipette after this number of break-in attempts. It helps if the nucleus is at the pipette tip and it prevents the formation of a gigaseal.
- Pull back distance: The desired extent the pipette should be pulled back after a few break-in attempts.

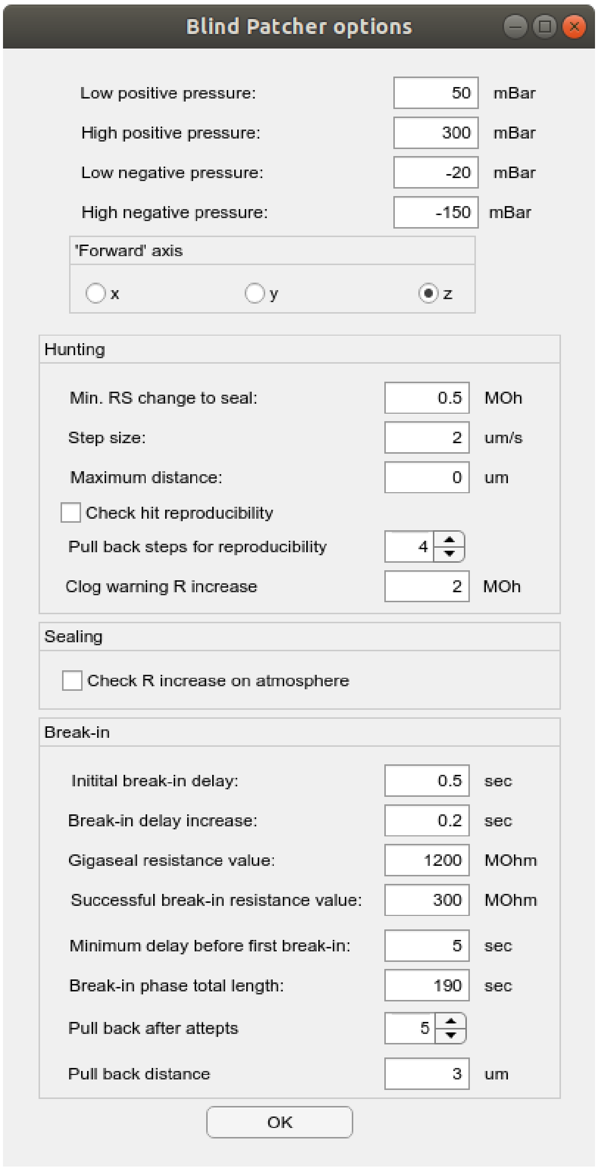

Supplementary Figure 26: The Blind Patcher options window.

Visual patch clamping

Visual patch clamping is the process of visually selecting a cell and performing recording on it. The system automatically tracks the shift of the cell in the tissue due to the deformation caused by pushing the pipette deeper, and avoids obstacles, like blood vessels and other cells, with the pipette on its way to the target.

The most often used way to start visual patch clamp recording is through the buttons on the Main window. It is assumed that the operator has already configured the pipette and moved it in the recording solution close to but above the sample. First, the operator has to click on the Setup Electrode button which applies pressure and starts the square signal. In practice, it has the same functionality as the Electrode in bath button in the Blind Patcher window. Then the pipette tip position has to be updated (especially if a new pipette has been inserted). The more consistent way of updating the tip position is running the Pipette Hunter algorithm from the Pipette menu - Detect focus item. However, depending on the host machine, for an

experienced operator it might be a few seconds faster to click on the Set Focus at Click button and then click on the tip in the camera image. Then the focus level should be set to the sample's top and the Set Sample Top Here button should be clicked to save this position. Finally, the Find and Patch button has to be pushed to run the cell detection and offer cells for recording. The detected cells are offered in descending order of the detection certainty. Supplementary Fig. 27 shows the Main window when the Find and Patch button is pushed and the system offers cells for recording for the operator. The selected cell is highlighted with a dashed bounding box.

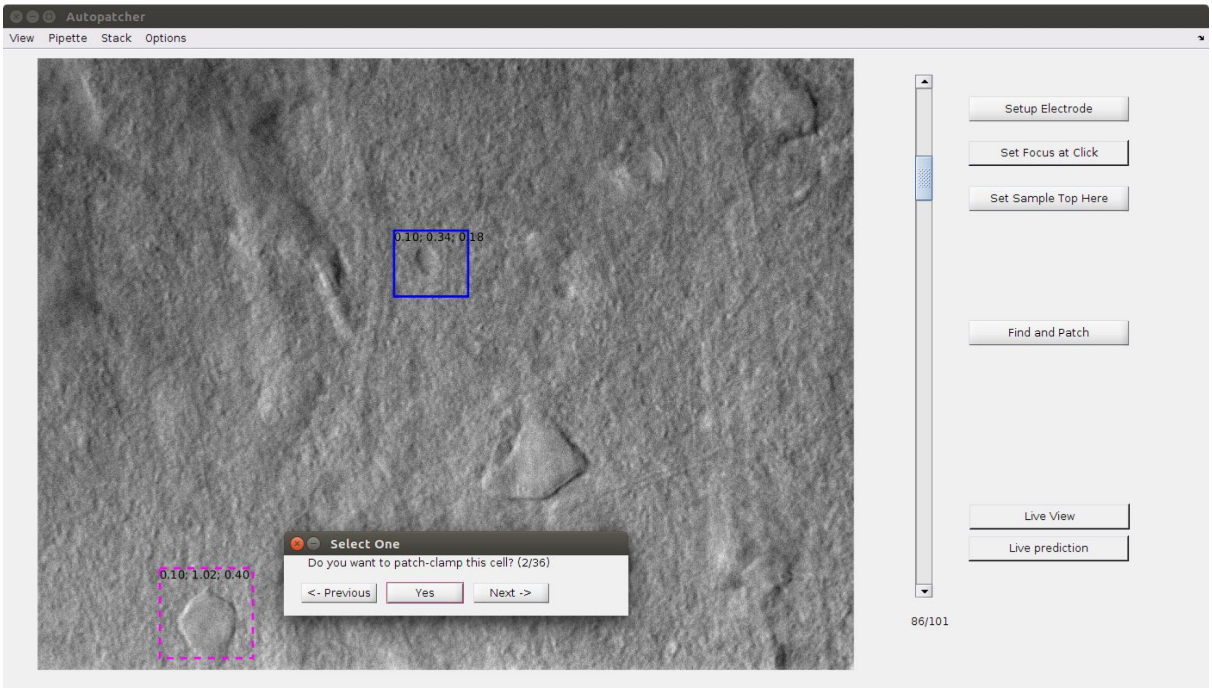

Supplementary Figure 27: Using the Find and Patch feature.

The other way of starting visual patch clamping is to right-click on a cell in the camera image and select the 'Do patch-clamp here' item from the context menu as shown in Supplementary Fig. 28. This approach does not detect cells and the operator should aim to the centroid of the cell to maximize the chance of the pipette hitting the cell.

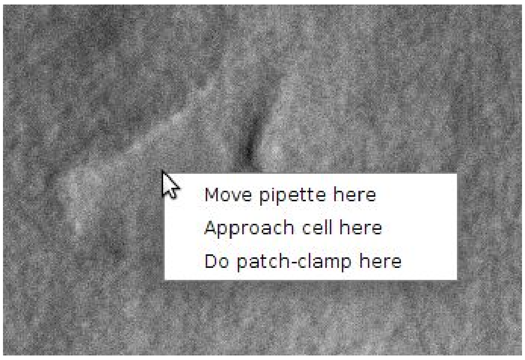

Supplementary Figure 28: Starting visual patch clamp recording without cell detection.

After the visual patch clamping is launched, multiple subsystems are started. The selected cell is centered in the camera image, the pipette is oriented so that only a forward movement in its X axis is needed to approach the cell. The cell tracking system draws a red square around the cell to note which area around the cell is tracked. A middle pressure is applied and the pipette is regularly moved closer to the cell. Meanwhile, the obstacle avoidance system helps to dodge objects on the way to the target cell. The Visual Patcher Control and Blind Patcher windows are opened or brought into focus for the operator to supervise the process. The Visual Patcher Control window shown in Supplementary Fig. 29 contains information related to the mentioned subsystems. When the pipette is just a few micrometers from the target, the Visual Patcher stops and starts the Blind Patcher which performs patch clamp recording. We found that the cell often shifts if the blind patch clamping is performed on the X axis of the pipette. Therefore the system is configured so that the approach places the pipette above the cell and the blind patch clamping is performed by pushing the pipette on the cell from above on the Z axis. However, this behavior can be configured in the configuration file.

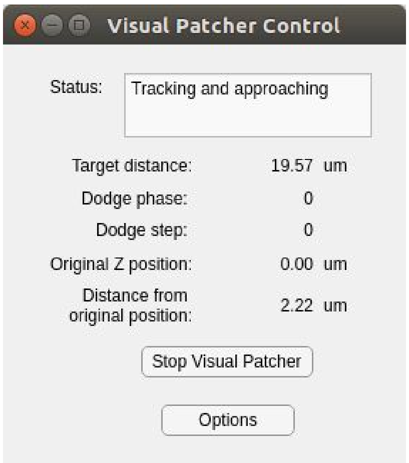

Supplementary Figure 29: The Visual Patcher Control window.

The Options button in the control window opens the Visual Patcher Options window which is shown in Supplementary Fig. 30. The adjustable parameters are detailed below.

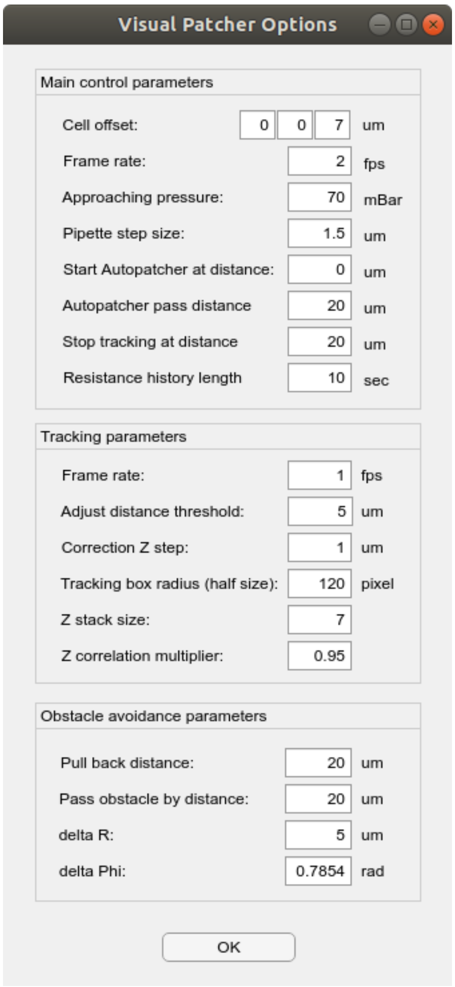

Supplementary Figure 30: The Visual Patcher Options window.

Main control parameters:

- Cell offset: An offset value can be set on all three axes where the pipette will be oriented, relative to the actual position of the cell. This parameter is useful when the forward axis during blind patch clamping is ‘z’ thus a ‘z’ offset should be set a few micrometers above the cell.
- Frame rate: The rate per second when the control function should make a step by pushing the pipette forward, look for obstacles and check the remaining distance to the target.
- Approaching pressure: The pressure value used when approaching the cell. Usually, it is higher than the low positive pressure used for blind patch clamping, since the target can be approached faster.
- Pipette step size: The distance the pipette should be pushed forward when the control callback function is called.
- Start Autopatcher at distance: The distance between the pipette tip and the target cell when blind patch clamping should be started. The control function stops pushing the pipette at this distance. Usually, the value is set in combination with Cell offset. If the blind patcher’s forward axis is ‘z’ and the Autopatcher is started at 0 distance, a cell offset in Z axis of 5-7 micrometers should be used.

- Autopatcher pass distance: The maximum distance the Autopatcher is allowed to take once it is started and before it is stopped by the controller function. 0 means no limit.
- Stop tracking at distance: The distance between the pipette and the target when tracking should be stopped. If the pipette is close to the cell it usually affects the image around it which has negative effects on the tracking system. Usually, this value is set to the same as the Tracking box radius value in the Tracking parameters.
- Resistance history length: The length of the resistance value history, in seconds, that should be kept. This vector is used, for example, to detect obstacles.

Tracking parameters:

- Frame rate: The rate per second the tracking system should make a step at. The tracking steps alternate between xy (lateral) tracking, taking a small image stack, and z tracking in the stack.
- Adjust distance threshold: The tracking system does not correct every little shift of the tracked object but saves this information. The system only moves the pipette when the shift of the target from its original position to the actual one, in micrometers, is more than this value.
- Correction Z step: The tracking system in the Z axis can only indicate whether the target shifted up or down. The correction step of the pipette will be this value if a shift in the Z axis is detected.
- Tracking box radius (half size): Half size of the box in pixels which should be used for tracking in the image. It should be about as big as most cells in the image (thus the box will be twice as big). Depending on the Pipette step size parameter the value can be increased or decreased.
- Z stack size: The number of slices in the z stack which is acquired for tracking in the Z axis.
- Z correlation multiplier: The tracking system uses correlation to determine the shift in the Z axis. However, if the image is noisy the correlation value will also be affected. This multiplier is applied to the correlation value of image slices other than the middle and shift is only detected if they are still higher than the correlation of the middle and the reference images.

Obstacle avoidance parameters:

- Pull back distance: If an obstacle is detected the pipette is pulled back to the extent of this value in micrometers.
- Pass obstacle by distance: An obstacle avoidance attempt is finished when the pipette passes the previously detected obstacle by the distance given in this parameter. Note that this value is not related to the Pull back distance parameter; after the pipette is pulled back it will be pushed forward by both the pull back and pass distances together.
- Delta R: A distance factor used in the formula described with Delta Phi for lateral movement along the axis where the pipette moves during obstacle avoidance[19].
- Delta Phi: A rotation factor used for the formula below for lateral movement along the axis where the pipette moves during obstacle avoidance:

$$m(n) = n\Delta r \cos(n\Delta\Phi - \pi/4)\mathbf{i} + n\Delta r \sin(n\Delta\Phi - \pi/4)\mathbf{j}$$

Patch Clamp Diary

The Patch Clamp Diary subsystem automatically collects information during the use of the software for patch clamping and is able to visually show the attempt positions and generate statistics files. The system intends to replace a paper-based laboratory notebook. Besides the positions, the system saves the outcome and timestamps of the phases of the attempts. The system can be used with or without the visual patch clamping functionalities.

The Patch Clamp Diary window can be opened along with the Visual Patcher window from the main window using the View - Visual Patcher item. The window has three tabs: Slice info, JEM slice info, and Patch-Seq Pipette. Supplementary Fig. 31 shows the window with the Slice info tab activated which enables the marking of the sample's side and holder positions and displays them along with the positions of patch clamp attempts.

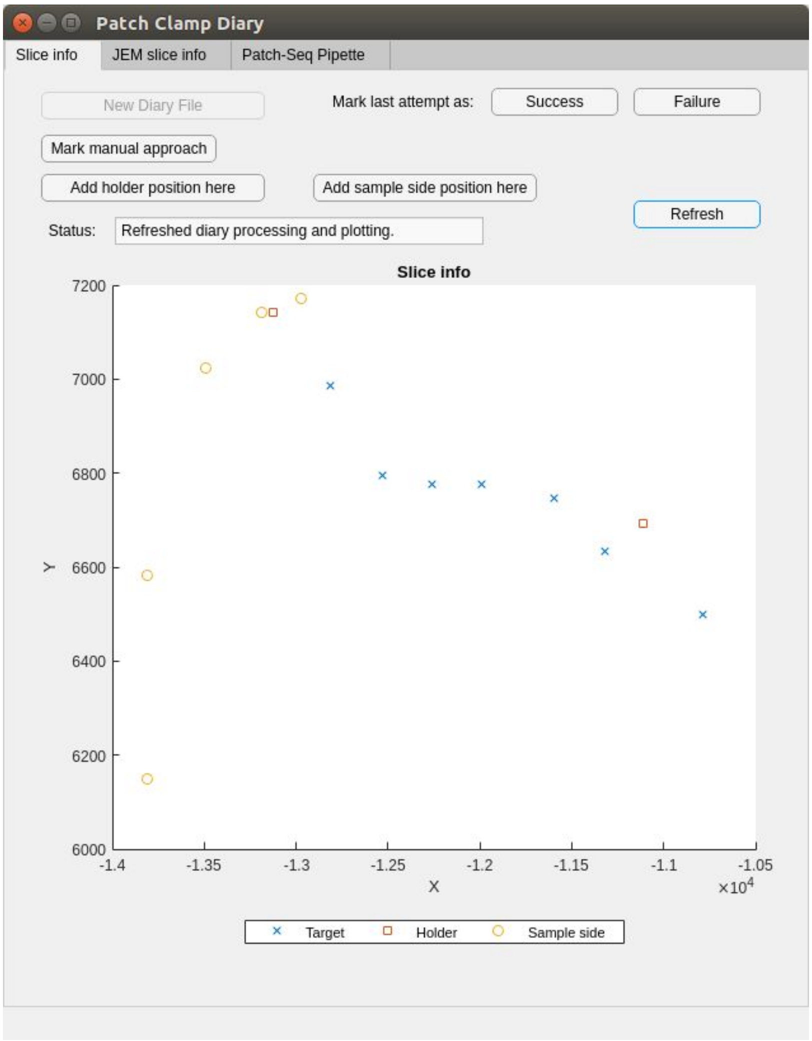

Supplementary Figure 31: Patch Clamp Diary window when the Slice info tab is active.

The Slice info drawing shows the saved positions and has a figure legend. The Status field shows useful information about the function being used. For example, when a holder position is added the Status field outputs the saved position or shows an error message if the position could not be queried but does not interrupt the work of the operator. The role of the buttons and elements in the Slice info tab is detailed here:

- New Diary File: Generates statistics and saves it to a CSV file. It also makes a backup file of the raw log data with the same name but a different extension than the statistics file. Note that this button should be used at the end of a patch clamping session, when finished with a sample or replacing it with a new one, to generate statistics of the last used.
- Mark last attempt as Success/Failure: The outcome of an attempt can be set manually using these buttons. This is useful if the system did not detect an early break-in or if the operator performed patch clamping manually.
- Mark manual approach: If the operator does not use the system's visual patch clamping functionality (blind patcher only or manual) the position of the new attempt can be set using this button. The saved positions are always in the stage coordinate system and the target cell should be approximately in the center of the image.
- Add holder position here: Saves the current stage position as a holder position. It is a good practice to place the holder approximately in the center of the image before clicking on this button.
- Add sample side position here: Saves the current stage position as a sample side position. It is a good practice to place the side of the sample approximately in the center of the image before clicking on this button.
- Refresh: Generates statistics, saves it to the default file name and updates the Slice info drawing.

The generated CSV file contains column names, most of which are self-explanatory but some of them are explained here. The TargetDepth value is measured from the manually set sample top position, while the TargetDistance expresses the distance the pipette should move on its X axis to reach the target. The abbreviation 'AP' in multiple columns stands for Autopatcher which is the blind patcher subsystem. The DetectionSelectedIndex shows the index of the selected cell when using the Find and Patch function. The timestamps are in the host system's timezone. Positions and distances are expressed in micrometers, resistance values are in megaohms.

The JEM Slice info and Patch-Seq pipette tabs are used together to document nucleus harvesting attempts. Supplementary Fig. 32 shows these tabs. Most values should be filled manually but there are some which are filled automatically, e.g. timestamps and resistance values.

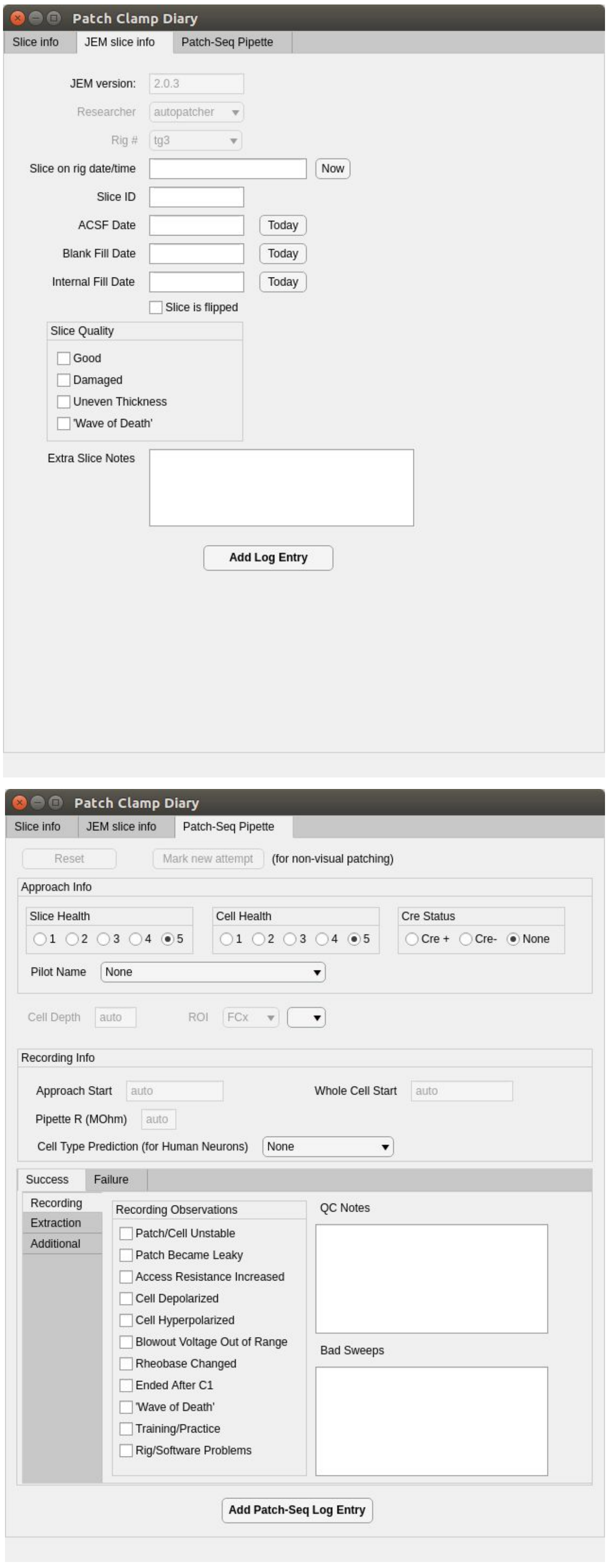

Supplementary Figure 32: The JEM slice info and Patch-Seq Pipette tabs.

Pipette Cleaner

The pipette cleaning functionality[20] can be configured and used from the Pipette Cleaner window which can be opened from the main window's View - Cleaner item and is shown in Supplementary Fig. 33.

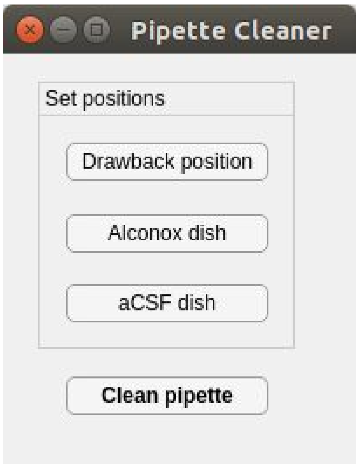

Supplementary Figure 33: The Pipette Cleaner window.

Before cleaning a pipette with the functionality the cleaning detergents have to be installed around the sample. Two detergents are required: standard recording solution (aCSF) and Alconox. We have designed and 3D printed an object, shown in Supplementary Fig. 34, that can hold two 250 µl PCR tubes containing the detergents and can be attached to the objective. Other tube holders or even Petri dishes can be used.

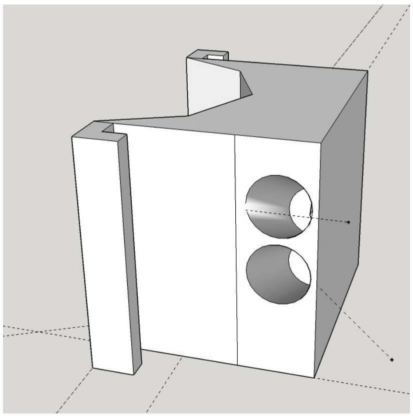

Supplementary Figure 34: Schematic of the 3D printed object that holds the cleaning detergents.

When the detergents are installed and the objective is lowered to the position where a sample would be visible, the operator should make sure that the pipette can reach the tubes and make adjustments if necessary. Then the pipette has to be calibrated for the dish positions using the GUI which are saved and loaded the next time the software starts. The Drawback position button saves the X position of the pipette which should be set such that the objective or other objects are not hit around the tubes when moving the pipette in the other axes. Then the pipette should be manually moved to the tube containing Alconox such that the tip is immersed with the detergent. The Alconox dish position button saves the position of the pipette. This has to be repeated for the other liquid before clicking on the aCSF dish button. Then the pipette can be cleaned after patch clamp recording attempts by the Clean pipette button. This button starts the process by pulling back the pipette to the drawback position, adjusting it laterally before pushing it in the dishes and alternates between blowing and suction pulses to clean the pipette. When the cleaning is finished the pipette is moved back safely to the position where the process was initiated.

Image stack labeling tool

We developed a tool for image stack labeling in the main window of the software. The tool allows the user to put 2D bounding boxes around the cells in the images over multiple stacks. We used the labels generated with this tool to teach a convolutional neural network for DIC cell detection. The tool can be started from the main window's View - Trainer item and Supplementary Fig. 35 shows it while a labeling is in progress.

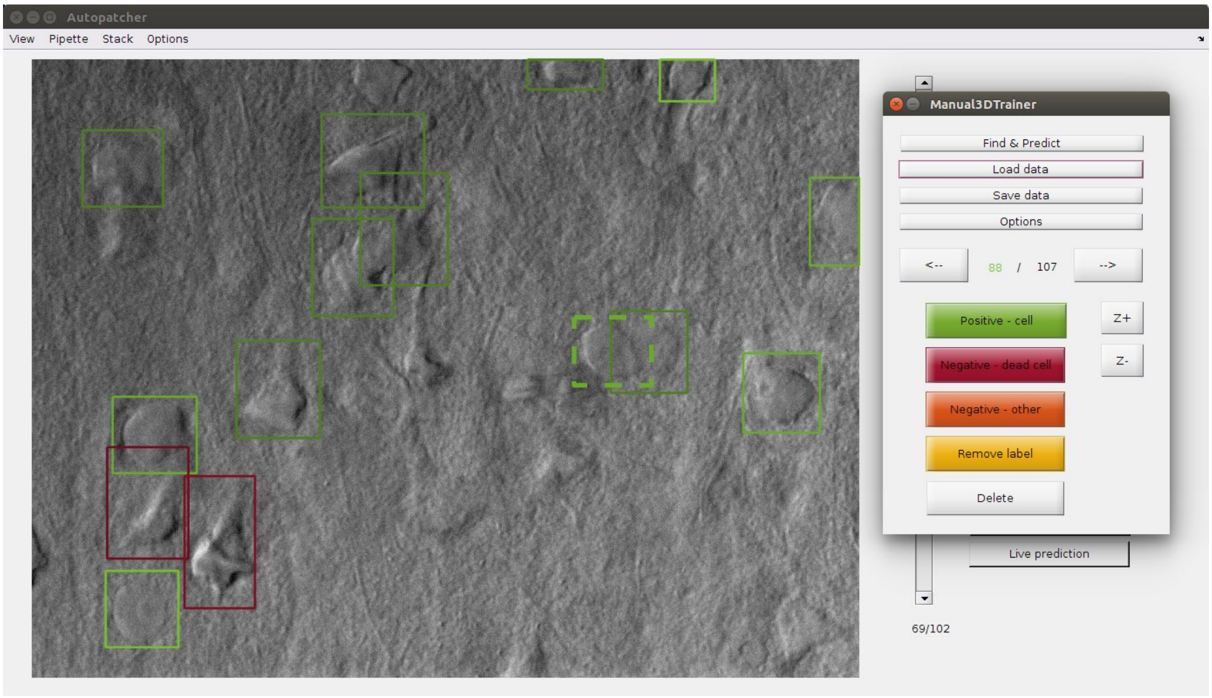

Supplementary Figure 35: The image stack labeling tool.

The tool can only be opened if an image stack is loaded (or acquired) in the main window. When the tool is opened, the labeling functionalities are enabled. Double-clicking in the image adds a new label with a default box size. The dashed lines always indicate the selected box. The edges of the boxes can be adjusted by dragging them with the mouse while the control key is pushed. The extent of the boxes in the Z dimension can be changed globally but not individually. Thus the boxes should be added in the slice where the cell is in focus. However, the Z position of the boxes can be adjusted later. The boxes can have different labels: a positive for cells, a negative for dead cells, and another negative possibly for other objects like red blood cells. A label can also be removed while the box is not deleted which is useful if the user wants to keep it until a later decision. The data can be saved and loaded and if the cell detection system which uses a previous network is installed it can be used to help the user and automatically put bounding boxes on the detected cells. The default new box size and the global size in Z dimension can be set and the values are saved in the trainer.xml file in the config folder.

Below are the description of the buttons in the window:

- Find cells: Uses the cell detection system and puts bounding boxes on the detection results.
- Load data: Loads a previously saved data of bounding boxes.
- Save data: Saves the bounding boxes from the current session to file.
- Options: Opens a dialog where the default box sizes can be set.
- Arrow buttons: Select the next/previous bounding box (dashed line shows the one selected). Arrow keys also switch the selection.
- Z+/Z-: Adjusts the Z position of the selected box.
- Positive - cell: Labels the selected box as positive.
- Negative - dead cell: Labels the selected box as a dead cell.
- Negative - other: Labels the selected box as other negative example.
- Remove label: Removes the label from the selected box but keeps the box itself.
- Delete: Deletes the selected box and its label. The delete key also has this functionality.

Supplementary Note 7: Representative Examples

This note contains plots of pipette trajectory, pressure, and resistance values.

Successful examples

Successful #1

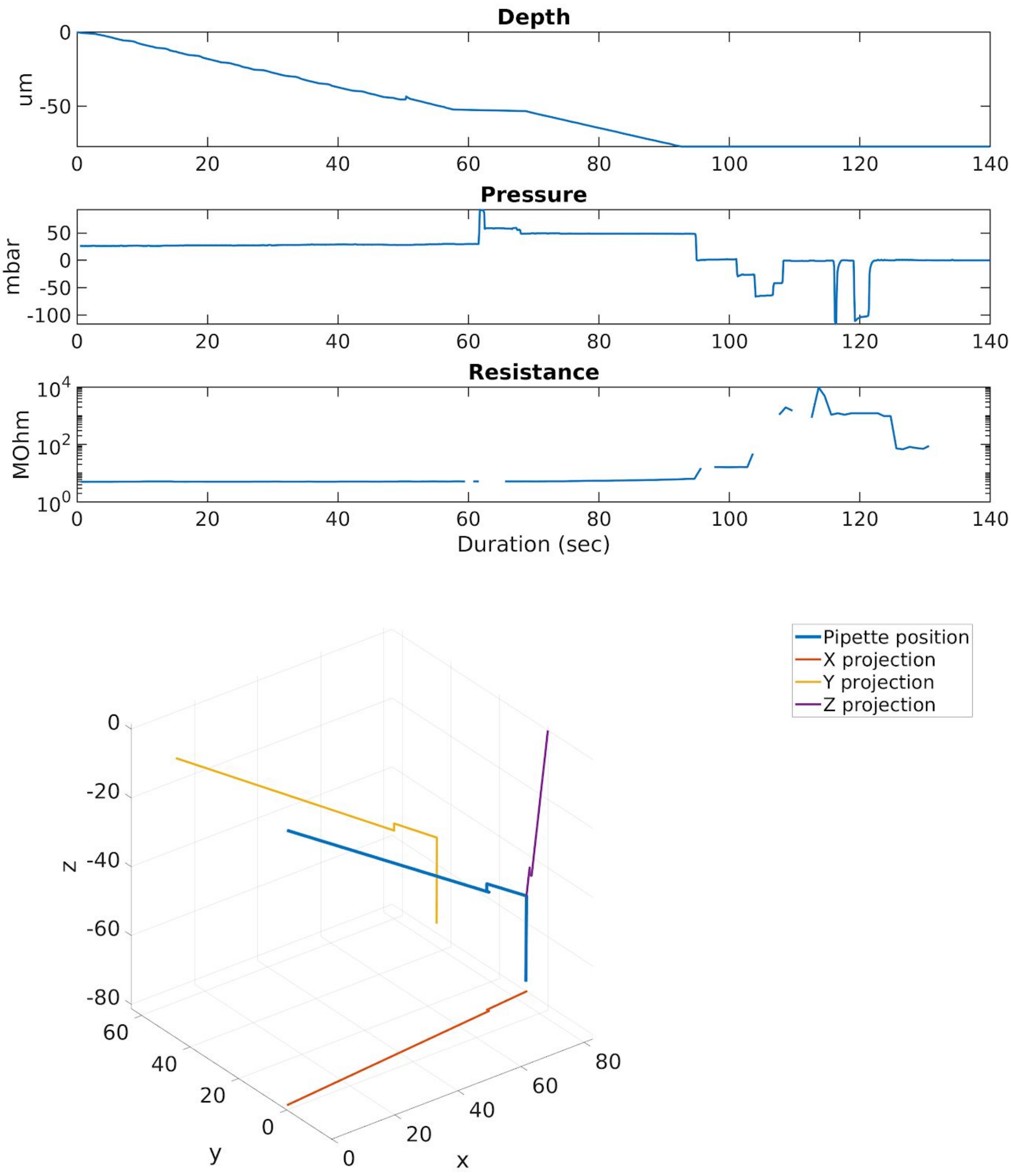

Successful #2

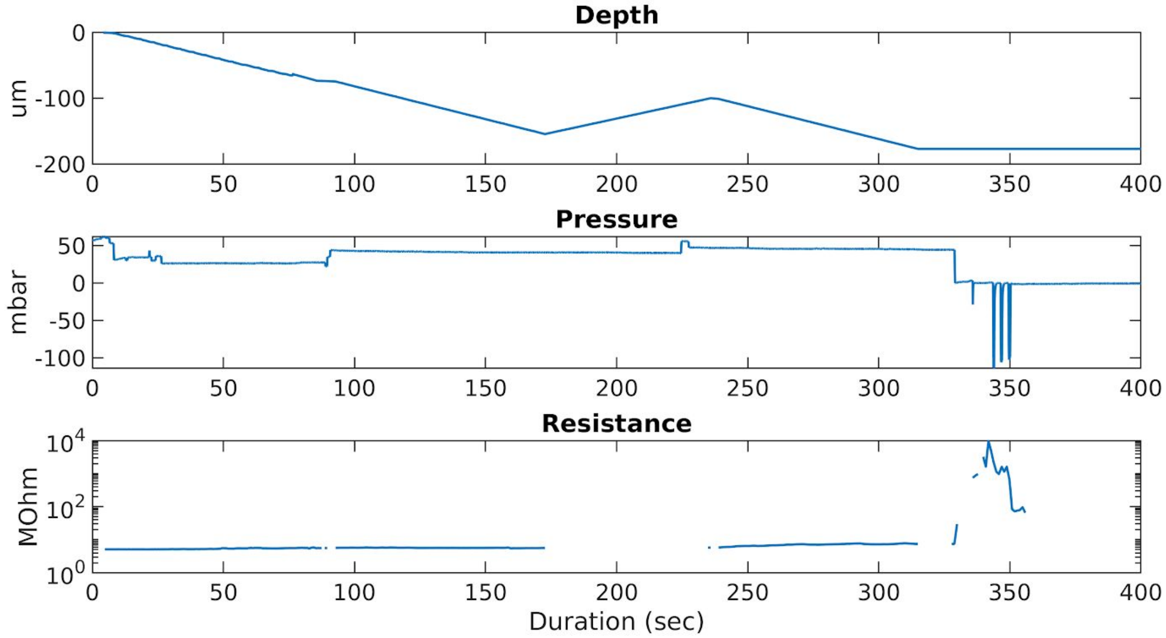

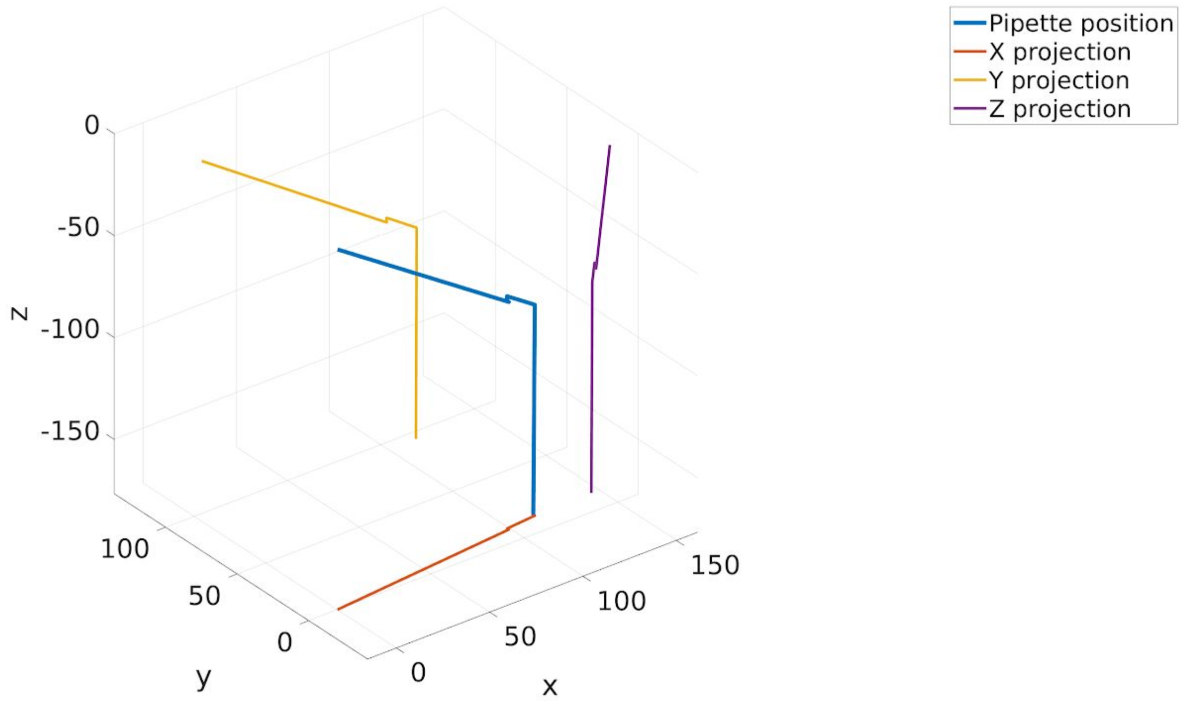

Successful #3

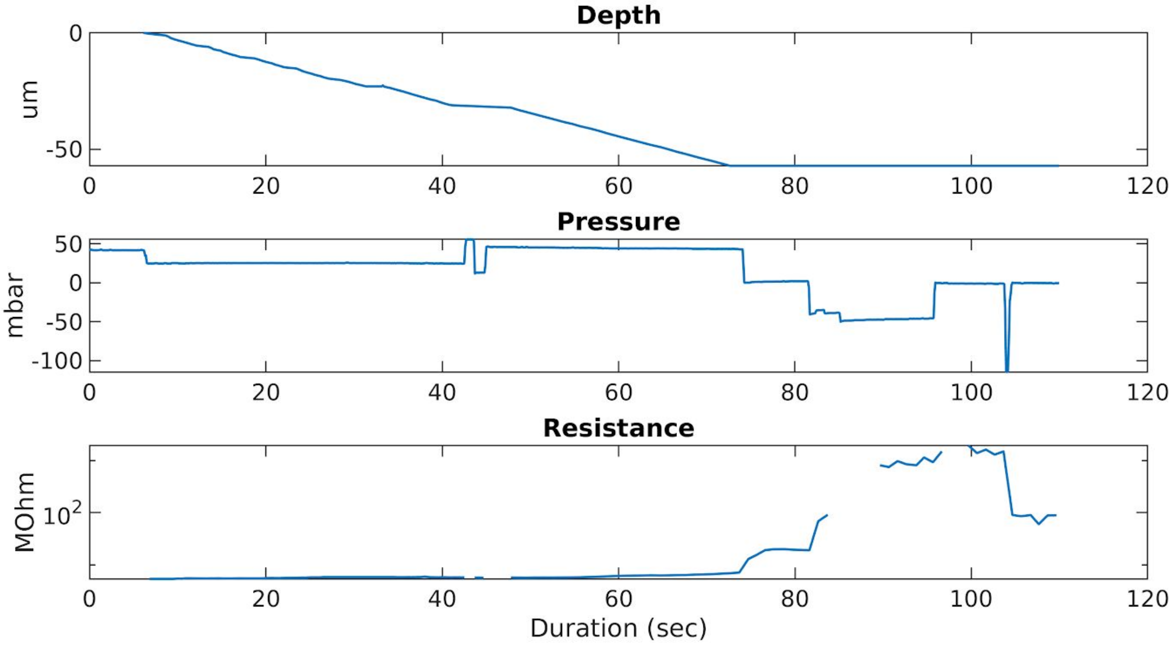

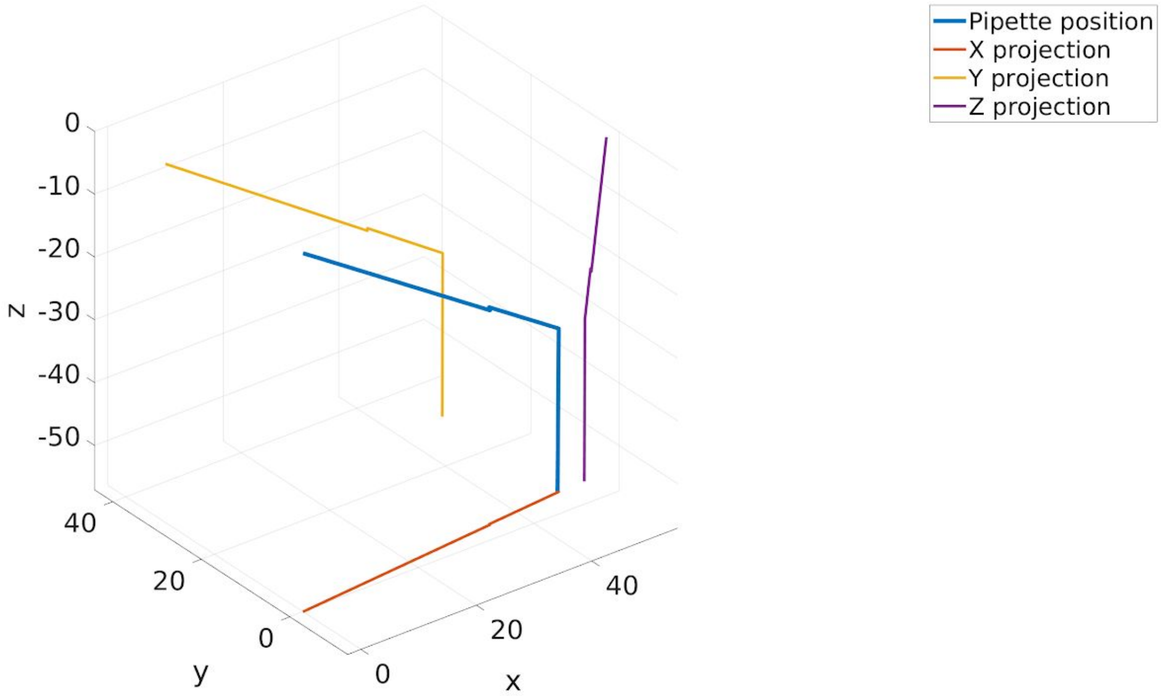

Successful #4

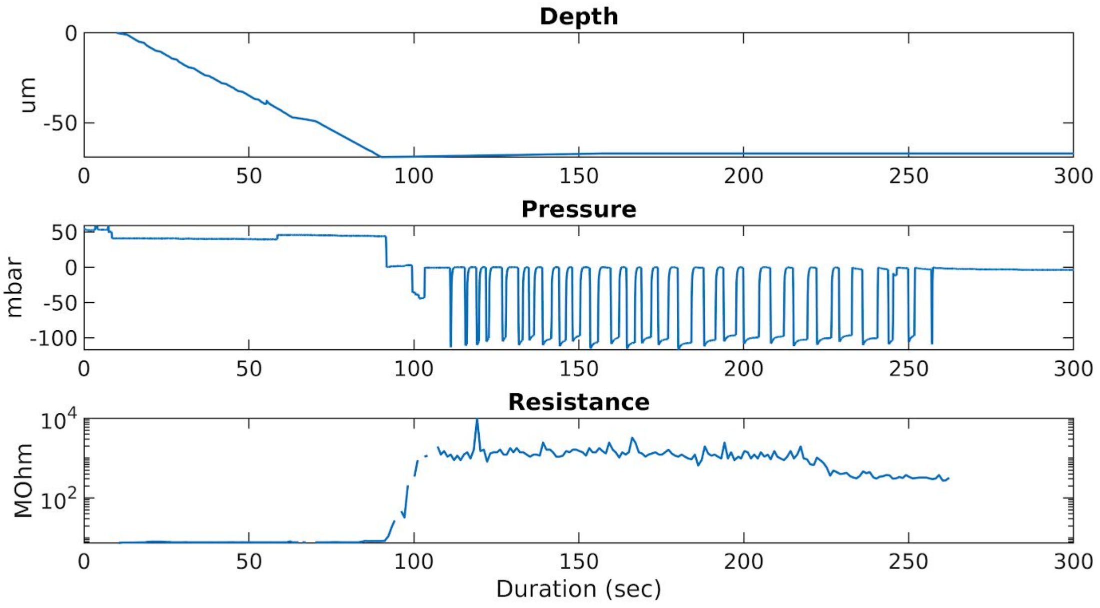

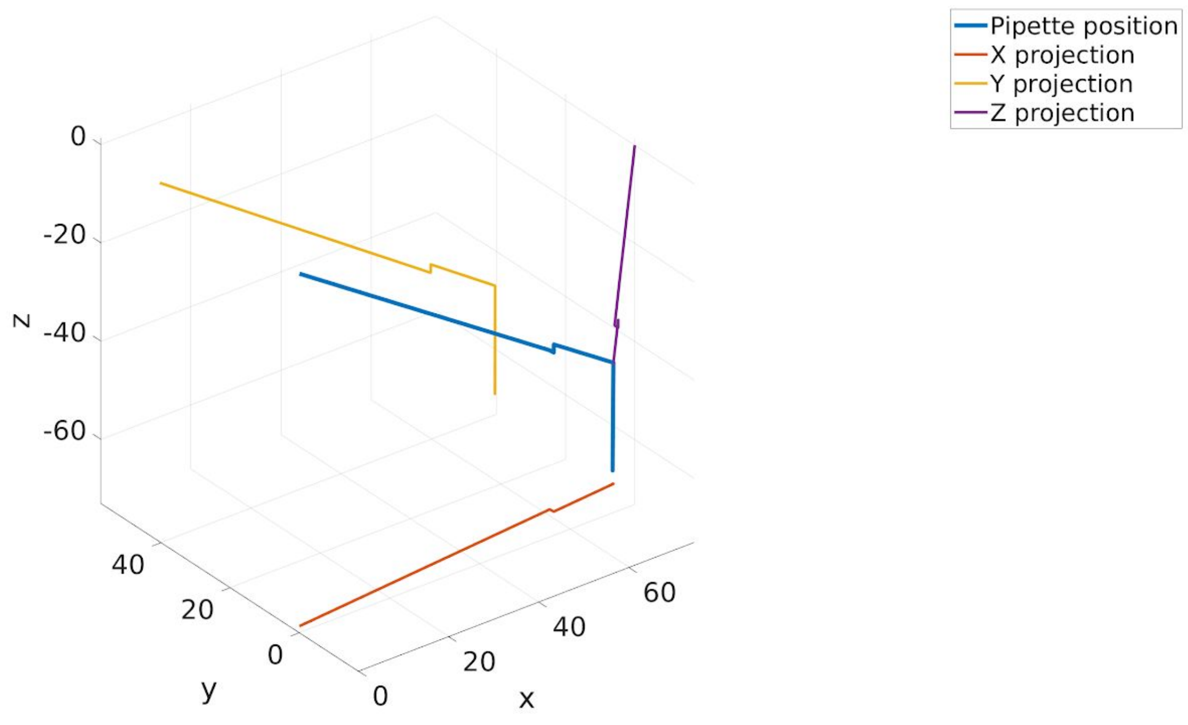

Successful #5

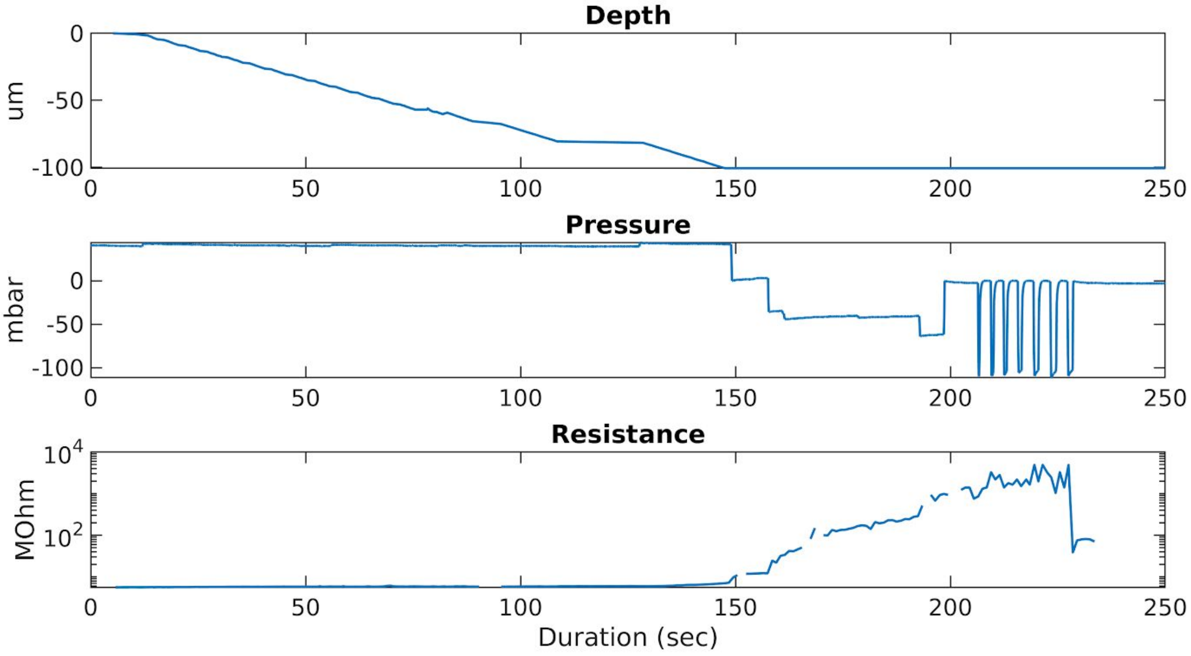

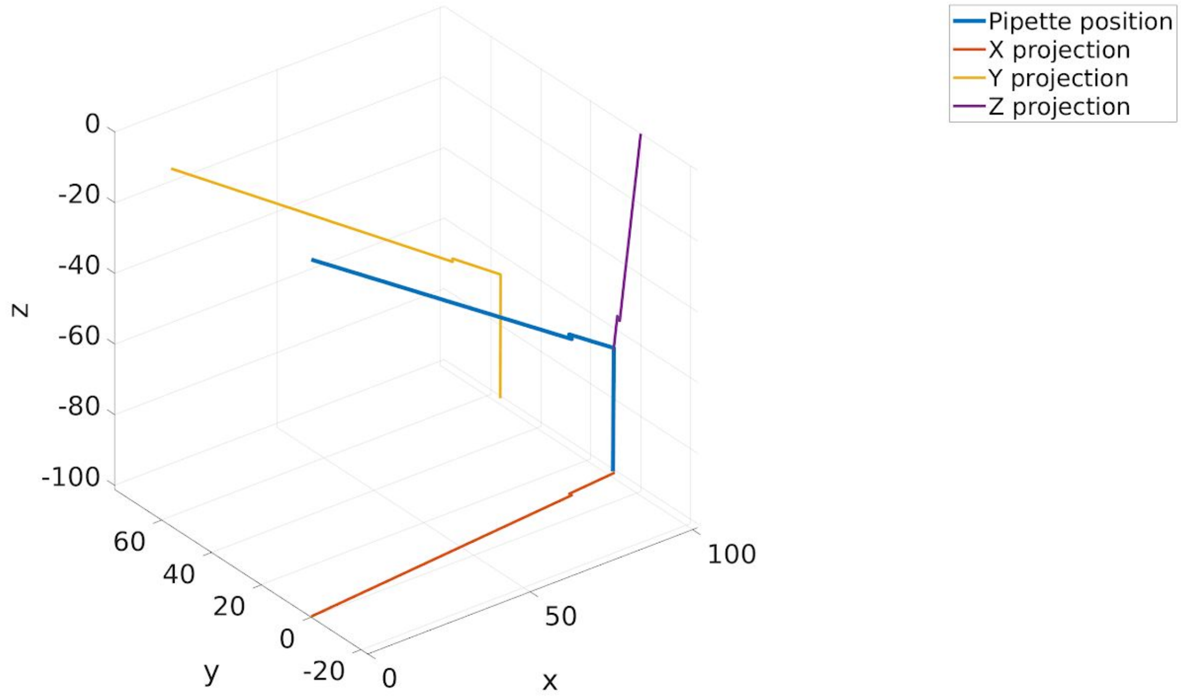

Successful #6

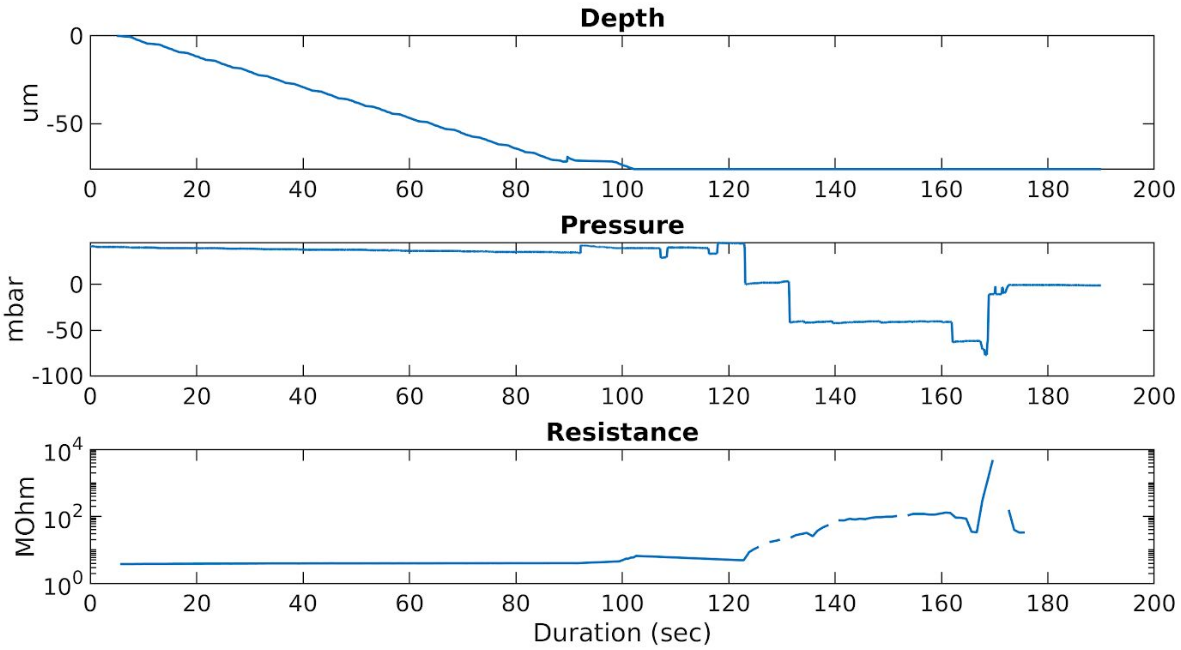

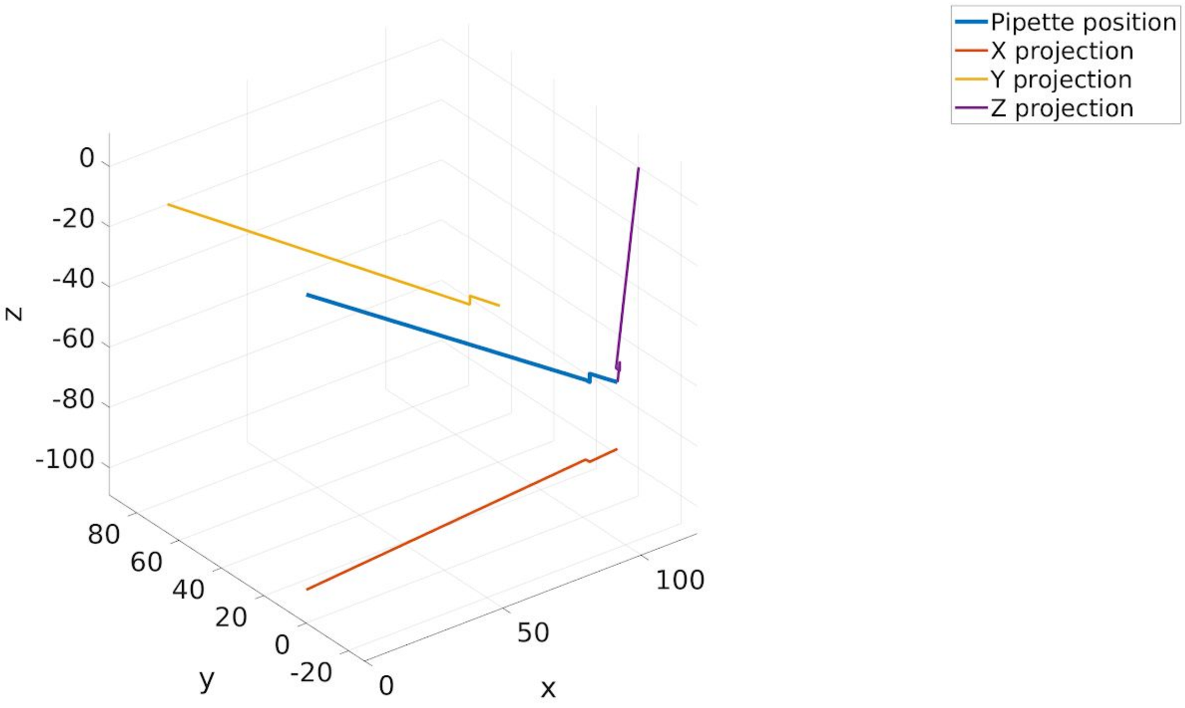

Successful #7

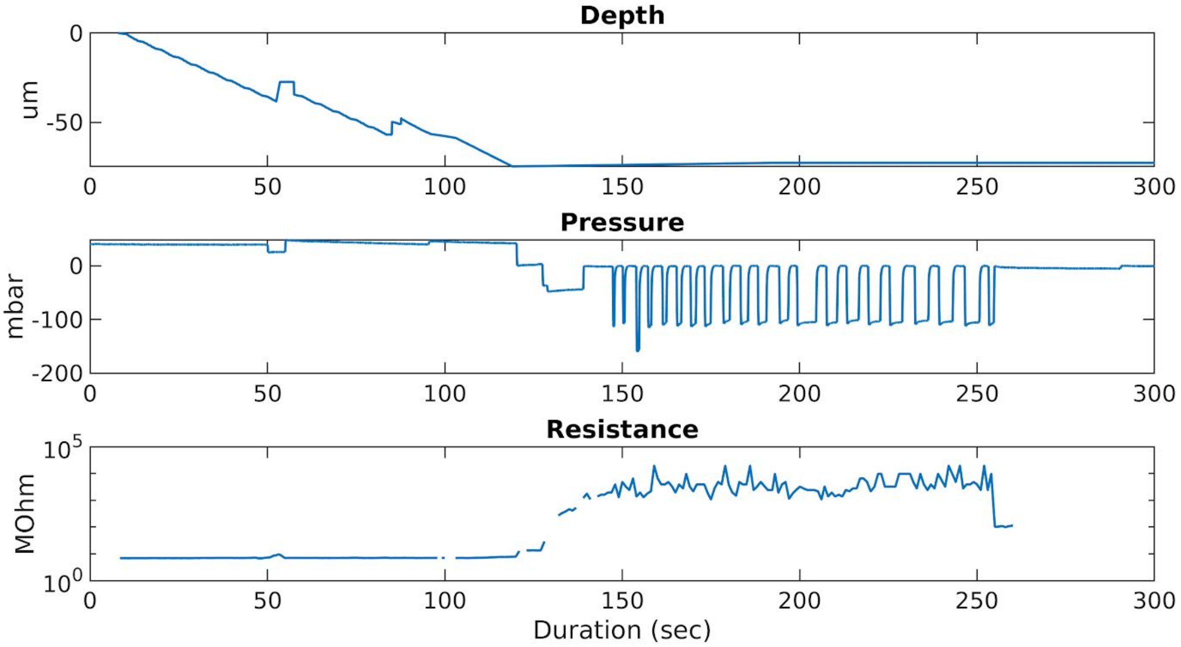

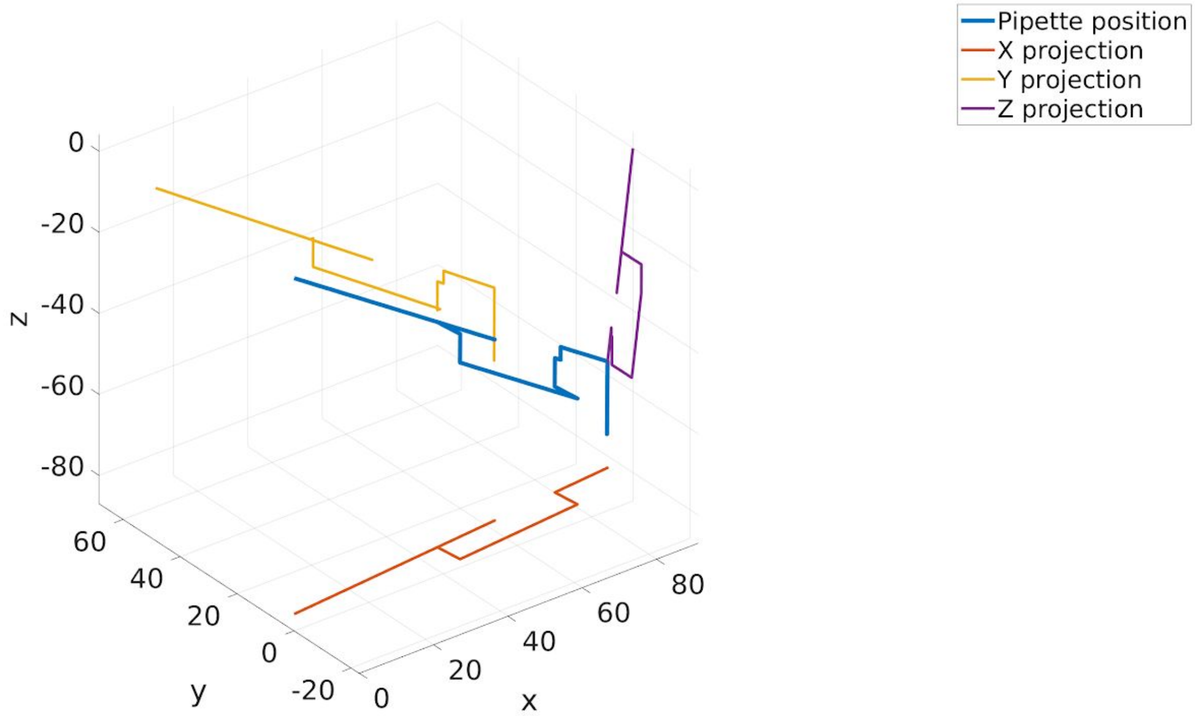

Successful #8: Blind patch clamp attempt, movement only in Z direction.

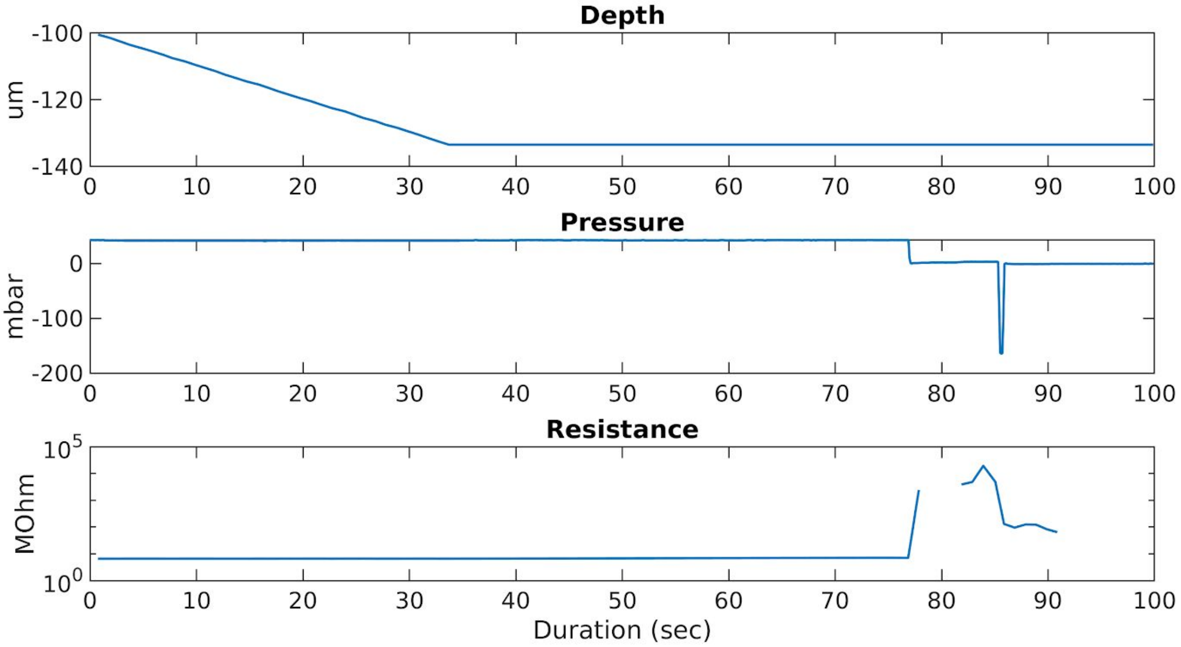

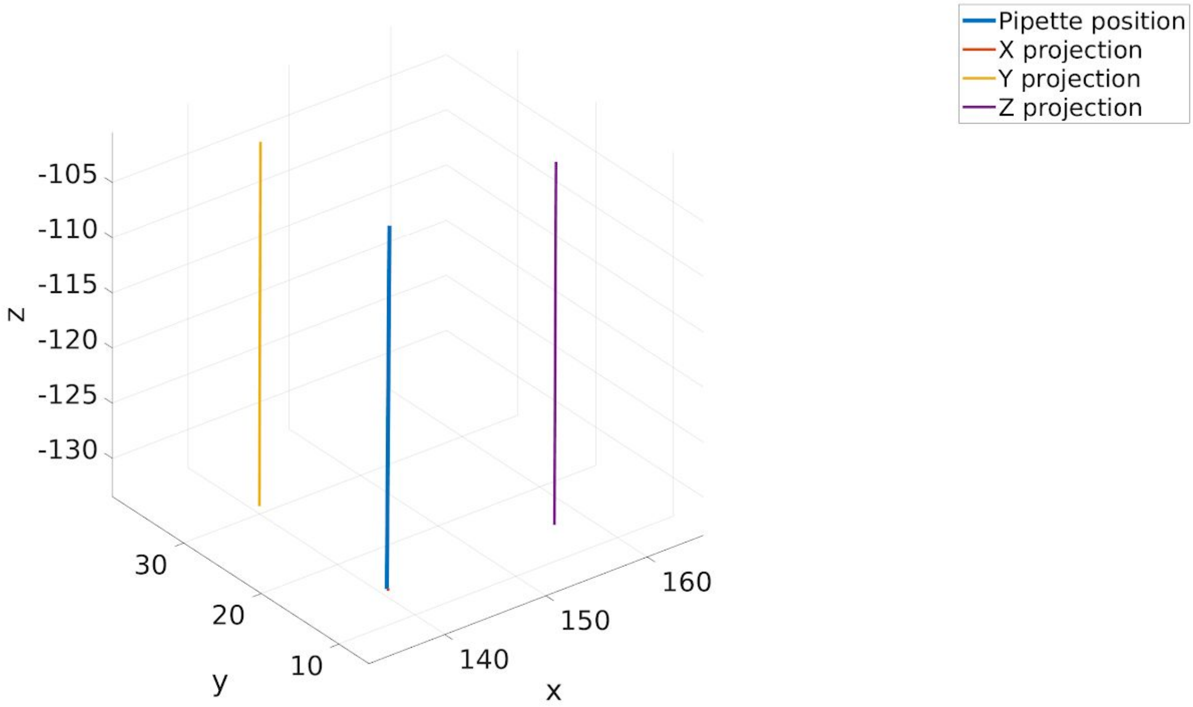

Successful #9: Manually moved the pipette next to the cell, DIGAP started from break-in.

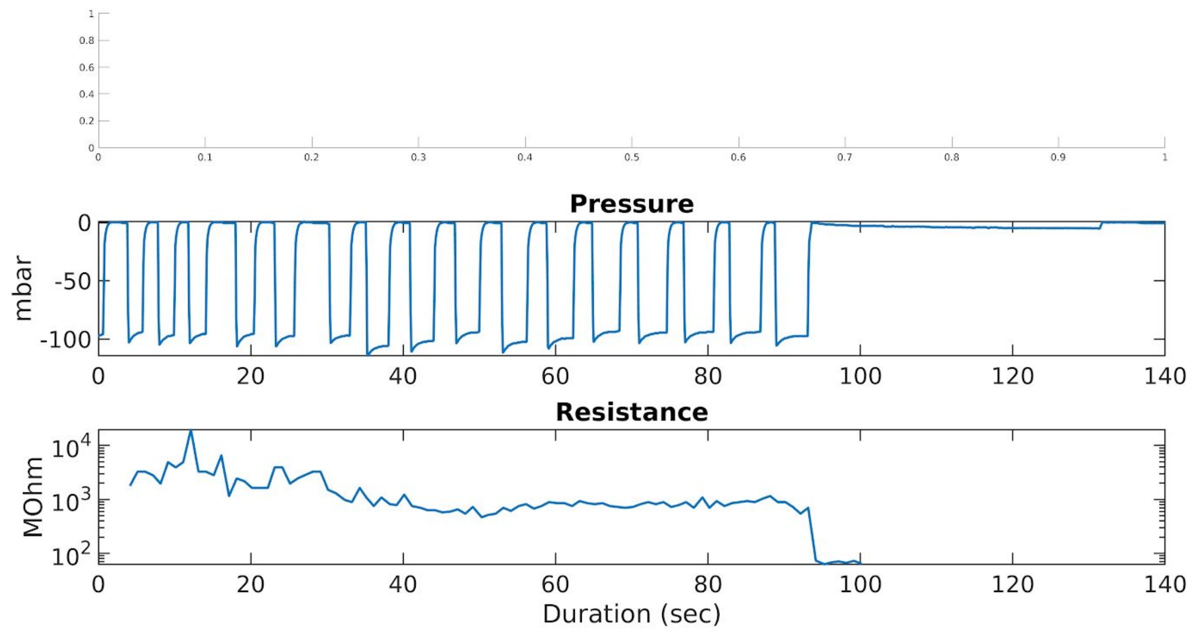

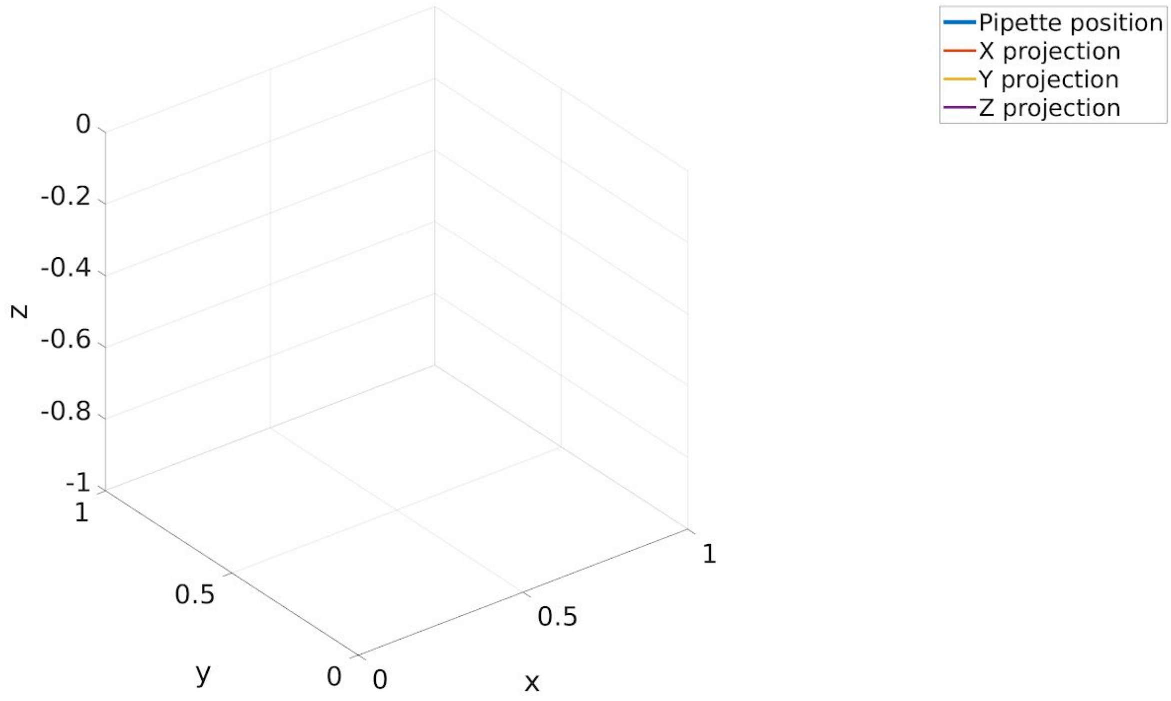

Failed examples

Failed #1

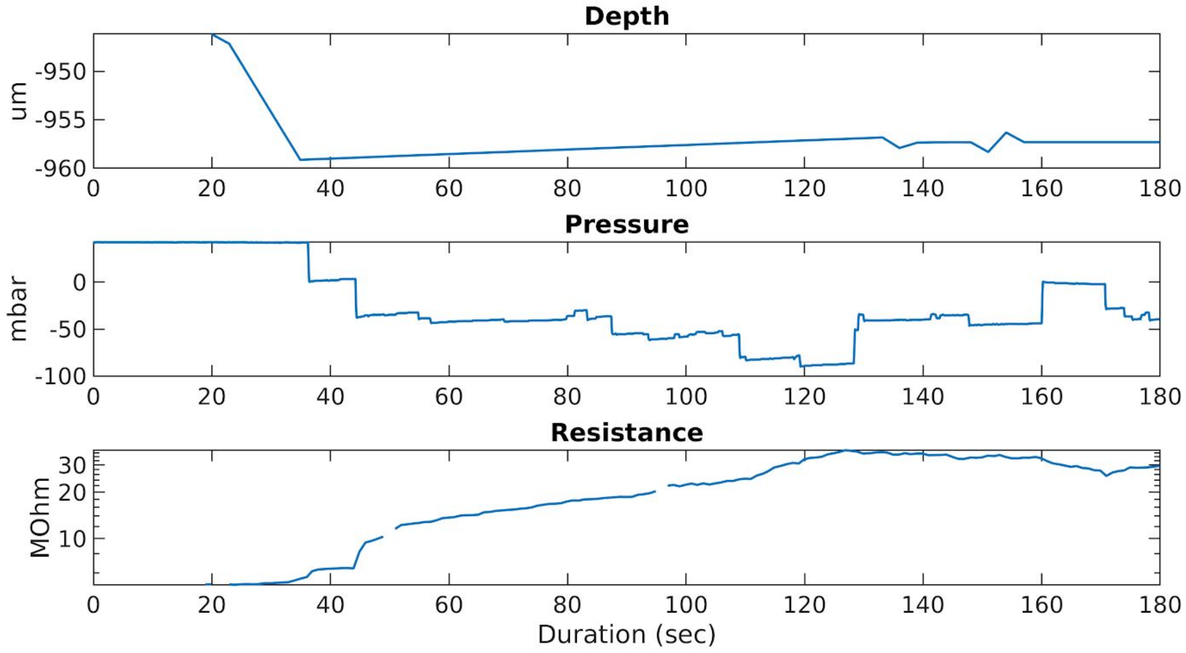

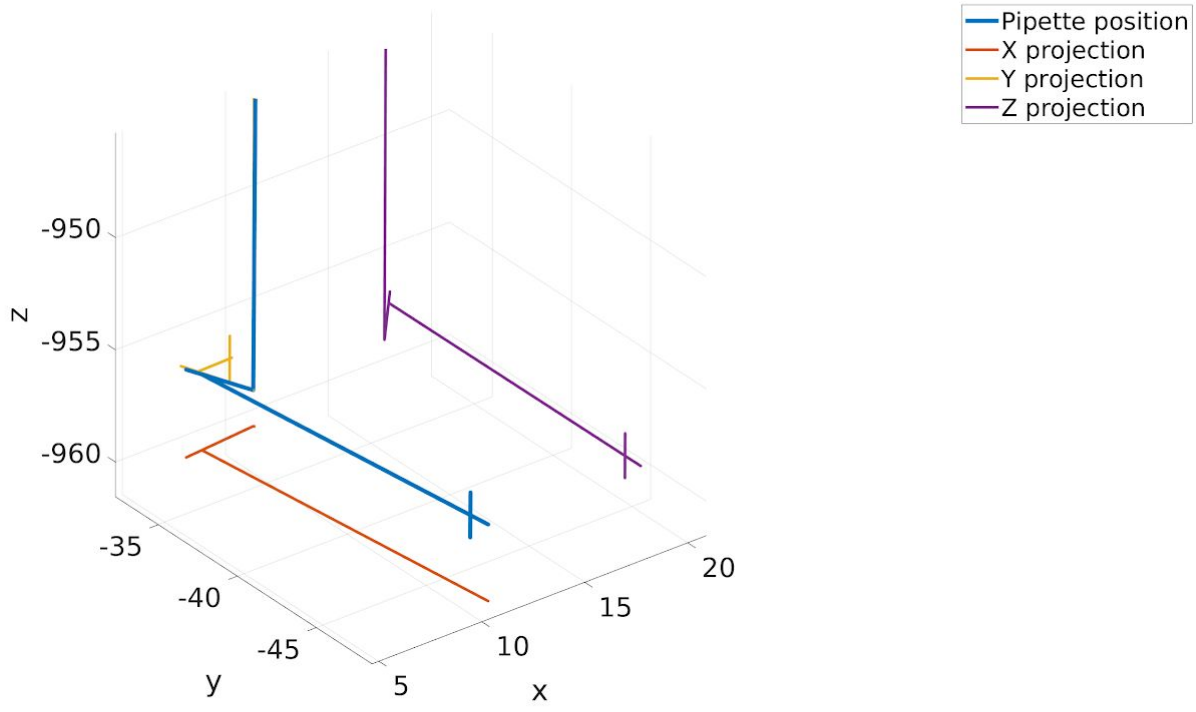

Failed #2: Problems arose in resistance measurement, but suction pulses continue when fixed.

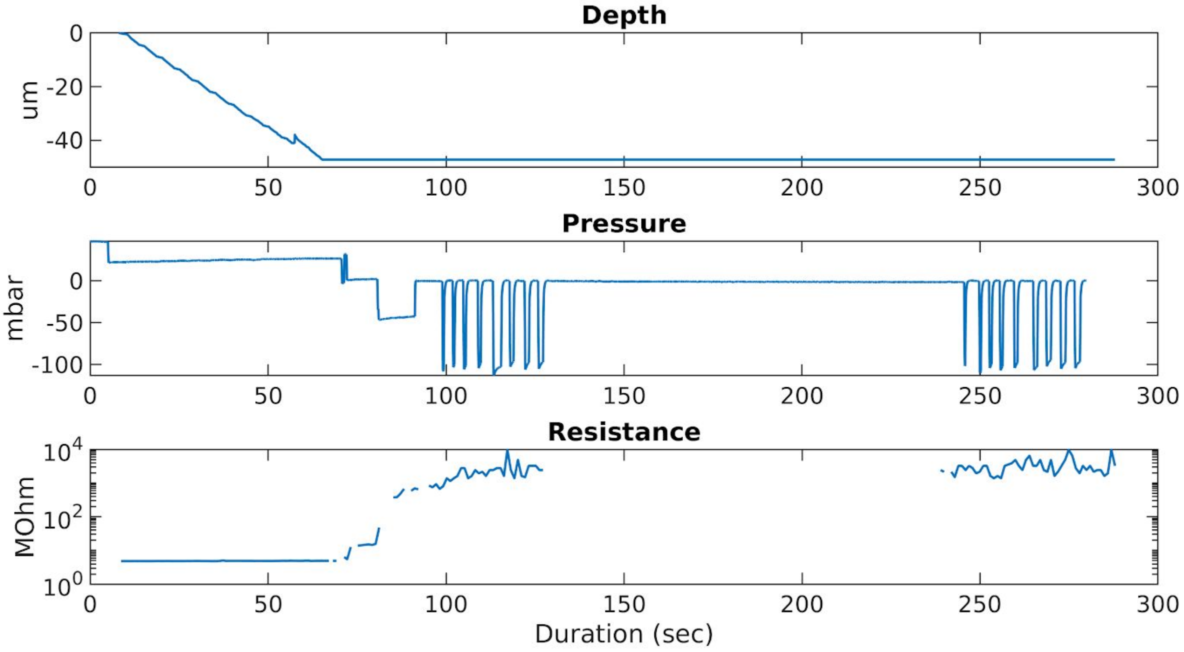

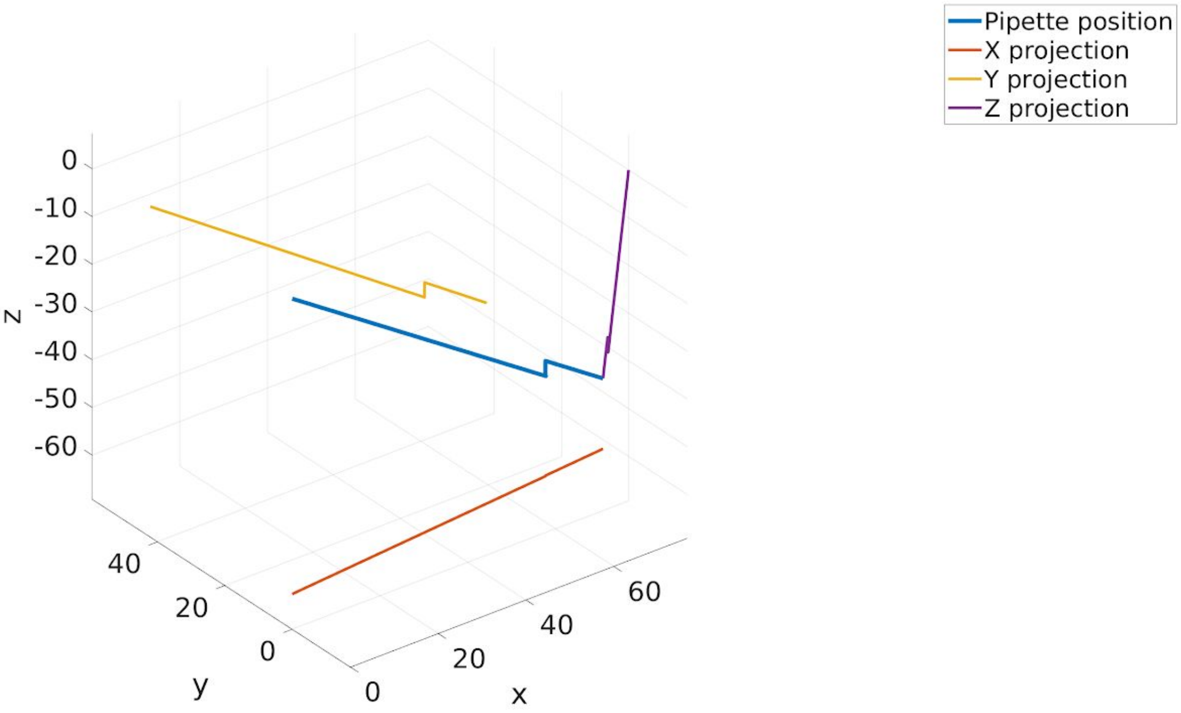

Failed #3: Started from break-in phase.

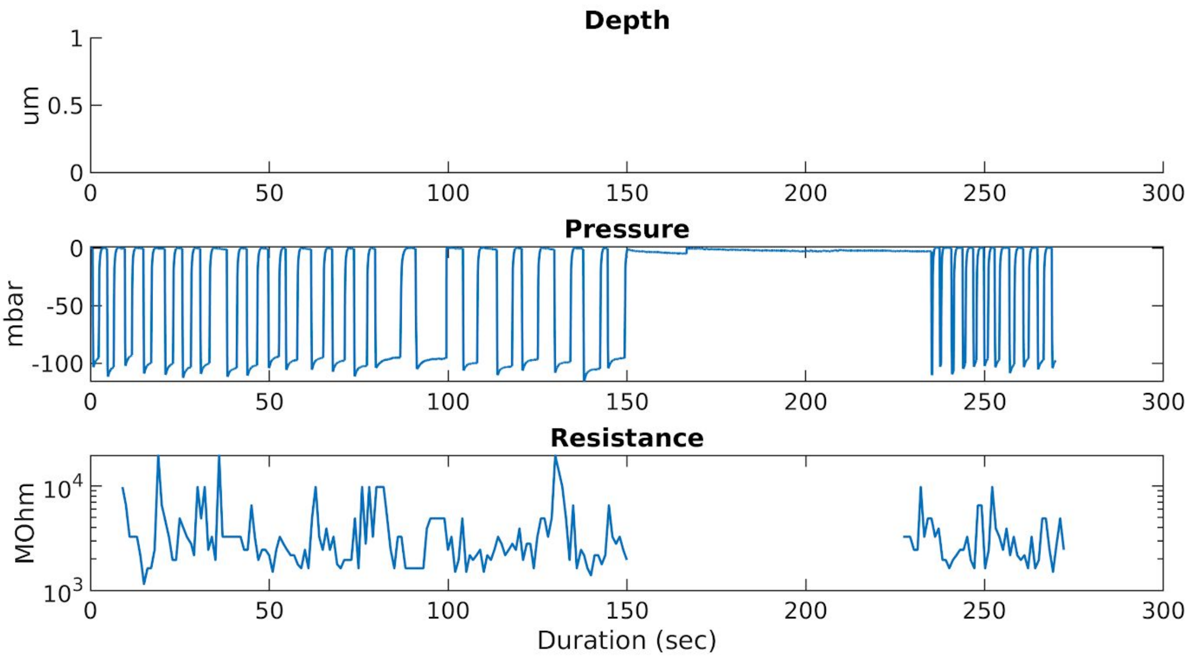

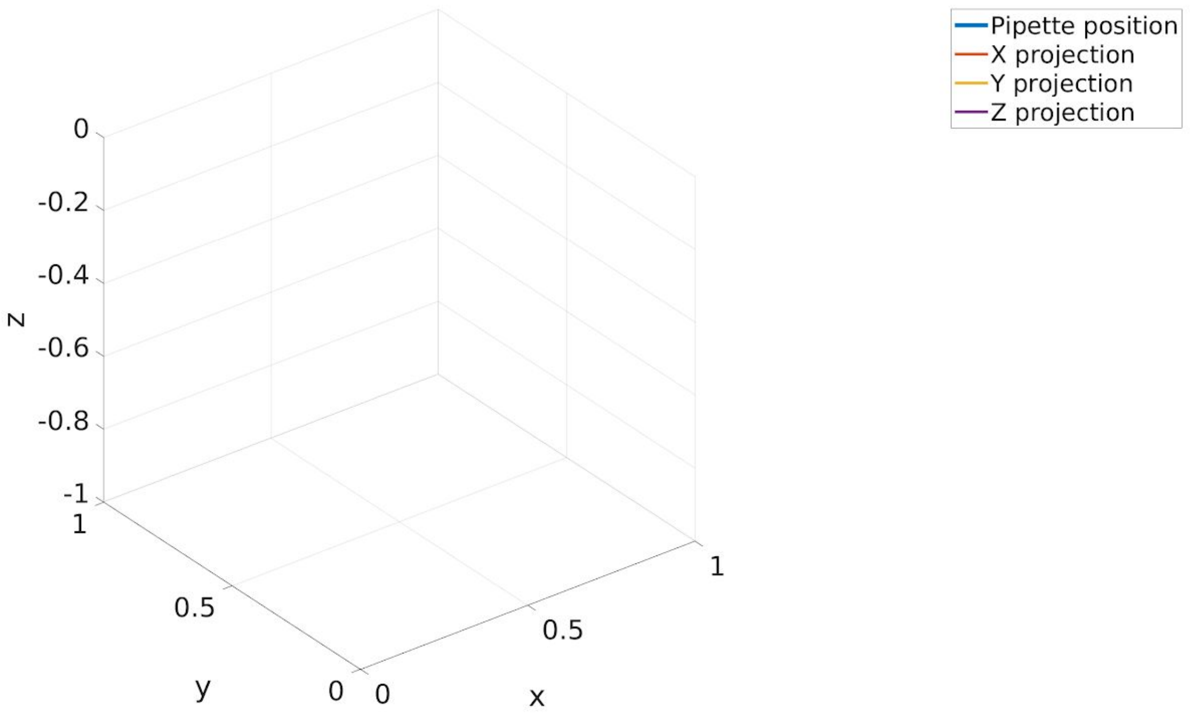

Failed #4

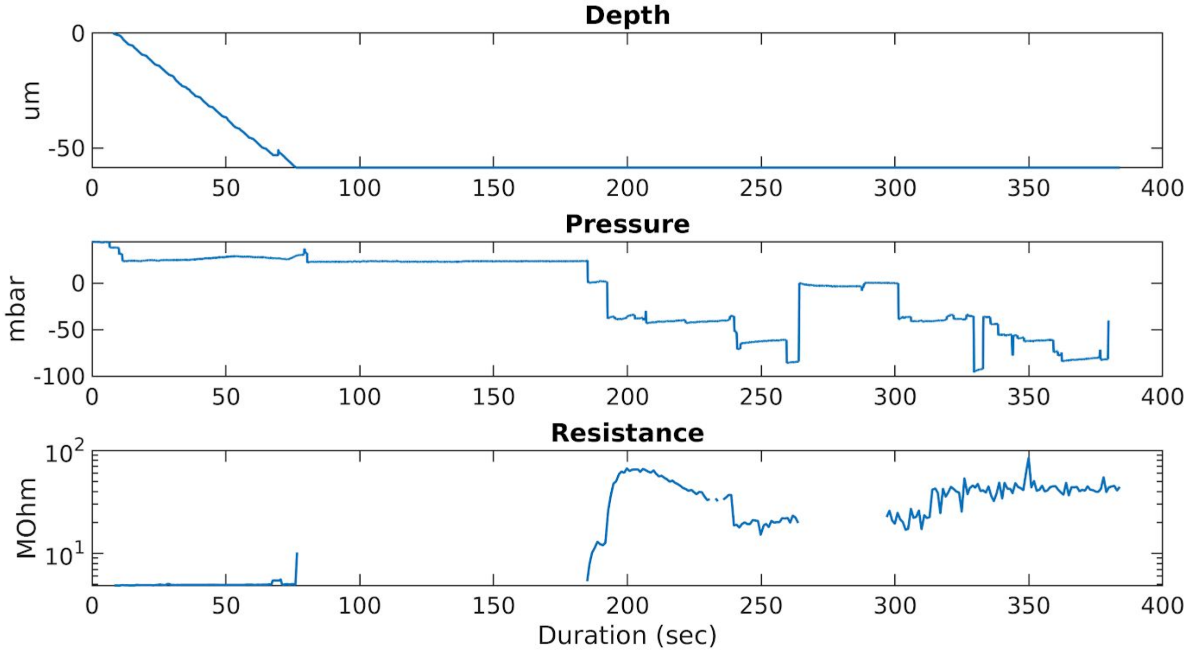

Depth
um
0
-50
Pressure
mbar
0
-50
-100
Resistance
MOhm
10^2
10^1
0 50 100 150 200 250 300 350 400
Duration (sec)

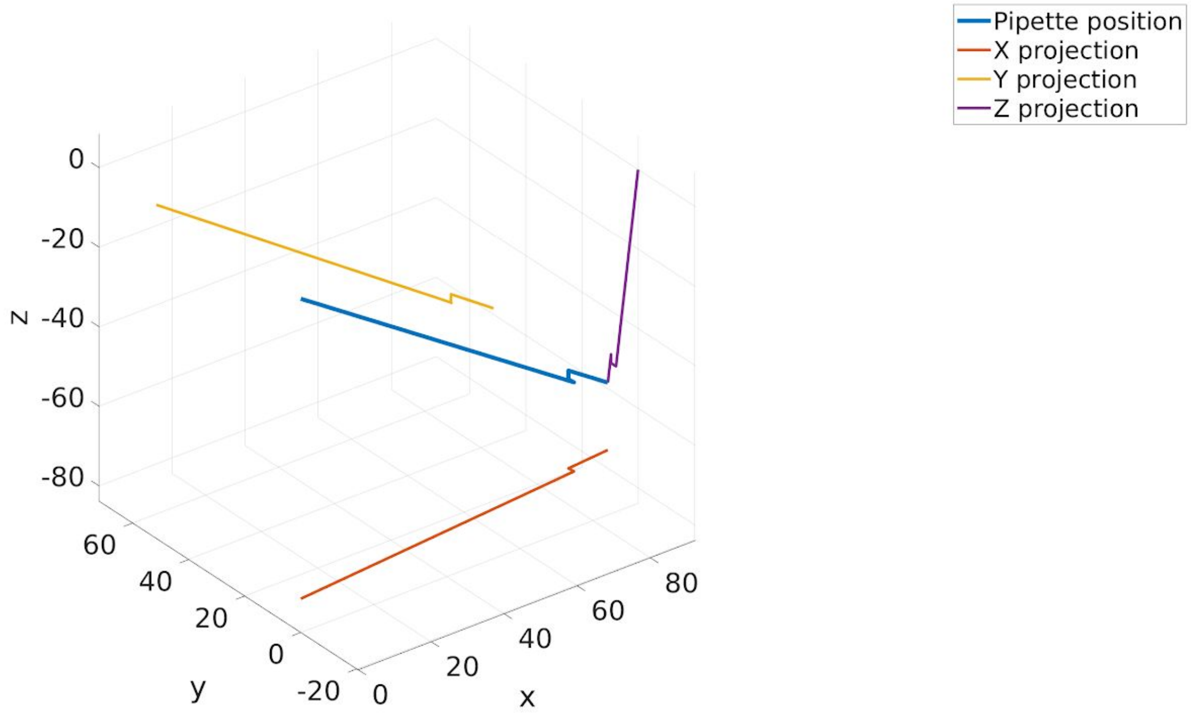

Pipette position
X projection
Y projection
Z projection
z
0
-20
-40
-60
-80
y
60
40
20
0
-20
x
0
20
40
60
80

Failed #5

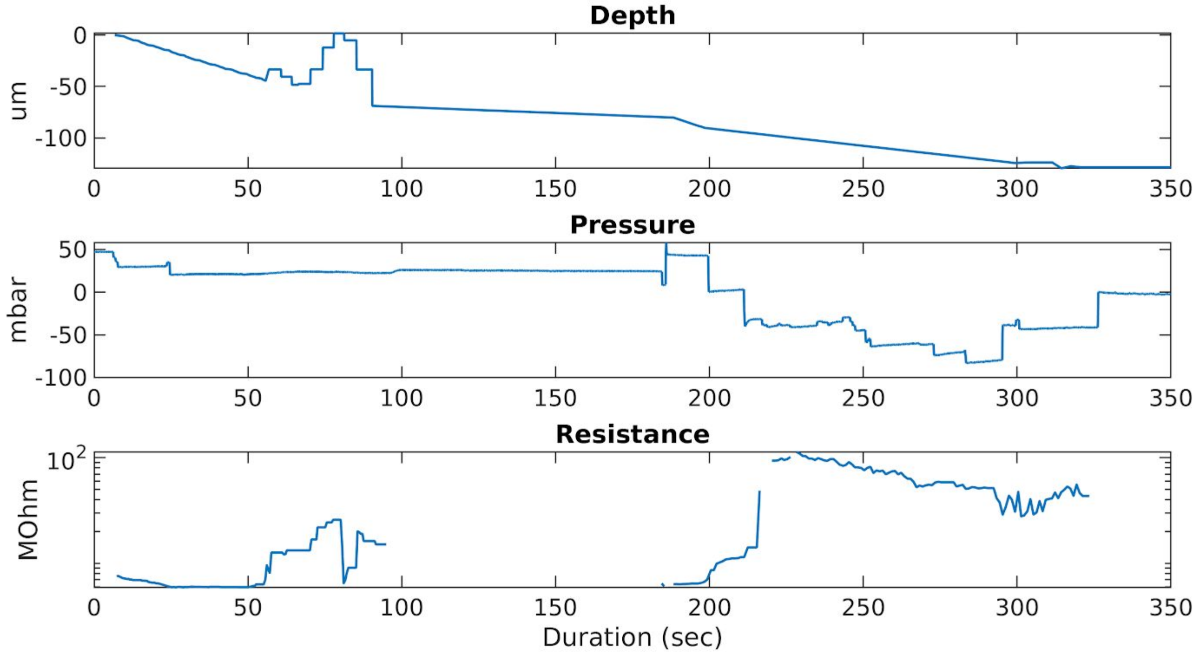
Depth
um
0
-50
-100
Pressure
mbar
50
0
-50
-100
Resistance
MOhm
10^2
0
50
100
150
200
250
300
350
Duration (sec)

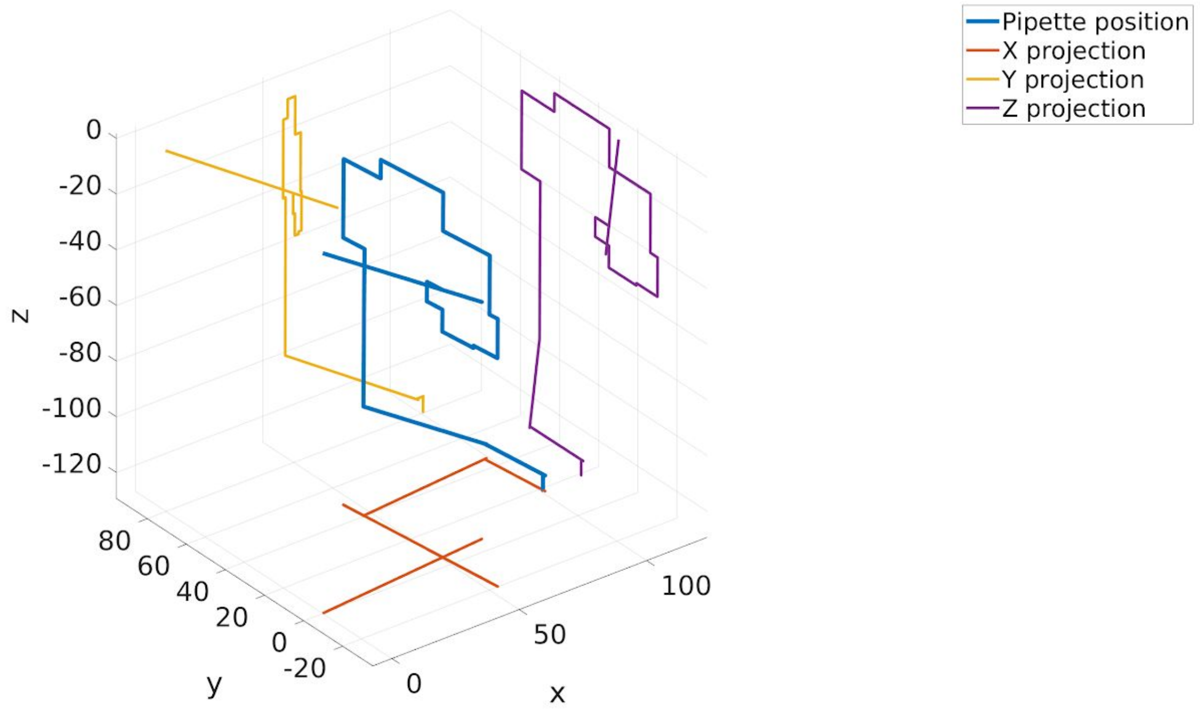
Pipette position
X projection
Y projection
Z projection
z
0
-20
-40
-60
-80
-100
-120
y
80
60
40
20
0
-20
x
0
50
100

Failed #6

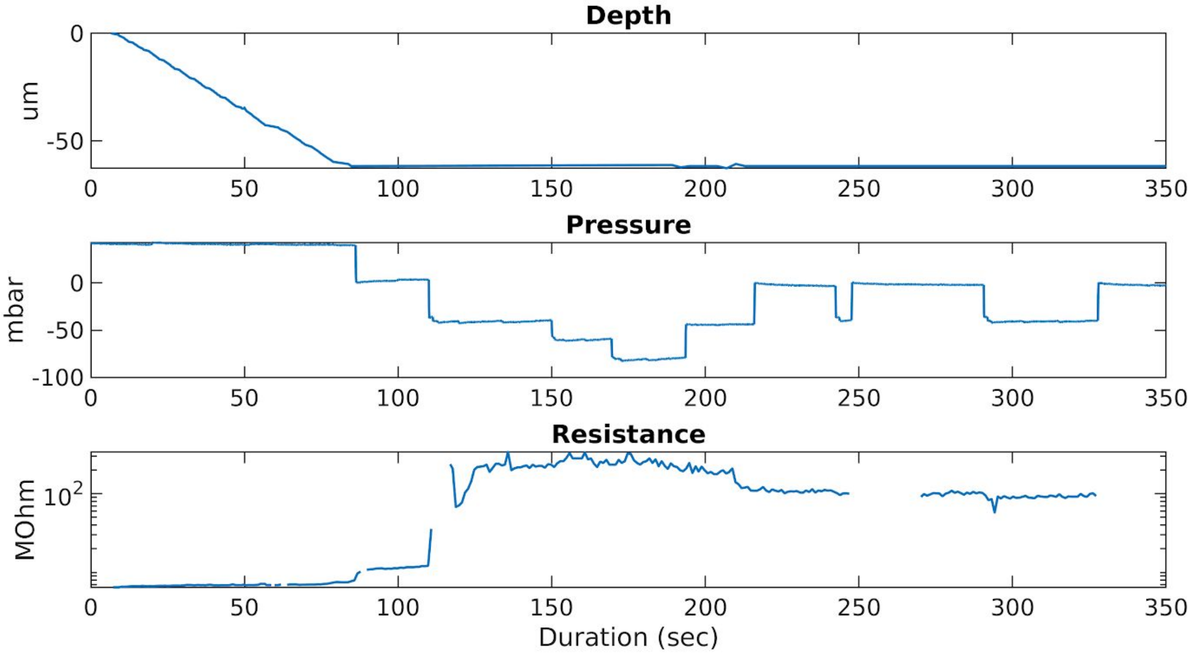

Depth
um
0
-50
Pressure
mbar
0
-50
-100
Resistance
MOhm
10^2
0
50
100
150
200
250
300
350
Duration (sec)

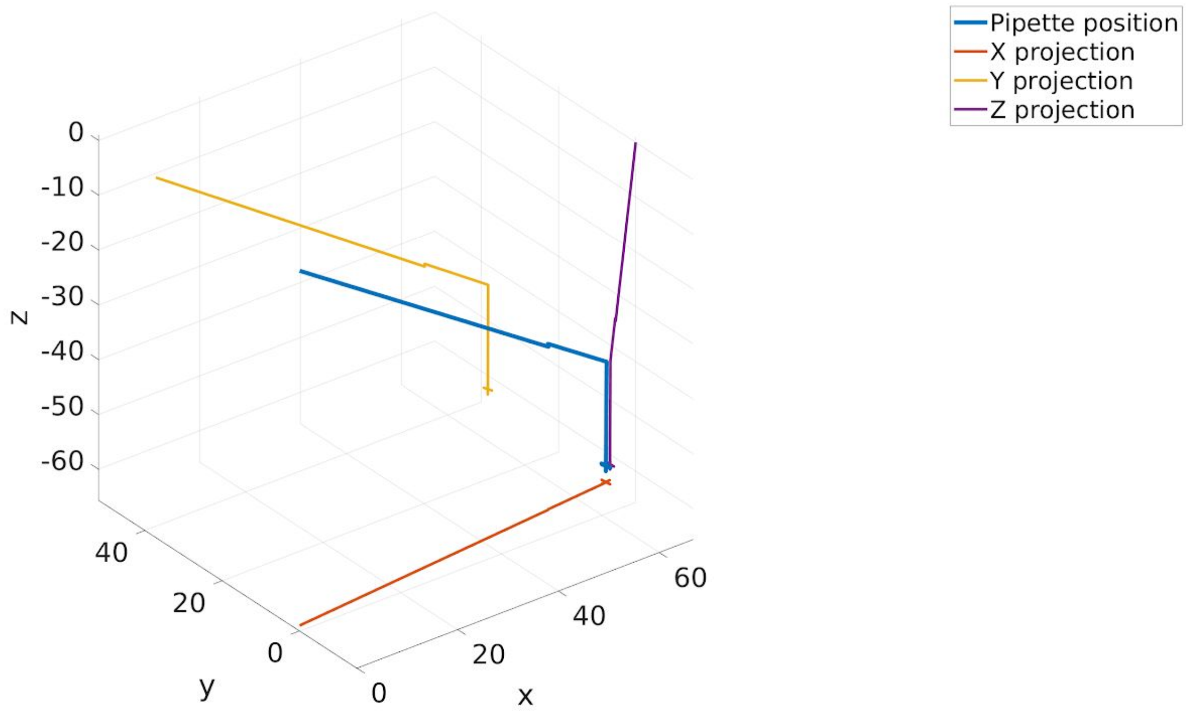

Pipette position
X projection
Y projection
Z projection
z
0
-10
-20
-30
-40
-50
-60
y
40
20
0
x
0
20
40
60

Supplementary Note 8: Anatomical Reconstruction Examples

Example #1

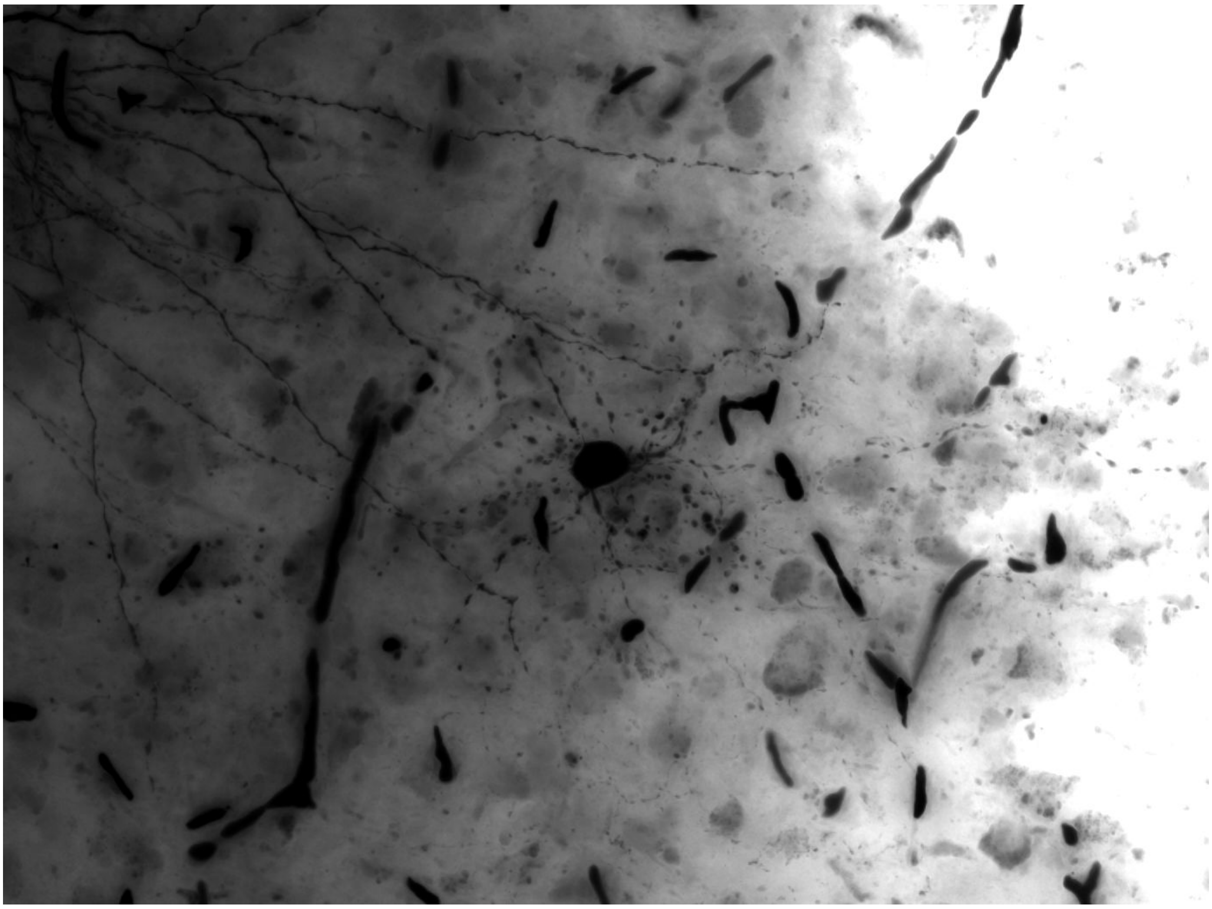

Example #2

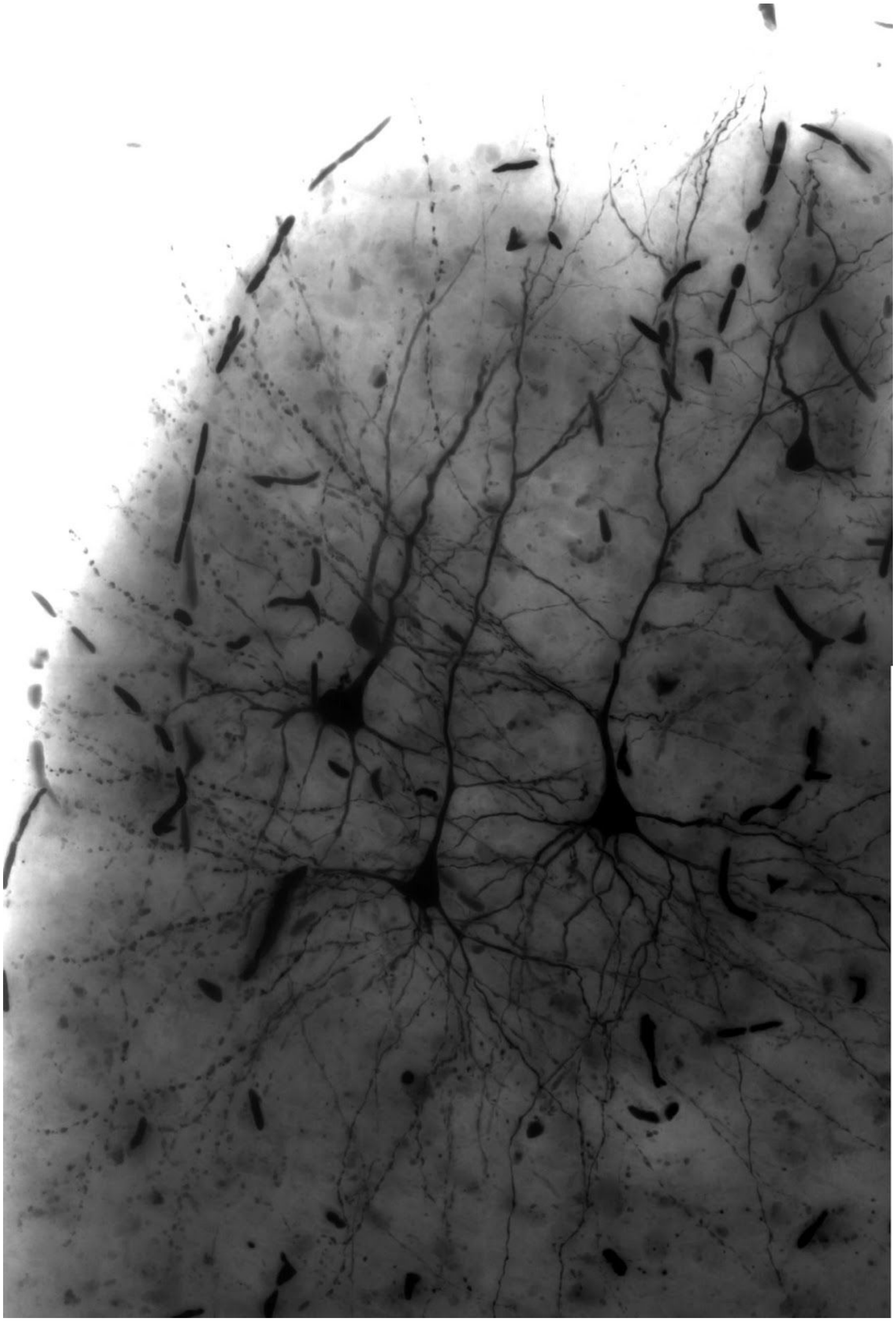

Example #3

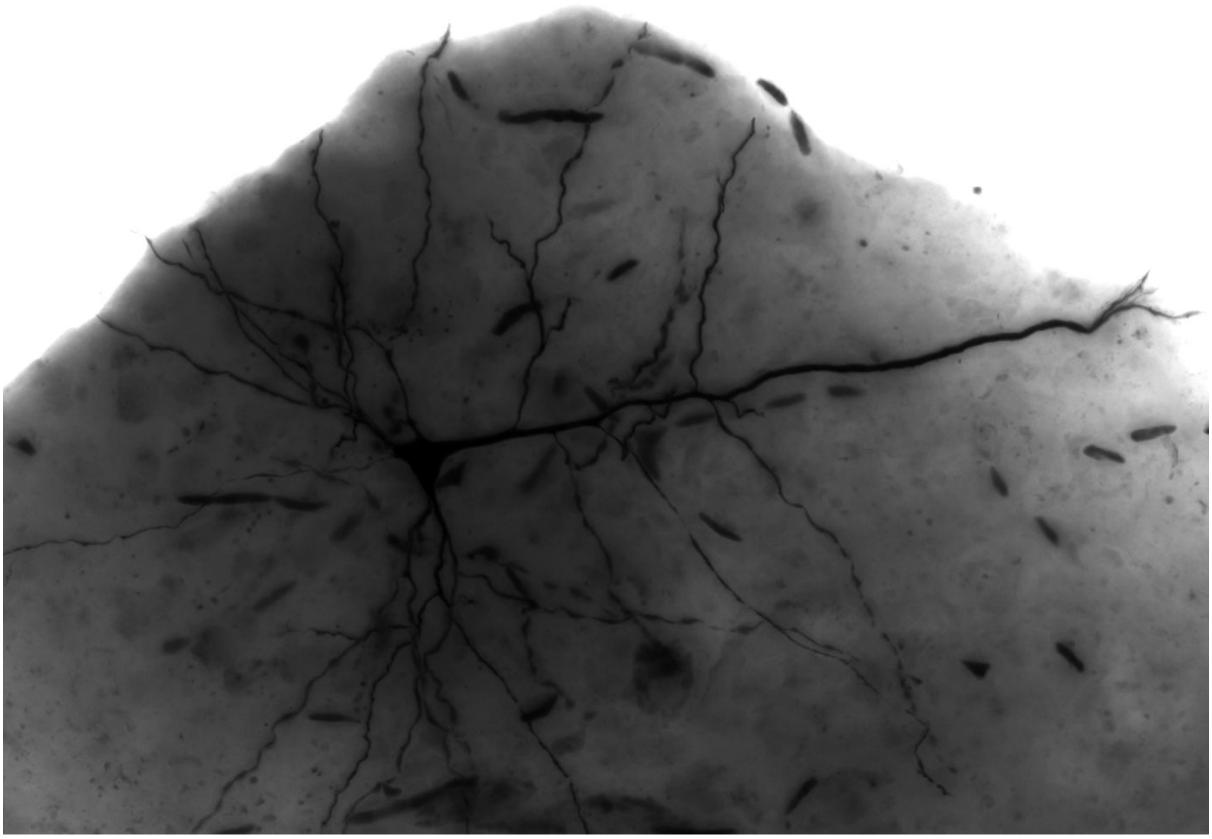

Example #4

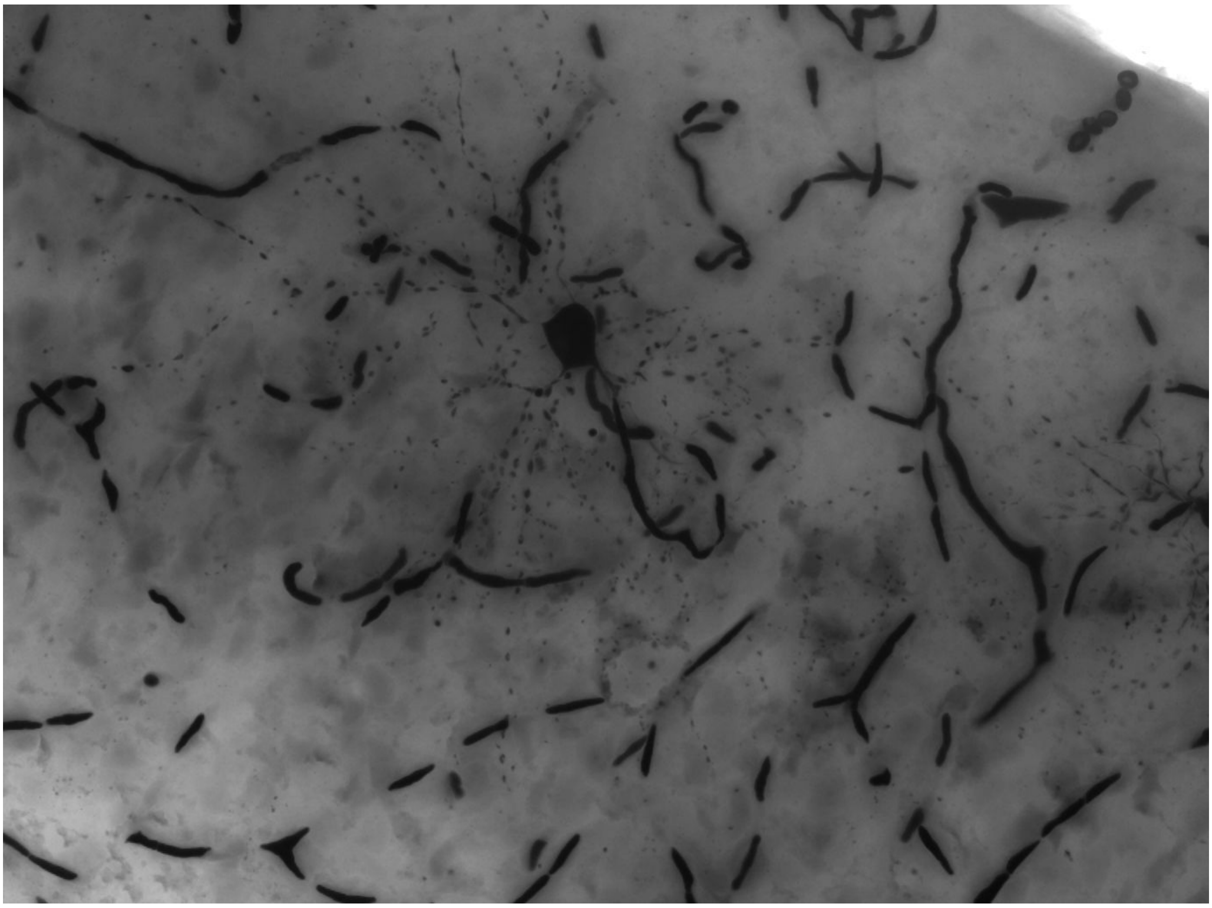

Example #5

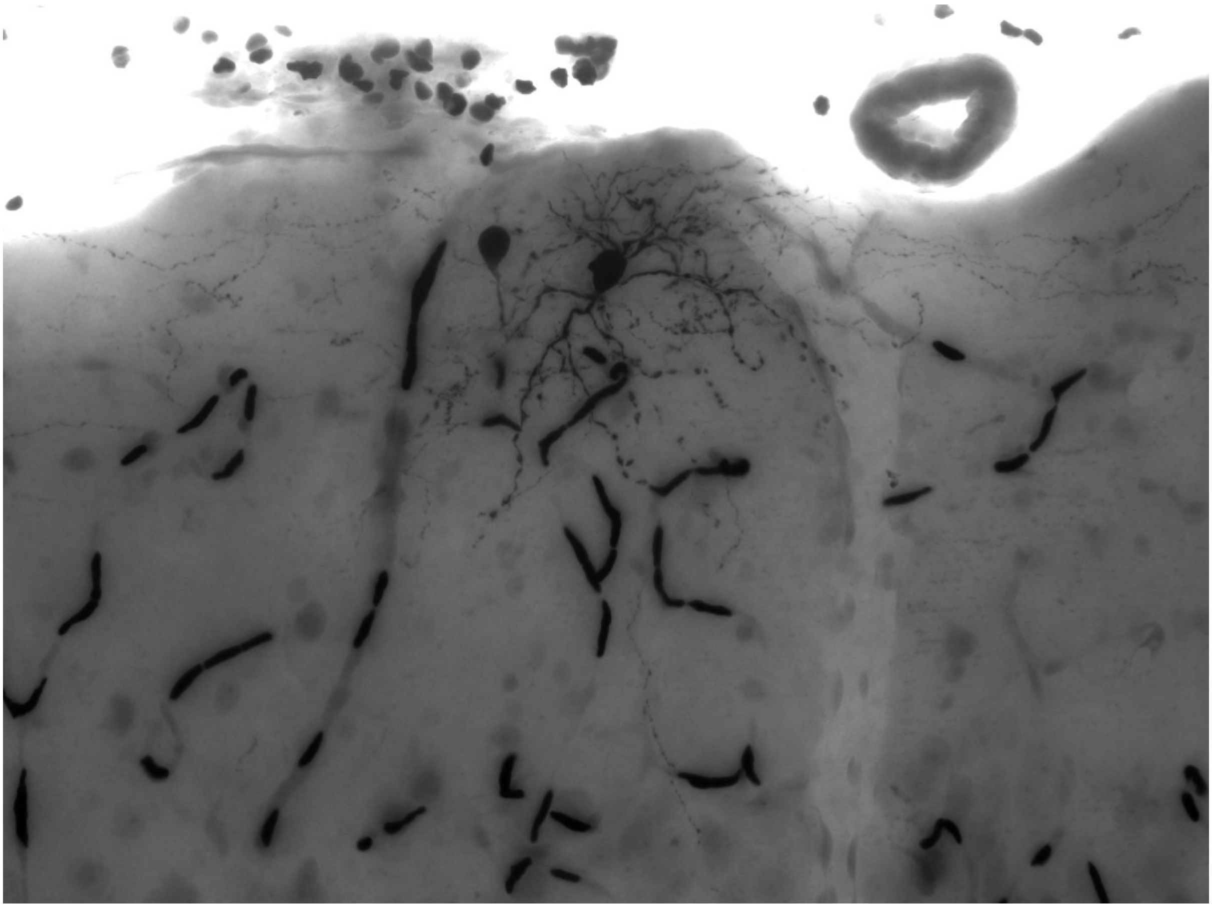

Example #6

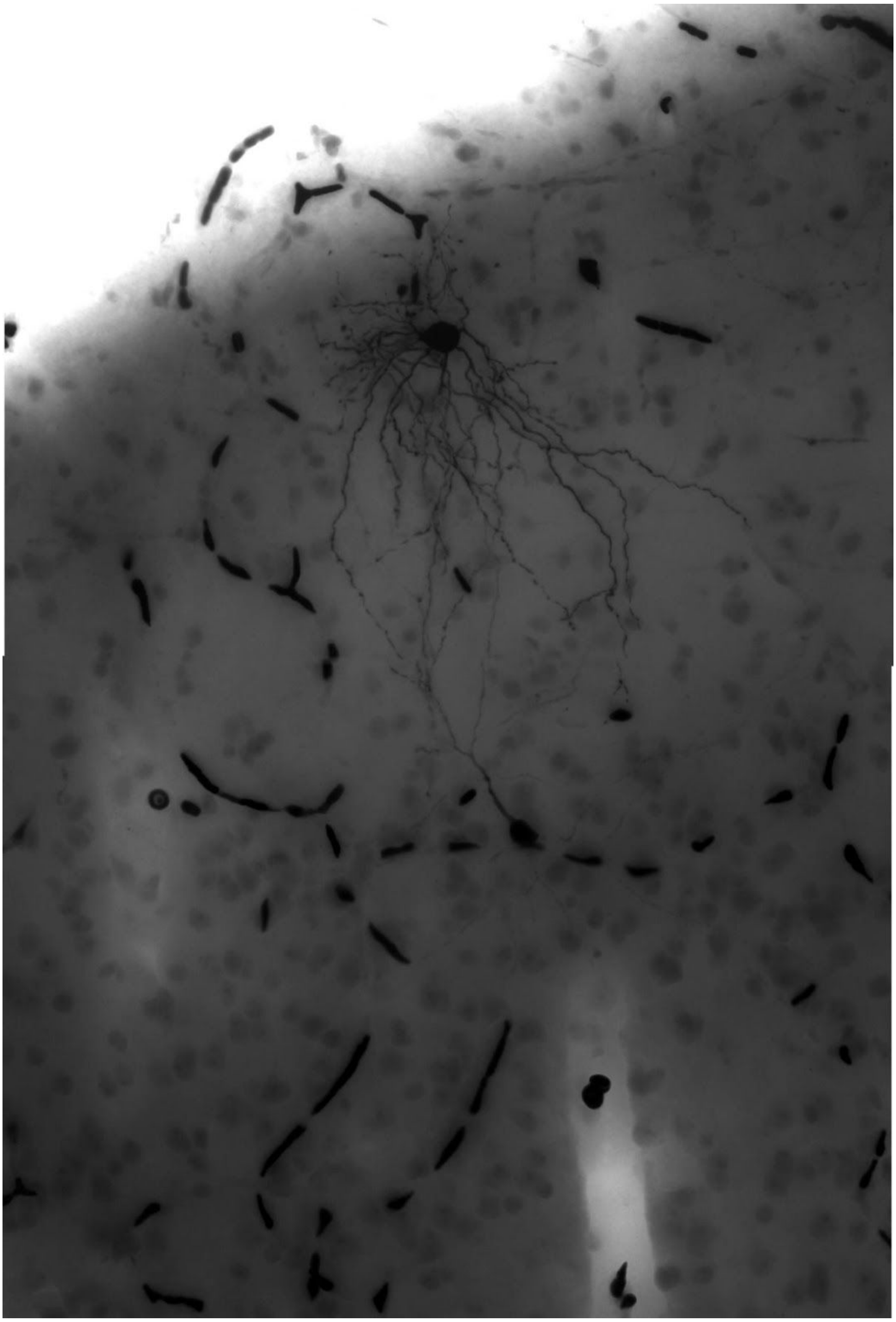

Example #7

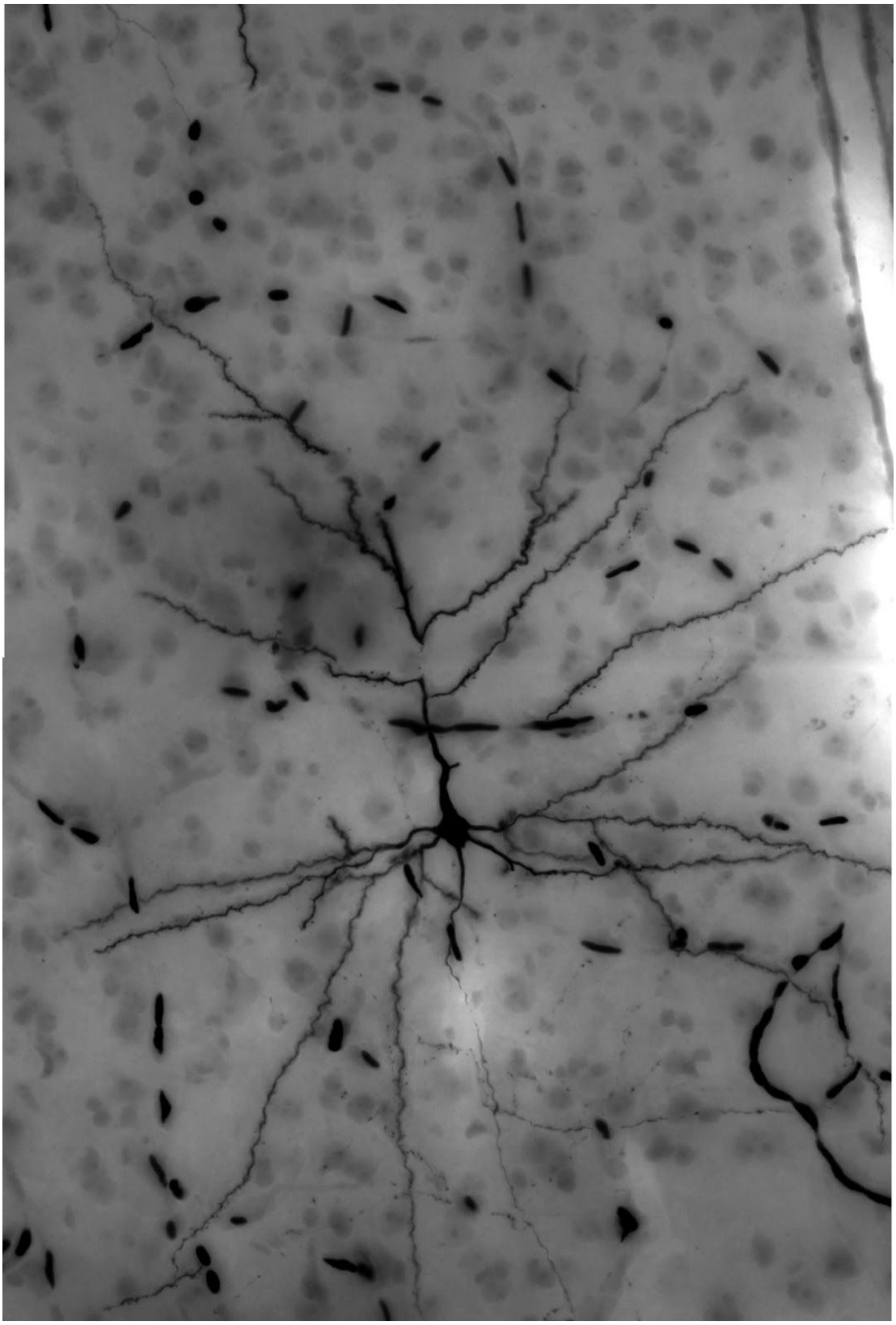

Example #8

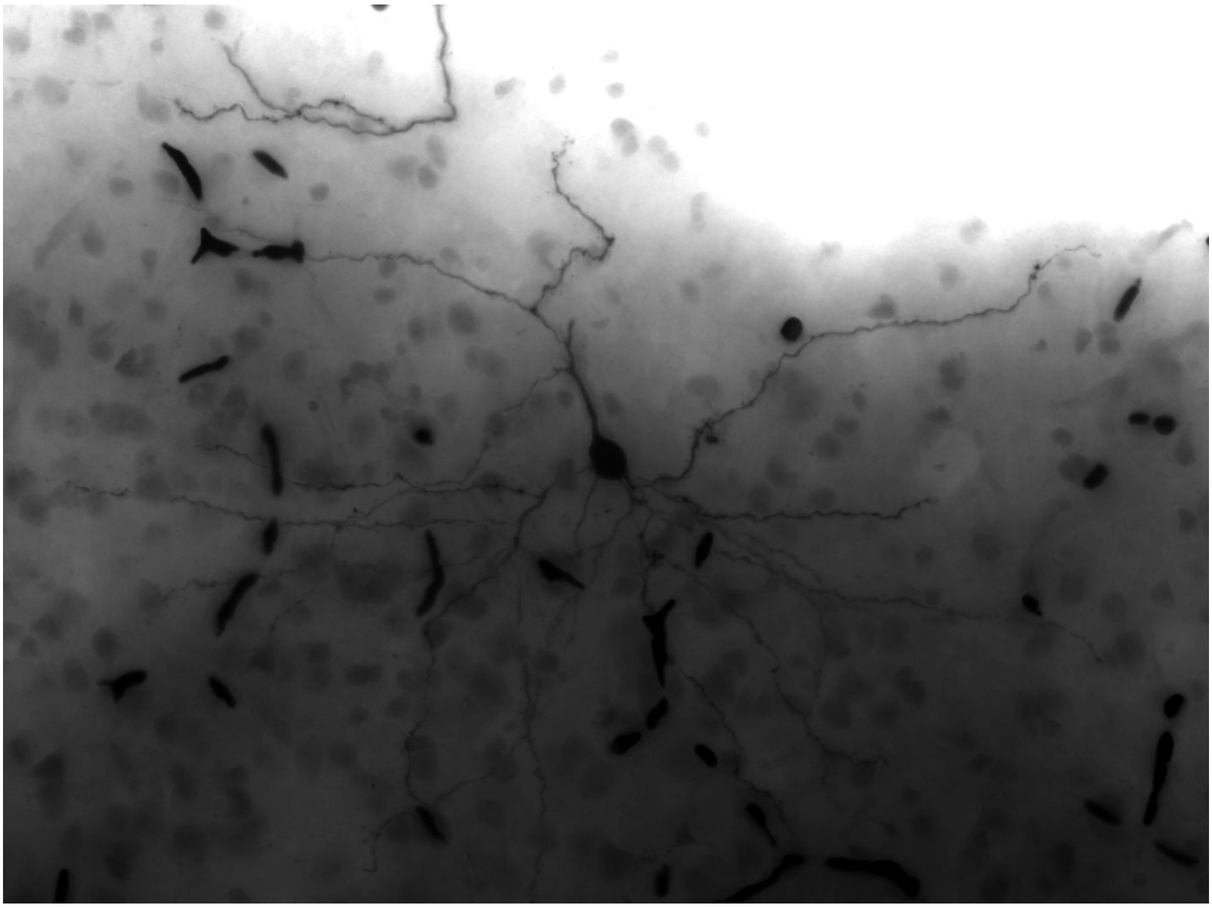

Supplementary References

1. Koos, K., Molnár, J. & Horvath, P. Pipette Hunter: Patch-Clamp Pipette Detection. *Image Analysis* 172–183 (2017) doi:10.1007/978-3-319-59126-1_15.
2. Tao, A., Barker, J. & Sarathy, S. Detectnet: Deep neural network for object detection in digits. *Parallel Forall* **4**, (2016).
3. Szegedy, C. *et al.* Going deeper with convolutions. *2015 IEEE Conference on Computer Vision and Pattern Recognition (CVPR)* (2015) doi:10.1109/cvpr.2015.7298594.
4. Yeager, L., Bernauer, J., Gray, A. & Houston, M. Digits: the deep learning gpu training system. in *ICML 2015 AutoML Workshop* (2015).
5. Jia, Y. *et al.* Caffe. *Proceedings of the ACM International Conference on Multimedia - MM '14* (2014) doi:10.1145/2647868.2654889.
6. Kingma, D. P. & Ba, J. Adam: A Method for Stochastic Optimization. *arXiv [cs.LG]* (2014).
7. Ren, S., He, K., Girshick, R. & Sun, J. Faster R-CNN: Towards Real-Time Object Detection with Region Proposal Networks. *IEEE Trans. Pattern Anal. Mach. Intell.* **39**, 1137–1149 (2017).
8. He, K., Zhang, X., Ren, S. & Sun, J. Deep Residual Learning for Image Recognition. *2016 IEEE Conference on Computer Vision and Pattern Recognition (CVPR)* (2016) doi:10.1109/cvpr.2016.90.
9. Murphy, K. P. *Machine Learning: A Probabilistic Perspective*. (MIT Press, 2012).
10. Paszke, A. *et al.* PyTorch: An Imperative Style, High-Performance Deep Learning Library. in *Advances in Neural Information Processing Systems 32* (eds. Wallach, H. et al.) 8026–8037 (Curran Associates, Inc., 2019).
11. Redmon, J. Darknet: open source neural networks in C (2013--2016). (2016).
12. Redmon, J. & Farhadi, A. YOLOv3: An Incremental Improvement. *arXiv [cs.CV]* (2018).
13. Lin, T.-Y. *et al.* Feature Pyramid Networks for Object Detection. *2017 IEEE Conference on Computer Vision and Pattern Recognition (CVPR)* (2017) doi:10.1109/cvpr.2017.106.
14. Xie, S., Girshick, R., Dollar, P., Tu, Z. & He, K. Aggregated Residual Transformations for Deep Neural Networks. *2017 IEEE Conference on Computer Vision and Pattern Recognition (CVPR)* (2017) doi:10.1109/cvpr.2017.634.
15. Sandler, M., Howard, A., Zhu, M., Zhmoginov, A. & Chen, L.-C. MobileNetV2: Inverted Residuals and Linear Bottlenecks. *2018 IEEE/CVF Conference on Computer Vision and Pattern Recognition* (2018) doi:10.1109/cvpr.2018.00474.
16. Bianco, S., Cadene, R., Celona, L. & Napoletano, P. Benchmark Analysis of Representative Deep Neural Network Architectures. *IEEE Access* vol. 6 64270–64277 (2018).
17. Lucas, B. D., Kanade, T. & Others. An iterative image registration technique with an application to stereo vision. (1981).
18. Tomasi, C. & Kanade, T. Detection and tracking of point features. School of Computer Science. (1991).
19. Stoy, W. A. *et al.* Robotic navigation to subcortical neural tissue for intracellular electrophysiology in vivo. *J. Neurophysiol.* **118**, 1141–1150 (2017).
20. Kolb, I. *et al.* Cleaning patch-clamp pipettes for immediate reuse. *Sci. Rep.* **6**, 35001 (2016).
