# Supplementary material for: Automatic deep learning-driven label-free image-guided patch clamp system
Source: Nat Commun. 2021 Feb 10;12:936. doi: 10.1038/s41467-021-21291-4 (PMC7875980; doi:10.1038/s41467-021-21291-4)
Supplement: Supplementary file 2 — Reporting Summary [file 41467_2021_21291_MOESM2_ESM.pdf]

nature research

Corresponding author(s): Peter Horvath

Last updated by author(s): Jan 11, 2021

Reporting Summary

Nature Research wishes to improve the reproducibility of the work that we publish. This form provides structure for consistency and transparency in reporting. For further information on Nature Research policies, see our Editorial Policies and the Editorial Policy Checklist.

Statistics

For all statistical analyses, confirm that the following items are present in the figure legend, table legend, main text, or Methods section.

n/a	Confirmed	
[ ]	[x]	The exact sample size (n) for each experimental group/condition, given as a discrete number and unit of measurement
[ ]	[x]	A statement on whether measurements were taken from distinct samples or whether the same sample was measured repeatedly
[x]	[ ]	The statistical test(s) used AND whether they are one- or two-sided<br>*Only common tests should be described solely by name; describe more complex techniques in the Methods section.*
[x]	[ ]	A description of all covariates tested
[ ]	[x]	A description of any assumptions or corrections, such as tests of normality and adjustment for multiple comparisons
[ ]	[x]	A full description of the statistical parameters including central tendency (e.g. means) or other basic estimates (e.g. regression coefficient) AND variation (e.g. standard deviation) or associated estimates of uncertainty (e.g. confidence intervals)
[x]	[ ]	For null hypothesis testing, the test statistic (e.g. F, t, r) with confidence intervals, effect sizes, degrees of freedom and P value noted<br>*Give P values as exact values whenever suitable.*
[x]	[ ]	For Bayesian analysis, information on the choice of priors and Markov chain Monte Carlo settings
[x]	[ ]	For hierarchical and complex designs, identification of the appropriate level for tests and full reporting of outcomes
[x]	[ ]	Estimates of effect sizes (e.g. Cohen's d, Pearson's r), indicating how they were calculated

Our web collection on statistics for biologists contains articles on many of the points above.

Software and code

Policy information about availability of computer code

Data collection	Cutom code (https://bitbucket.org/biomag/autopatcher), PatchMaster 2x90.3 versions (HEKA), Matlab (Mathworks)
Data analysis	Custom code, Fitmaster 2x73 (HEKA) and OriginPro 7.5 (OriginLab), Excel 2016 (Microsoft), Matlab R2017a and R2019b (Mathworks), PyTorch 1.4.0

For manuscripts utilizing custom algorithms or software that are central to the research but not yet described in published literature, software must be made available to editors and reviewers. We strongly encourage code deposition in a community repository (e.g. GitHub). See the Nature Research guidelines for submitting code & software for further information.

Data

Policy information about availability of data

All manuscripts must include a data availability statement. This statement should provide the following information, where applicable:

- Accession codes, unique identifiers, or web links for publicly available datasets
- A list of figures that have associated raw data
- A description of any restrictions on data availability

The data shown in Fig. 3, 4, 6, and 7 are provided as a Source Data file. The annotated image data used for deep learning are available from the corresponding author upon request.

Field-specific reporting

Please select the one below that is the best fit for your research. If you are not sure, read the appropriate sections before making your selection.

[x] Life sciences [ ] Behavioural & social sciences [ ] Ecological, evolutionary & environmental sciences

For a reference copy of the document with all sections, see nature.com/documents/nr-reporting-summary-flat.pdf

Life sciences study design

All studies must disclose on these points even when the disclosure is negative.

Sample size	100 out of 157 rat and 74 out of 198 human successful single cell recording were used.
Data exclusions	Successful recordings with Rs value exceeding 100 MΩ were noted as unsuccessful attempts. Successful recordings with less than 30 MΩ were used for further analysis.
Replication	Every protocol used has been described in the main text and in attached supplementary files.
Randomization	The samples and neurons were chosen randomly for the measurements.
Blinding	We used the same algorithms for data collections and analysis from both human and rat samples, therefore blinding was not necessary.

Reporting for specific materials, systems and methods

We require information from authors about some types of materials, experimental systems and methods used in many studies. Here, indicate whether each material, system or method listed is relevant to your study. If you are not sure if a list item applies to your research, read the appropriate section before selecting a response.

Materials & experimental systems

n/a	Involved in the study	
[x]	[ ]	Antibodies
[x]	[ ]	Eukaryotic cell lines
[x]	[ ]	Palaeontology and archaeology
[ ]	[x]	Animals and other organisms
[ ]	[x]	Human research participants
[x]	[ ]	Clinical data
[x]	[ ]	Dual use research of concern

Methods

n/a	Involved in the study	
[x]	[ ]	ChIP-seq
[x]	[ ]	Flow cytometry
[x]	[ ]	MRI-based neuroimaging

Animals and other organisms

Policy information about studies involving animals; ARRIVE guidelines recommended for reporting animal research

Laboratory animals	Male and female wistar rats, age of postnatal 18-25 days.
Wild animals	No animals and other organisms were used.
Field-collected samples	No animals and other organisms were used.
Ethics oversight	University of Szeged Ethics Committee

Note that full information on the approval of the study protocol must also be provided in the manuscript.

Human research participants

Policy information about studies involving human research participants

Population characteristics	The specimens used in the manuscript were from 16 Caucasian donors were selected both genders (n=9 female, aged 48.2 ± 26.6 years and n=7 male, aged 48.3 ± 9.9 years)
Recruitment	We used nonpathological neocortical tissue surgically removed from patients as part of the treatment protocol for epilepsy, hydrocephalus and deep-brain tumors. There was no biased approach used in patient selection criteria.
Ethics oversight	University of Szeged Ethics Committee

Note that full information on the approval of the study protocol must also be provided in the manuscript.
