# Supplementary material for: Automatic deep learning-driven label-free image-guided patch clamp system
Source: Nat Commun. 2021 Feb 10;12:936. doi: 10.1038/s41467-021-21291-4 (PMC7875980; doi:10.1038/s41467-021-21291-4)
Supplement: Supplementary file 7 — Description of Additional Supplementary Files [file 41467_2021_21291_MOESM7_ESM.pdf]

Title: Supplementary Movie 1:
Description: Animated introduction of the DIGAP system.

Title: Supplementary Movie 2:
Description: Screen record of the DIGAP system.

Title: Supplementary Movie 3:
Description: Example patch clamping process from multiple camera angles.

Title: Supplementary Movie 4:
Description: Lateral cell tracking examples.
